# Diastereoselective Synthesis of *N*-Methylspiroindolines by Intramolecular Mizoroki–Heck Annulations

**DOI:** 10.1021/acsomega.2c04111

**Published:** 2022-08-26

**Authors:** Jens Lindman, Greeshma Gopalan, Carlos Palo-Nieto, Peter Brandt, Johan Gising, Mats Larhed

**Affiliations:** Department of Medicinal Chemistry, Uppsala University, Husargatan 3, SE-751 23 Uppsala, Sweden

## Abstract

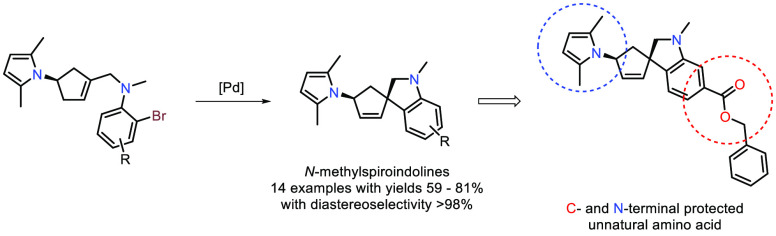

Spiroindolines represent a privileged structure in medicinal chemistry, although stereocontrol around the spirocarbon can be a synthetic challenge. Here we present a palladium(0)-catalyzed intramolecular Mizoroki–Heck annulation reaction from (*+*)-Vince lactam-derived cyclopentenyl-tethered 2-bromo-*N*-methylanilines for the formation of *N*-methylspiroindolines. A series of 14 *N*-methylspiroindolines were synthesized in 59–81% yield with diastereoselectivity >98%, which was rationalized by density functional theory calculations and confirmed through X-ray crystallography. One spiroindoline was converted to an N- and C-terminal protected rigidified unnatural amino acid, which could be orthogonally deprotected.

## Introduction

The spiroindoline scaffold is present in several natural products^1^ and has received interest for use in medicinal chemistry projects with targets related to cancer,^2−4^ antithrombosis,^5^ as well as *in vivo* imaging of neurodegenerative processes/disorders (Supporting Information).^6^ To further improve the utility of these unique scaffolds as tools in biotechnology and drug development projects new synthetic methods to access spiroindolines, preferably with high stereoselectivity, are needed. Several ways of producing spiroindolines exist in the literature, such as through interrupted Fischer indolization,^7^ isomerization/spirocyclization/transfer hydrogenation,^8,9^ photocatalytic [2,2]-addition,^10^ silver^11,12^ and gold^13^ catalyzed cyclization, as well as palladium-catalyzed dearomative ring-closing (Supporting Information).^14^ The Mizoroki–Heck reaction is one of the prominent methods for carbon–carbon bond formation, which is well-suited for utilization in the synthesis of sterically demanding spirocyclic structures, as demonstrated in the synthesis of spirooxindoles,^15−17^ spirobenzofurans,^18^ and spirolactones.^19^ This is due to the tendency of the intramolecular Mizoroki–Heck reaction to selectively yield the *exo* cyclization product upon the intramolecular reaction between an aryl halide and an alkene.^20^ This regioselectivity is observed in the construction of five- or six-membered rings even when the electronic properties of the alkene would suggest a different outcome, as the *exo* route is less sterically demanding. Even though the Mizoroki–Heck reaction has been applied for the synthesis of other spirocycles, palladium-catalyzed spiroindoline synthesis is less-explored. In this work, a 2,5-dimethylpyrrole-protected amine is used as a chiral auxiliary to construct the quaternary all-carbon spirocenter in the intramolecular Mizoroki–Heck synthesis of spiroindolines with high stereocontrol. This ability to achieve facial selectivity of the Mizoroki–Heck annulation through protection of the amine functionality with bulky protecting groups has earlier been investigated^21^ and used in our group for the preparation of spirooxindoles^16,17^ and spirobenzofurans.^18^ The essential chiral starting structure utilized in our new methodology, the stereopure (*+*)-Vince lactam (**1**), has previously garnered attention for its utilization in the synthesis of chiral, substituted cyclopentyl- and pentenyl rings in drug discovery projects. Some prominent examples are the synthesis of abacavir and peramivir, which are used for the treatment of viral diseases.^22^ Rigidified unnatural amino acids are also of importance to drug discovery programs, where they can function either as single molecular entities or be incorporated into proteins or peptides^23,24^ to impart improved pharmacokinetic characteristics, such as higher bioavailability and improved metabolic stability,^25^ of the medicinal compounds.

Herein, we report the synthesis of 14 novel spiroindolines in a diastereo- and regioselective manner through an intramolecular Mizoroki–Heck cyclization of a series of amino group functionalized cyclopentenyl-tethered *N*-methylbromoanilines. Diversification of substrates was achieved through varying the substitution of the anilines used for the spiroindoline synthesis. Through palladium(0)-catalyzed carbonylation chemistry, one compound was converted to an ester-protected unnatural amino acid which was orthogonally deprotected.

## Results and Discussion

**Scheme 1:**
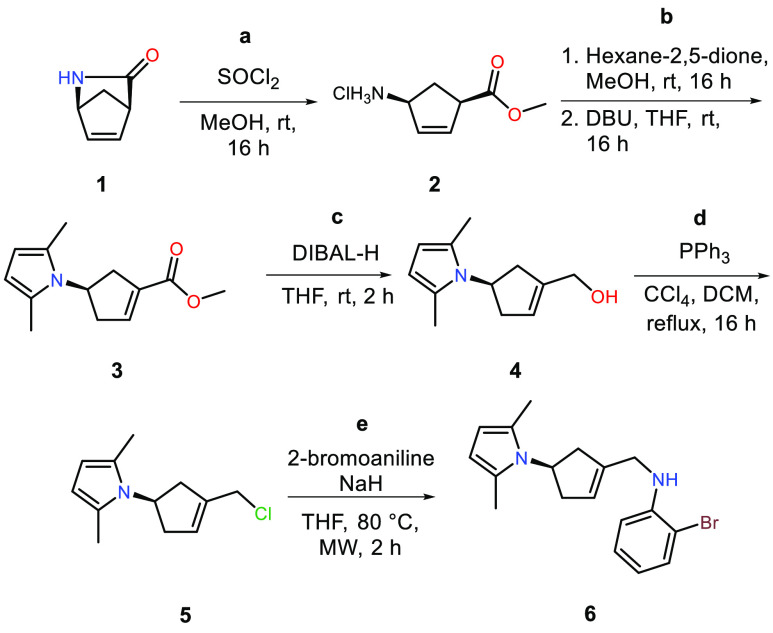
Preparation of *N*-Allylaniline 6 Reaction conditions for **a**: **1** (1 equiv), SOCl_2_ (1.1 equiv), MeOH, 100%. **b**: 1. **2** (1 equiv), hexane-2,5-dione (1 equiv), DIPEA (1 equiv), MeOH. 2. DBU (3 equiv), THF, 85%. **c**: **3** (1 equiv), DIBAL-H (3 equiv), THF, 84%. **d**: **4** (1 equiv), PPh_3_ (1.2 equiv), CCl_4_, DCM, 81%. **e**: **5** (1 equiv), NaH (3 equiv), 2-bromoaniline (2 equiv), THF, microwave heated at 80 °C, 63%.

The study was initiated with the synthesis of *N*-allylaniline cyclization precursor **6** using a literature procedure for the first three synthetic steps (Scheme 1).^21,18^ This was achieved through acid-promoted ring opening of (*1S*)-(+)-2-azabicyclo[2.2.1]hept-5-en-3-one (**1**, (*+*)-Vince lactam) to get the optically pure ammonium chloride salt **2**. The free amine was converted to the corresponding 2,5-dimethylpyrrole, a protecting group strategy chosen due to its proven ability to control the diastereoselectivity of a related intermolecular Mizoroki–Heck alkenylation/arylation of alkene **3**.^21^ The air-sensitive intermediate **2** was without further purification subjected to a DBU-mediated double-bond isomerization to yield acrylic ester **3**. The methyl ester was then reduced using DIBAL-H, followed by conversion of the allyl alcohol **4** to the corresponding allyl chloride **5** via an Appel reaction with triphenylphosphine and carbon tetrachloride. To yield the secondary amine cyclization precursor **6**, allyl chloride **5** was substituted with 2-bromoaniline under microwave heating in a sealed reaction vial and with excess amounts of sodium hydride.

**Scheme 2:**
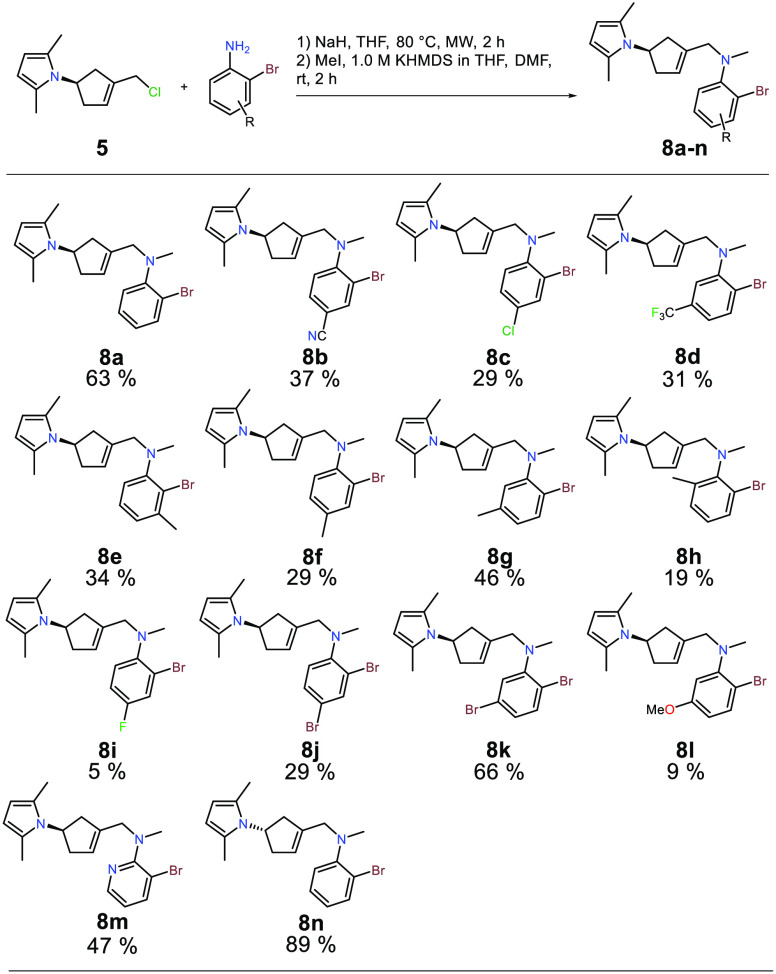
Synthesis of Precursors **8a**–**8n** for Intramolecular Cyclization through Allyl Chloride Substitution Followed by *N*-Methylation Reaction conditions: 1. **5** (1 equiv), aniline (2 equiv), NaH (3 equiv), dry THF, microwave heated at 80 °C. 2. MeI (1.2 equiv), 1.0 M KHMDS in dry THF (1.3 equiv), dry DMF. Isolated yields reported are for two steps (>95% purity as determined by ^1^H NMR).

With substrate **6** in hand, screening of reaction conditions for the Mizoroki–Heck cyclization was commenced. The results are presented in Table 1. A Pd(OAc)_2_/dppf catalytic system with Et_3_N as the base and DMF as the solvent was chosen as the starting point, as this has previously been successfully used for the synthesis of spiroxindoles using a related ring-closing protocol.^16^ Screening of the reaction temperature showed no improvement in conversion when raising the temperature from 80 to 100 °C or 120 °C (entries 1–4). Decreasing dppf loading from 10 to 5 mol % as well as using precatalyst Pd(dppf)Cl_2_ (5 mol %) resulted in a dramatic lowering of the conversion from **6** to **7** (entries 5–7). Our focus was then turned to other palladium ligands, where bis(tri-*tert*-butylphosphine)palladium (Pd(*t*-Bu_3_P)_2_) at higher loading (10 mol %) furnished an improved outcome with >99% conversion after 16 h at 80 °C and an isolated yield of 64% (entry 11). Old-fashioned PPh_3_ did not perform as well as a ligand in the reaction (entry 8), presumably because of the aryl bromide bond being relatively electron-rich due to the electron donating effect of the aniline nitrogen, making oxidative addition sluggish with this ligand.^20^ A couple of control experiments were also conducted, in which omission of palladium afforded <1% conversion (entry 12), and omission of base provided 3% conversion after 16 h (entry 13), thus verifying the necessity of these reaction components. During the optimization attempts, it was observed that both **6** and **7** were somewhat unstable under the reaction conditions, giving rise to unidentified byproducts and low yields, prompting us to investigate if functionalization of the secondary aniline could remedy these issues. Initially, benzylation and acetylation of the secondary aniline nitrogen were attempted, however unsuccessfully. *N*-Methylation of *N*-allylaniline **6** provided a way to synthesize *N*-methylallylaniline **8a** (Scheme 2), which was evaluated with the best-performing conditions from the earlier reaction condition screening. For *N*-methylallylaniline **8a** Pd(*t*-Bu_3_P)_2_ proved effective in catalyzing the reaction, providing >99% conversion after 16 h at 80 °C with an isolated yield of 83% (entry 14). The higher isolated yield with the *N*-methylated substrate (entry 14 compared to entry 11) indicates that our *N*-methylation strategy was successful in improving compound stability. Compared to entry 1, the Pd(OAc)_2_/dppf catalytic system performed relatively poorly with the *N*-methylallylaniline substrate (entry 15).

**Table 1:**
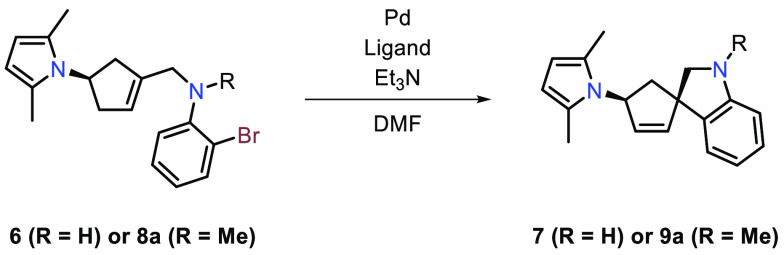
Optimization of Reaction Conditions for Intramolecular Mizoroki–Heck Spirocyclization of **6**/**8a**^a^

entry	starting material	Pd (mol %)	ligand (mol %)	temp (°C)	time (h)	NMR ratio (**6**:**7** or **8a**:**9a**)^b^	isolated yield of **7**/**9a**^c^
1	**6**	Pd(OAc)_2_ (5)	dppf (10)	80	48	11:89	
2	**6**	Pd(OAc)_2_ (5)	dppf (10)	100	16	25:75	
3	**6**	Pd(OAc)_2_ (5)	dppf (10)	120	5	19:81	
4	**6**	Pd(OAc)_2_ (5)	dppf (10)	120	16	22:78	
5	**6**	Pd(OAc)_2_ (5)	dppf (5)	80	5	83:17	
6	**6**	Pd(OAc)_2_ (5)	dppf (5)	80	16	82:18	
7	**6**	Pd(dppf)Cl_2_ (5)		80	16	90:10	
8	**6**	Pd(OAc)_2_ (5)	PPh_3_ (10)	80	16	55:45	
9	**6**	Pd(OAc)_2_ (5)	XPhos (10)	80	16	96:4	
10	**6**	Pd(*t*-Bu_3_P)_2_ (5)		80	5	11:89	
11	**6**	Pd(*t*-Bu_3_P)_2_ (10)		80	16	1:99	64%
12	**6**		*t*-Bu_3_P (10)	80	16	100:0	
13^d^	**6**	Pd(*t*-Bu_3_P)_2_ (10)		80	16	97:3	
14	**8a**	Pd(*t*-Bu_3_P)_2_ (10)		80	16	1:99	83%
15	**8a**	Pd(OAc)_2_ (5)	dppf (10)	80	16	47:53	

a Reaction conditions: cyclization precursor **6** or **8a** (0.1 mmol, 1 equiv), Et_3_N (2 equiv), DMF. Amounts of palladium precatalyst and ligands listed in the table.

b Ratio between **6**:**7** or **8a**:**9a** determined by ^1^H NMR of crude reaction mixture.

c Isolated yields after column chromatography.

d Experiment run without Et_3_N. In all screening reactions diastereoselectivity toward the *anti* product was >98% according to ^1^H NMR of the crude reaction mixture.

Rewardingly, in all entries of Table 1, compound **7/9a** was always observed as the diastereomer resulting from a stereocontrolled *anti* migratory insertion. As the secondary *N*-allylaniline starting materials were found to be unstable during preparation, the focus of our inquiry was shifted to *N*-methylallylaniline Mizoroki–Heck substrates. Cyclization precursors **8a**–**8n** based on this concept were prepared in a two-step sequence, where allylic chloride substitution followed by *N*-methylation with methyl iodide and potassium bis(trimethylsilyl)amide (KHMDS) gave the desired **8a**–**8n** in isolated yields ranging between 5–89% over two steps (Scheme 2). Unfortunately, some electron-deficient anilines furnished only low reactivity under the investigated conditions. Allylation of 4-amino-3-bromobenzoic acid and 2-bromo-5-nitroanline led to trace amounts of product formation as seen on LC-MS with allyl chloride **5** still remaining in the reaction mixture. 1-(3-Amino-4-bromophenyl)ethan-1-one afforded only low amounts of product with the partial conversion of allyl chloride **5** but also significant byproduct formation.

**Figure 1:**
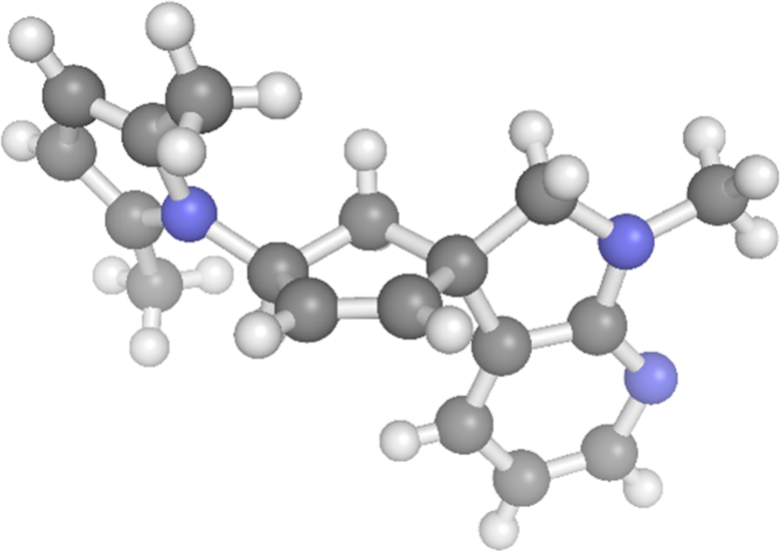
PyMOL visual representation of the X-ray crystallography structure of compound **9m**.

**Scheme 3:**
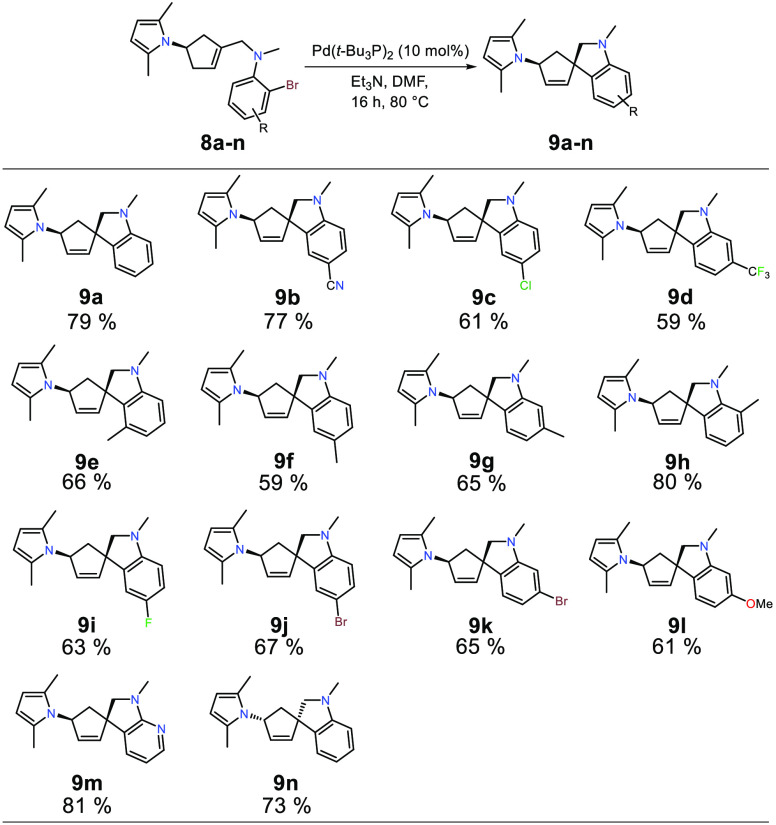
Spirocyclization of *N*-Methylallylanilines **8a**–**8n** Reaction conditions for the synthesis of spiroindolines **9a**–**9n**: cyclization precursor **8a**–**8n** (1 equiv), Pd(*t*-Bu_3_P)_2_ (10 mol %), Et_3_N (2 equiv), DMF. Isolated yields determined after flash chromatography (>95% purity as determined by ^1^H NMR).

Having established the optimum reaction conditions, the scope of the intramolecular Heck–Mizoroki spirocyclization was investigated by varying the substitution of the aniline. The results are presented in Scheme 3. Analogous to what was observed during reaction optimization, only the diastereomer from an *anti* insertion was detected by ^1^H NMR for compounds **9a**–**9n**. All substrates applied in the spirocyclization reaction performed satisfactorily with isolated yields ranging between 59 and 81%. No immediate pattern could be discerned concerning the outcome of electron-poor compared to electron-rich anilines. The chemoselectivity of the reaction was displayed in the synthesis of **9c**, where the chloride was left intact with no signs of dehalogenation, and the compound could be isolated in 61% yield. Methyl-substituted aniline precursors **8e**–**8h** were all well accommodated in the spirocyclization reaction giving moderate to good isolated yields. Interestingly, the 3-position of the aniline could be substituted with a methyl group without the additional steric hindrance around the reactive center negatively effecting the reactivity, with the isolated yield of **9e** comparable to what was achieved for 4-, 5-, and 6-methylanilines **9f**–**9h**. Dibromo substrates **8j** and **8k** afforded the corresponding spirocyclic products in 67% and 65% yield, respectively. However, debromination was detected in the spirocyclization reactions where in both cases debromination was seen only for the bromide in the 4-position (**8j**) and 5-position (**8k**). The debrominated product was in both cases observed as product **9a**, and it is unknown whether this debromination occurs before or after ring closing. The hydride for this dehalogenation might originate from either triethylamine or DMF.^26,27^^1^H NMR analysis of the crude reaction mixture of the cyclization reactions of substrates **8j** and **8k** displayed 9% and 12% debromination, respectively. Aminopyridine **8m** was well-tolerated in the reaction, providing the spirocyclic 7-azaindoline **9m** in a very good isolated yield (81%). Compound **9m** was also subjected to X-ray crystallography studies (CCDC2144389), confirming the *R*-configuration of the spirocarbon in accordance with an *anti* insertion (Figure 1). The enantiomer **9n** was prepared with excellent stereoselectivity and isolated in a yield similar to **9a**. Thus, the results of Scheme 3 could be extrapolated to synthesize any of the enantiomers of compounds **9a**–**9m** starting from (−)-Vince lactam.

**Scheme 4:**
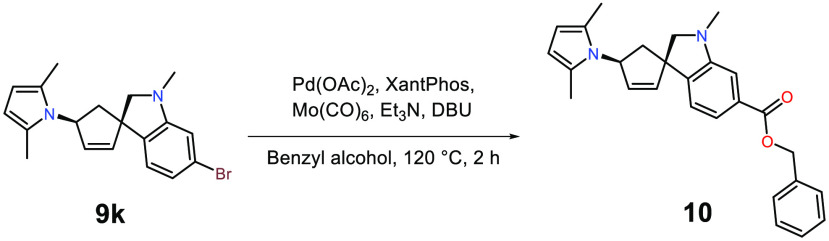
Benzyloxycarbonylation of 5-Bromospiro-indoline **9k** Reaction conditions for **10**: **9k** (1 equiv), Pd(OAc)_2_ (2 mol %), XantPhos (4 mol %), Mo(CO)_6_ (2 equiv), DBU (3 equiv), Et_3_N (2 equiv), and benzyl alcohol used in both chambers, 64%.

In order to access the benzyl-protected C-terminal end of our desired unnatural amino acid, aryl bromide **9k** was subjected to a palladium-catalyzed benzyloxycarbonylation with a PdOAc_2_:XantPhos catalytic system (Scheme 4).^28^ The reaction was run in a two-chamber system, where one chamber is dedicated to the *in situ* production of carbon monoxide from Mo(CO)_6_, which can then diffuse to the other chamber where the carbon monoxide-consuming benzyloxycarbonylation can occur.^29,30^ Employing these conditions, the C- and N-protected unnatural amino acid **10** was synthesized and isolated in 64% yield.

**Scheme 5:**
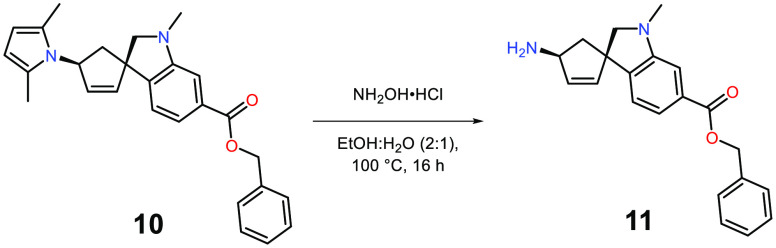
Removal of 2,5-Dimethylpyrrole Protecting Group to Form Free Amine **11a** Reaction conditions for **11**: **10** (1 equiv), NH_2_OH·HCl (10 equiv), EtOH: H_2_O (2:1), 54%.

The 2,5-dimethylpyrrole protected amine **10** was converted to primary amine **11** through a deprotection protocol employing excess amounts of hydroxylamine hydrochloride in a mixture of ethanol and water (Scheme 5).^31^

**Scheme 6:**
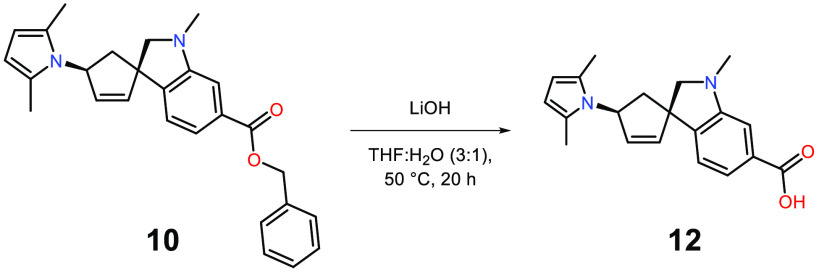
Removal of Benzyl Protecting Group to Form Free Carboxylic Acid 12 Reaction conditions for **12**: **10** (1 equiv), LiOH (5 equiv), THF:H_2_O (3:1), 83%.

For the orthogonal deprotection of the carboxylic acid, hydrolysis of the benzyl ester with LiOH in a THF:water mixture was used to provide the free carboxylic acid in a yield of 83% (Scheme 6).

To further examine the mechanism leading to the diastereoselectivity of the reaction, computational studies were conducted.

**Figure 2:**
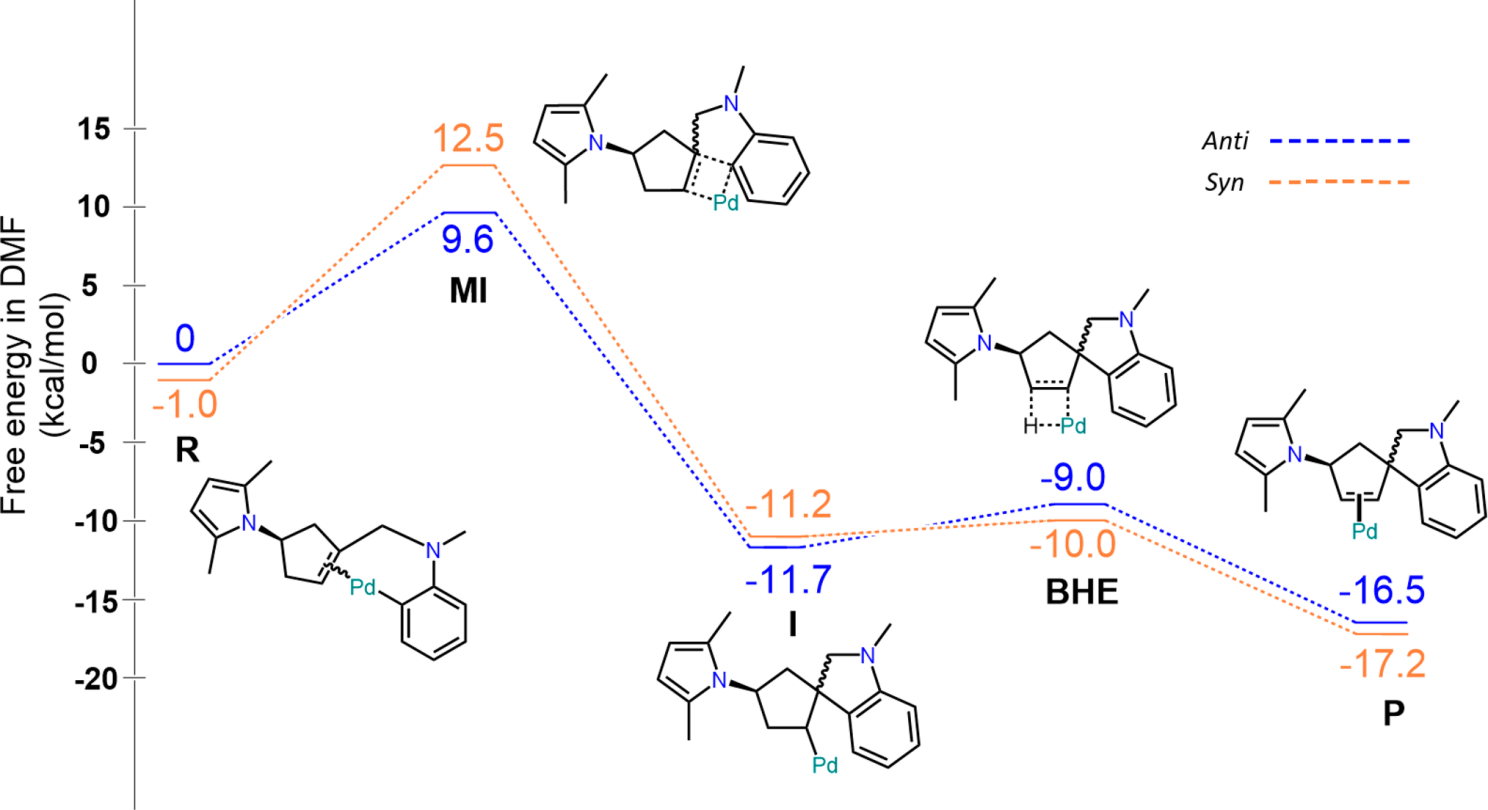
Relative free energies for Mizoroki–Heck reaction intermediates and transition states leading to *syn* and *anti* product calculated by DFT using B3LYP-D3. Additional ligands removed from palladium in the image for clarity. **R** = π complex intermediate, **MI** = migratory insertion transition state, **I** = σ complex intermediate, **BHE** = β-hydride elimination transition state, **P** = product.

**Figure 3:**
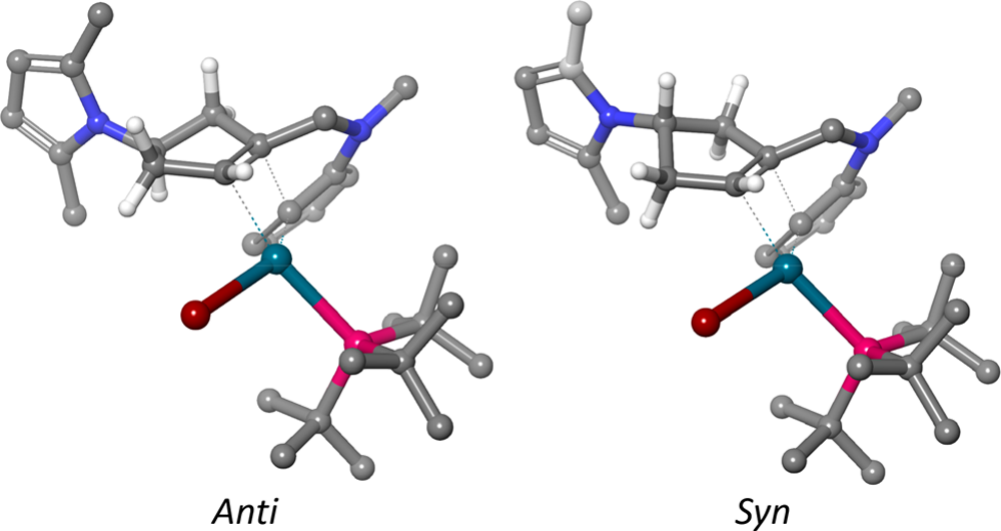
Diastereomeric transition states for migratory insertion illustrating how the 2,5-dimethylpyrrole substituent determines the ring conformation of the cyclopentene. This in turn makes the two trans protons in the top left structure antiperiplanar to the aryl palladium, resulting in a hyperconjugative stabilization of this diastereomeric migratory insertion. Note that the structure to the right is inverted to facilitate the structural comparison. Hydrogen atoms not belonging to the cyclopentenyl ring have been removed from the image for clarity.

Density functional theory calculations were performed for the Mizoroki–Heck annulation of **8a** essentially as described previously for similar systems.^16,21^ The stereoconfiguration of the spirocenter is determined during the migratory insertion transition state (MI). Starting from the aryl-palladium complex, two diastereomeric π-complexes can form. It was found that the *syn* π-complex was slightly more stable than the *anti* π-complex. Assuming a fast equilibrium between the diastereomeric π-complexes, Curtin–Hammett conditions apply,^32^ and the diastereoselectivity will be governed by the energy difference between the two diastereomeric migratory insertions. The energy barrier for the migratory insertion leading to *anti* product **9a** was found to be 2.9 kcal/mol lower than the insertion leading to the *syn* diastereomer (Figure 2, Supporting Information), in line with the Experimental Results. The transition states for the migratory insertion leading to the *anti* and *syn* products are shown in Figure 3.

## Conclusion

A palladium-catalyzed Mizoroki–Heck method employing Pd(*t*-Bu_3_P)_2_ was developed for the diastereoselective spirocyclization of a series of *N*-methylallylanilines to form spiroindolines. This diastereoselectivity was confirmed by X-ray crystallography as well as 2D NOESY. Through a palladium-catalyzed carbonylation protocol, a bromo-substituted spiroindoline was converted to a C- and N-protected unnatural amino acid, where both the amine and carboxylic acid moieties were subsequently orthogonally deprotected.

## Experimental Section

### General Methods

^1^H-, ^13^C-, and ^19^F-NMR as well as 2D-NMR spectra were recorded on a Bruker 400 MHz instrument with chemical shifts reported in parts per million (ppm) and using the residual solvent peak as internal reference. High-resolution mass spectra (HRMS) was recorded using a mass spectrometer with electrospray ionization (ESI) with a 7-T hybrid linear ion trap. Thin-layer chromatography (TLC) with Supelco TLC plates with fluorescence indicator (254 nm) as well as LC/MS on a Thermo Fischer Scientific UltiMate 3000 HPLC system with an MSQ Plus mass spectrometer was used for monitoring of reactions. Manual flash chromatography was run using silica gel (230–400 mesh). Automated flash chromatography was conducted on a Biotage Isolera One flash purification instrument with Biotage Sfär cartridges. Automated reverse phase flash chromatography was performed on a Buchi Reveleris X2 flash chromatography purification system using a Claricep Flash Spherical C18 column. For conventional heating of reactions, DrySyn plates were used as heating mantle. For microwave reactions, the reactions were run in sealed microwave vials using an Anton Paar MonoWave 400 or Biotage Initiator microwave reactor. Optical rotation was recorded on a Rudolph Autopol II polarimeter. Palladium-catalyzed carbonylation reactions were run in two fused microwave vials (H-tubes).

#### (*R*)-1-(3-(Chloromethyl)cyclopent-3-en-1-yl)-2,5-dimethyl-1*H*-pyrrole (**5**)

PPh_3_ (11.9 g, 45.5 mmol) was added to a solution of allyl alcohol **4** (7.25 g, 37.9 mmol) in CCl_4_ (20 mL) and DCM (20 mL). The reaction was refluxed overnight. The reaction mixture was evaporated, and the crude product was purified by automated flash chromatography (5% EtOAc in i-hexane) to yield a light-yellow oil (6.44 g, 30.7 mmol, 81%). ^1^H NMR (400 MHz, acetonitrile-*d*_3_) δ 5.84–5.80 (m, 1H), 5.64 (s, 2H), 5.13–5.03 (m, 1H), 4.30–4.20 (m, 2H), 3.01–2.89 (m, 2H), 2.70–2.55 (m, 2H), 2.18 (s, 6H). ^13^C NMR (101 MHz, acetonitrile-*d*_3_) δ 139.5, 129.1, 128.4, 107.2, 53.4, 44.1, 40.5, 40.4, 13.6. HRMS: calcd. for C_12_H_17_ClN [M + H]^+^ 210.1050; found: 210.1051.

#### (*R*)-*N*-2-Bromo-((4-(2,5-dimethyl-1*H*-pyrrol-1-yl)cyclopent-1-en-1-yl)methyl)aniline (**6**)

To a suspension of sodium hydride (3 equiv, 2.10 mmol) in dry THF (1 mL) was added a dry THF solution (2 mL) of 2-bromoaniline (2 equiv, 1.4 mmol) slowly at 0 °C. After stirring for 30 min, allyl chloride **5** (1 equiv, 0.7 mmol) dissolved in dry THF (2 mL) was added, and the mixture was microwave heated at 80 °C for 2 h. Water (2 mL) was slowly added (pay attention to hydrogen gas evolution), and the product was extracted with EtOAc. The organic solvent was dried with MgSO_4_ and evaporated. The product was isolated by flash chromatography (5–10% EtOAc in iso-hexane) to yield the product **6** as an oil (152.3 mg, 0.44 mmol, 63%). [α]_D_^25^ = +4.7 (*c* = 1.06, THF). ^1^H NMR (400 MHz, DMSO-*d*_6_) δ 7.39 (dd, *J* = 7.8, 1.5 Hz, 1H), 7.13 (ddd, *J* = 8.2, 7.2, 1.5 Hz, 1H), 6.68 (dd, *J* = 8.2, 1.5 Hz, 1H), 6.51 (ddd, *J* = 7.8, 7.2, 1.5 Hz, 1H), 5.56 (s, 2H), 5.54–5.51 (m, 1H), 5.45 (t, *J* = 6.1 Hz, 1H), 5.02–4.91 (m, 1H), 3.95–3.82 (m, 2H), 2.91–2.75 (m, 2H), 2.53–2.38 (m, 2H), 2.07 (s, 6H). ^13^C NMR (101 MHz, acetonitrile-*d*_3_) δ 146.1, 141.1, 133.2, 129.5, 128.3, 124.6, 118.6, 113.0, 109.9, 107.1, 53.3, 44.4, 41.0, 40.3, 13.6. HRMS: calcd. for C_18_H_22_N_2_Br [M + H]^+^ 345.0966; found: 345.0974.

#### (1*R*,4*S*)-4-(2,5-Dimethyl-1*H*-pyrrol-1-yl)spiro[cyclopentane-1,3′-indolin]-2-ene (**7**)

In an oven-dried reaction vial, Pd(*t*-Bu_3_)_2_ (13.0 mg, 25.4 μmol) and Et_3_N (50.8 mg, 70 μL, 0.502 mmol) were taken together. The vial was evacuated and flashed with nitrogen three times. DMF (1 mL) was introduced in the reaction vial, and the solution was allowed to stir at room temperature for 5 min. Afterward, **6** (86.7 mg, 0.251 mmol) in 1 mL of DMF was introduced in the reaction mixture and stirred for 16 h at 80 °C. After completion of the reaction, the reaction mixture was quenched with water (5 mL), and the product was extracted with ethyl acetate (3 × 10 mL). The combined organic layer was washed with a saturated solution of brine and dried over anhydrous MgSO_4_, evaporated, and purified by column chromatography (0–12% EtOAc in pentane) to yield the product as an oil (42.5 mg, 0.160 mmol, 64%). [α]_D_= −16.76 (c= 0.99, THF). ^1^H NMR (400 MHz, DMSO-*d*_6_) δ 7.01–6.91 (m, 2H), 6.58 (td, *J* = 7.4, 1.0 Hz, 1H), 6.54–6.50 (m, 1H), 5.93 (dd, *J* = 5.5, 1.9 Hz, 1H), 5.88 (dd, *J* = 5.5, 2.5 Hz, 1H), 5.61–5.54 (m, 4H), 3.59 (dd, *J* = 9.3, 2.0 Hz, 1H), 3.41 (dd, *J* = 9.3, 2.1 Hz, 1H), 2.64 (dd, *J* = 13.4, 8.5 Hz, 1H), 2.19 (s, 6H), 1.99 (dd, *J* = 13.4, 8.1 Hz, 1H). ^13^C NMR (101 MHz, DMSO-*d*_6_) δ 151.1, 137.4, 134.7, 131.1, 127.6, 127.2, 122.4, 117.3, 108.9, 106.0, 60.1, 59.3, 57.4, 46.5, 13.8. HRMS: calcd. for C_18_H_21_N_2_ [M + H]^+^ 265. 1705; found: 265.1710.

General procedure 1 for the synthesis of *N*-methylallylanilines **8a**–**8n**. To a suspension of sodium hydride (3 equiv) in dry THF (1 mL) was added a dry THF solution (2 mL) of aniline (2 equiv) slowly at 0 °C. After stirring for 30 min, allyl chloride **5** (1 equiv, 0.7 mmol) was added, and the mixture was microwave heated at 80 °C for 2 h. Water (2 mL) was slowly added (pay attention to hydrogen gas evolution), and the product was extracted with EtOAc. The organic solvent was dried with MgSO_4_ and evaporated. Automated flash chromatography was run to remove residual aniline from the mixture. The product was dissolved in 3 mL of dry DMF in a 20 mL closed vial with a septum. The vial was purged with nitrogen, and the mixture was cooled to 0 °C on ice. 1.0 M KHMDS in dry THF (1.3 equiv) was added dropwise to the cooled mixture, which was kept for stirring for 30 min. MeI (1.2 equiv) was added dropwise, and the mixture was stirred for 1 h. After completion of the reaction EtOAc (12 mL) was added, and the organic phase was washed with water (3 × 12 mL), followed by brine (12 mL). The organic phase was dried with MgSO_4_, and the solvent was evaporated. The product was isolated by automated flash chromatography.

#### (*R*)-2-Bromo-*N*-((4-(2,5-dimethyl-1*H*-pyrrol-1-yl)cyclopent-1-en-1-yl)methyl)-*N*-methylaniline (**8a**)

Compound **8a** was synthesized according to general procedure 1. After automated flash chromatography (0–4% EtOAc in pentane), the product was isolated as a clear oil (104 mg, 0.29 mmol, 63% over two steps). [α]_D_^25^ = −11.3 (*c* = 0.1, THF). ^1^H NMR (400 MHz, acetonitrile-*d*_3_) δ 7.56 (dd, *J* = 7.9, 1.5 Hz, 1H), 7.29 (ddd, *J* = 8.0, 7.2, 1.5 Hz, 1H), 7.20 (dd, *J* = 8.0, 1.6 Hz, 1H), 6.93 (td, *J* = 7.9, 7.2, 1.6 Hz, 1H), 5.67–5.60 (m, 3H), 5.00 (m, 1H), 3.78–3.71 (m, 1H), 3.67–3.59 (m, 1H), 2.92–2.79 (m, 2H), 2.70 (s, 3H), 2.60–2.48 (m, 2H), 2.10 (s, 6H). ^13^C NMR (101 MHz, acetonitrile-*d*_3_) δ 152.0, 141.1, 134.6, 129.2, 128.2, 126.8, 125.5, 123.5, 120.9, 107.1, 57.0, 53.5, 42.2, 41.3, 40.1, 13.6. HRMS: calcd. for C_19_H_24_BrN_2_ [M + H]^+^ 359.1123; found: 359.1127.

#### (*R*)-3-Bromo-4-(((4-(2,5-dimethyl-1*H*-pyrrol-1-yl)cyclopent-1-en-1-yl)methyl)(methyl)amino)benzonitrile (**8b**)

Compound **8b** was synthesized according to general procedure 1. After automated flash chromatography (0–20% EtOAc in pentane), the product was isolated as an oil (132 mg, 0.343 mmol, 37% over two steps). [α]_D_^25^ = −1.0 (c = 0.1, THF). ^1^H NMR (400 MHz, acetonitrile-*d*_3_) δ 7.89 (d, *J* = 1.7 Hz, 1H), 7.60 (dd, *J* = 8.5, 1.7 Hz, 1H), 7.20 (d, *J* = 8.5 Hz, 1H), 5.68–5.64 (m, 1H), 5.61 (s, 2H), 5.06–4.96 (m, 1H), 3.91 (d, *J* = 15.1 Hz, 1H), 3.80 (d, *J* = 15.1 Hz, 1H), 2.92–2.76 (m, 5H), 2.60–2.44 (m, 2H), 2.11 (s, 6H). ^13^C NMR (101 MHz, acetonitrile-*d*_3_) δ 156.0, 140.1, 138.5, 133.2, 128.3, 127.4, 122.8, 118.9, 118.1, 107.1, 106.6, 56.1, 53.5, 41.4, 41.1, 40.1, 13.6. HRMS: calcd. for C_20_H_23_BrN_3_ [M + H]^+^ 384.1075; found: 384.1061.

#### (*R*)-2-Bromo-4-chloro-*N*-((4-(2,5-dimethyl-1*H*-pyrrol-1-yl)cyclopent-1-en-1-yl)methyl)-*N*-methylaniline (**8c**)

Compound **8c** was synthesized according to general procedure 1. After flash chromatography (0–10% EtOAc in pentane), the product was isolated as an oil (81.2 mg, 0.206 mmol, 29% over two steps). [α]_D_^25^ = −14.3 (*c* = 0.1, THF). ^1^H NMR (400 MHz, Acetonitrile-*d*_3_) δ 7.60 (d, *J* = 2.5 Hz, 1H), 7.30 (dd, *J* = 8.7, 2.5 Hz, 1H), 7.16 (d, *J* = 8.7 Hz, 1H), 5.66–5.59 (m, 3H), 5.05–4.94 (m, 1H), 3.73 (d, *J* = 14.5 Hz, 1H), 3.62 (d, *J* = 14.5 Hz, 1H), 2.90–2.78 (m, 2H), 2.68 (s, 3H), 2.58–2.46 (m, 2H), 2.09 (s, 6H). ^13^C NMR (101 MHz, acetonitrile-*d*_3_) δ 151.0, 140.7, 133.83, 129.1, 129.0, 128.3, 127.1, 124.4, 121.3, 107.1, 56.8, 53.4, 42.2, 41.2, 40.1, 13.6. HRMS: calcd. for C_19_H_23_N_2_ClBr [M + H]^+^ 393.0733; found: 393.0734.

#### (*R*)-2-Bromo-*N*-((4-(2,5-dimethyl-1*H*-pyrrol-1-yl)cyclopent-1-en-1-yl)methyl)-*N*-methyl-5-(trifluoromethyl)aniline (**8d**)

Compound **8d** was synthesized according to general procedure 1. After flash chromatography (0–10% EtOAc in pentane), the product was isolated as an oil (40.0 mg, 0.094 mmol, 31% over two steps). [α]_D_^25^ = −26.0 (c = 0.1, THF). ^1^H NMR (400 MHz, acetonitrile-*d*_3_) δ 7.73 (dd, *J* = 8.3, 0.7 Hz, 1H), 7.44 (d, *J* = 1.9 Hz, 1H), 7.21 (dd, *J* = 1.9 Hz, 1H), 5.68–5.64 (m, 1H), 5.60 (s, 2H), 5.05–4.94 (m, 1H), 3.88–3.80 (m, 1H), 3.73–3.66 (m, 1H), 2.91–2.78 (m, 2H), 2.76 (s, 3H), 2.58–2.43 (m, 2H), 2.05 (s, 6H). ^13^C NMR (101 MHz, acetonitrile-*d*_3_) δ 152.6, 140.4, 135.6, 130.8 (q, *J* = 32.5 Hz), 128.3, 127.4, 125.1 (q, *J* = 271.0 Hz), 124.8 (q, *J* = 1.5 Hz), 121.6 (q, *J* = 3.7 Hz), 120.1 (q, *J* = 3.6 Hz), 107.1, 56.4, 53.4, 42.2, 41.2, 40.1, 13.5. HRMS: calcd. for C_20_H_23_N_2_F_3_Br [M + H]^+^ 427.0997; found: 427.1012.

#### (*R*)-2-Bromo-*N*-((4-(2,5-dimethyl-1*H*-pyrrol-1-yl)cyclopent-1-en-1-yl)methyl)-*N*,3-dimethylaniline (**8e**)

Compound **8e** was synthesized according to general procedure 1. After flash chromatography (0–10% EtOAc in pentane), the product was isolated as an oil (114 mg, 0.305 mmol, 34% over two steps). [α]_D_^25^ = −23.01 (*c* = 0.1, THF). ^1^H NMR (400 MHz, acetonitrile-*d*_3_) δ 7.18 (dd, *J* = 7.8, 7.6 Hz, 1H), 7.05 (d, *J* = 7.8 Hz, 1H), 7.00 (d, *J* = 7.6 Hz, 1H), 5.65–5.59 (m, 3H), 5.04–4.94 (m, 1H), 3.71 (d, *J* = 14.2 Hz, 1H), 3.59 (d, *J* = 14.2 Hz, 1H), 2.91–2.79 (m, 2H), 2.66 (s, 3H), 2.60–2.47 (m, 2H), 2.39 (s, 3H), 2.08 (s, 6H). ^13^C NMR (101 MHz, acetonitrile-*d*_3_) δ 152.4, 141.3, 140.5, 128.4, 128.3, 126.8, 126.7, 124.1, 121.0, 107.1, 57.2, 53.5, 42.6, 41.3, 40.1, 24.3, 13.6. HRMS: calcd. for C_20_H_26_N_2_Br [M + H]^+^ 373.1279; found: 373.1285.

#### (*R*)-2-Bromo-*N*-((4-(2,5-dimethyl-1*H*-pyrrol-1-yl)cyclopent-1-en-1-yl)methyl)-*N*,4-dimethylaniline (**8f**)

Compound **8f** was synthesized according to general procedure 1. After flash chromatography (0–10% EtOAc in pentane), the product was isolated as an oil (89.0 mg, 0.238 mmol, 29% over two steps). [α]_D_^25^ = −5.00 (*c* = 0.1, THF). ^1^H NMR (400 MHz, acetonitrile-*d*_3_) δ 7.35–7.31 (m, 1H), 7.04–7.01 (m, 2H), 5.56–5.52 (m, 3H), 4.97–4.86 (m, 1H), 3.62 (d, *J* = 14.3 Hz, 1H), 3.51 (d, *J* = 14.3 Hz, 1H), 2.83–2.71 (m, 2H), 2.58 (s, 3H), 2.51–2.39 (m, 2H), 2.18 (s, 3H), 2.01 (s, 6H). ^13^C NMR (101 MHz, acetonitrile-*d*_3_) δ 149.4, 141.3, 135.7, 134.8, 129.8, 128.3, 126.7, 123.3, 120.9, 107.1, 57.1, 53.5, 42.4, 41.3, 40.1, 20.3, 13.6. HRMS: calcd. for C_20_H_26_N_2_Br [M + H]^+^ 373.1279; found: 373.1279.

#### (*R*)-2-Bromo-*N*-((4-(2,5-dimethyl-1*H*-pyrrol-1-yl)cyclopent-1-en-1-yl)methyl)-*N*,5-dimethylaniline (**8g**)

Compound **8g** was synthesized according to general procedure 1. After flash chromatography (0–10% EtOAc in pentane), the product was isolated as a white solid (139 mg, 0.372 mmol, 46% over two steps). [α]_D_^25^ = −13.33 (*c* = 0.1, THF). ^1^H NMR (400 MHz, acetonitrile-*d*_3_) δ 7.41 (d, *J* = 8.1 Hz, 1H), 7.04 (d, *J* = 1.8 Hz, 1H), 6.76 (dd, *J* = 8.1, 1.8 Hz, 1H), 5.65–5.59 (m, 3H), 5.05–4.95 (m, 1H), 3.77–3.70 (m, 1H), 3.65–3.58 (m, 1H), 2.91–2.79 (m, 2H), 2.68 (s, 3H), 2.59–2.46 (m, 2H), 2.27 (s, 3H), 2.09 (s, 6H). ^13^C NMR (101 MHz, acetonitrile-*d*_3_) δ 151.7, 141.1, 139.4, 134.2, 128.3, 126.7, 126.3, 124.2, 117.4, 107.0, 56.9, 53.5, 42.3, 41.3, 40.1, 21.1, 13.5. HRMS: calcd. for C_20_H_26_N_2_Br [M + H]^+^ 373.1279; found: 373.1283.

#### (*R*)-2-Bromo-*N*-((4-(2,5-dimethyl-1*H*-pyrrol-1-yl)cyclopent-1-en-1-yl)methyl)-*N*,6-dimethylaniline (**8h**)

Compound **8h** was synthesized according to general procedure 1. After flash chromatography (0–10% EtOAc in pentane) the product was isolated as a white solid (110 mg, 0.295 mmol, 19% over two steps). [α]_D_^25^ = −12.31 (c = 0.1, THF). ^1^H NMR (400 MHz, acetonitrile-*d*_3_) δ 7.39 (d, *J* = 7.9 Hz, 1H), 7.16 (d, *J* = 7.7 Hz, 1H), 6.94 (dd, *J* = 7.9, 7.7 Hz, 1H), 5.73–5.68 (m, 1H), 5.63 (s, 2H), 5.08–4.97 (m, 1H), 3.99–3.59 (m, 2H), 2.95–2.81 (m, 2H), 2.73 (s, 3H), 2.70–2.52 (m, 2H), 2.35 (s, 3H), 2.15 (s, 6H). ^13^C NMR (101 MHz, acetonitrile-*d*_3_) δ 149.8, 142.3, 141.4, 132.4, 131.5, 128.3, 127.7, 126.2, 125.4, 107.1, 56.4, 53.8, 41.5, 40.1, 39.6, 19.9, 13.7. HRMS: calcd. for C_20_H_26_N_2_Br [M + H]^+^ 373.1279; found: 373.1288.

#### (*R*)-2-Bromo-*N*-((4-(2,5-dimethyl-1*H*-pyrrol-1-yl)cyclopent-1-en-1-yl)methyl)-4-fluoro-*N*-methylaniline (**8i**)

Compound **8i** was synthesized according to general procedure 1. After flash chromatography (0–10% EtOAc in pentane), the product was isolated as an oil (19.0 mg, 0.050 mmol, 5% over two steps). [α]_D_^25^ = −16.0 (*c* = 0.1, THF). ^1^H NMR (400 MHz, acetonitrile-*d*_3_) δ 7.39 (dd, *J* = 8.4, 3.0 Hz, 1H), 7.23 (dd, *J* = 8.9, 5.6 Hz, 1H), 7.08 (ddd, *J* = 8.9, 8.1, 3.0 Hz, 1H), 5.65–5.62 (m, 1H), 5.61 (s, 2H), 5.04–4.94 (m, 1H), 3.74–3.67 (m, 1H), 3.61–3.55 (m, 1H), 2.91–2.79 (m, 2H), 2.66 (s, 3H), 2.57–2.47 (m, 2H), 2.08 (s, 6H). ^13^C NMR (101 MHz, acetonitrile-*d*_3_) δ 159.4 (d, *J* = 244.1 Hz), 148.5 (d, *J* = 2.8 Hz), 141.0, 128.3, 127.0, 124.5 (d, *J* = 8.8 Hz), 121.6 (d, *J* = 10.3 Hz), 121.2 (d, *J* = 25.3 Hz), 115.7 (d, *J* = 22.1 Hz), 107.1, 57.1, 53.5, 42.7, 41.3, 40.1, 13.5. HRMS: calcd. for C_19_H_23_N_2_BrF [M + H]^+^ 377.1029; found: 377.1031.

#### (*R*)-2,4-Dibromo-*N*-((4-(2,5-dimethyl-1*H*-pyrrol-1-yl)cyclopent-1-en-1-yl)methyl)-*N*-methylaniline (**8j**)

Compound **8j** was synthesized according to general procedure 1. After flash chromatography (0–10% EtOAc in pentane), the product was isolated as an oil (105 mg, 0.238 mmol, 29% over two steps). [α]_D_^25^ = −7.20 (*c* = 0.1, THF). ^1^H NMR (400 MHz, acetonitrile-*d*_3_) δ 7.74 (d, *J* = 2.3 Hz, 1H), 7.43 (dd, *J* = 8.7, 2.3 Hz, 1H), 7.11 (d, *J* = 8.7 Hz, 1H), 5.66–5.59 (m, 3H), 5.04–4.94 (m, 1H), 3.74 (d, *J* = 14.5 Hz, 1H), 3.62 (d, *J* = 14.5 Hz, 1H), 2.90–2.78 (m, 2H), 2.69 (s, 3H), 2.58–2.44 (m, 2H), 2.08 (s, 6H). ^13^C NMR (101 MHz, acetonitrile-*d*_3_) δ 151.4, 140.7, 136.6, 132.0, 128.3, 127.2, 124.9, 121.6, 116.3, 107.1, 56.7, 53.4, 42.1, 41.2, 40.1, 13.6. HRMS: calcd. for C_19_H_23_N_2_Br_2_ [M + H]^+^ 437.0228; found: 437.0233.

#### (*R*)-2,5-Dibromo-*N*-((4-(2,5-dimethyl-1*H*-pyrrol-1-yl)cyclopent-1-en-1-yl)methyl)-*N*-methylaniline (**8k**)

Compound **8k** was synthesized according to general procedure 1. After flash chromatography (0–10% EtOAc in pentane), the product was isolated as an oil (234 mg, 0.534 mmol, 66% over two steps). [α]_D_^25^ = −7.63 (*c* = 0.1, THF). ^1^H NMR (400 MHz, acetonitrile-*d*_3_) δ 7.45 (dd, *J* = 8.5, 1.0 Hz, 1H), 7.31 (d, *J* = 2.3 Hz, 1H), 7.07 (ddd, *J* = 8.5, 2.3, 1.0 Hz, 1H), 5.66–5.62 (m, 1H), 5.61 (s, 2H), 5.05–4.94 (m, 1H), 3.76 (d, *J* = 14.5 Hz, 1H), 3.64 (d, *J* = 14.5 Hz, 1H), 2.91–2.78 (m, 2H), 2.71 (s, 3H), 2.59–2.44 (m, 2H), 2.09 (s, 6H). ^13^C NMR (101 MHz, acetonitrile-*d*_3_) δ 153.3, 140.5, 136.0, 128.3, 128.1, 127.2, 126.5, 122.2, 119.4, 107.1, 56.5, 53.4, 42.1, 41.2, 40.1, 13.6. HRMS: calcd. for C_19_H_23_N_2_Br_2_ [M + H]^+^ 437.0228; found: 437.0209.

#### (*R*)-2-Bromo-*N*-((4-(2,5-dimethyl-1*H*-pyrrol-1-yl)cyclopent-1-en-1-yl)methyl)-5-methoxy-*N*-methylaniline (**8l**)

Compound **8l** was synthesized according to general procedure 1. After flash chromatography (0–10% EtOAc in pentane), the product was isolated as an oil (30.0 mg, 0.077 mmol, 9%). [α]_D_^25^ = −11.90 (*c* = 0.1, THF). ^1^H NMR (400 MHz, acetonitrile-*d*_3_) δ 7.43 (d, *J* = 8.7 Hz, 1H), 6.75 (d, *J* = 2.9 Hz, 1H), 6.54 (dd, *J* = 8.7, 2.9 Hz, 1H), 5.67–5.63 (m, 1H), 5.61 (s, 2H), 5.05–4.95 (m, 1H), 3.79–3.72 (m, 4H), 3.68–3.60 (d, *J* = 14.3 Hz, 1H), 2.92–2.79 (m, 2H), 2.69 (s, 3H), 2.60–2.46 (m, 2H), 2.09 (s, 6H). ^13^C NMR (101 MHz, acetonitrile-*d*_3_) δ 160.9, 152.9, 141.1, 134.8, 128.3, 126.8, 111.0, 110.6, 109.9, 107.0, 56.8, 56.2, 53.5, 42.2, 41.3, 40.1, 13.6. HRMS: calcd. for C_20_H_26_ON_2_Br [M + H]^+^ 389.1229; found: 389.1218.

#### (*R*)-3-Bromo-*N*-((4-(2,5-dimethyl-1*H*-pyrrol-1-yl)cyclopent-1-en-1-yl)methyl)-*N*-methylpyridin-2-amine (**8m**)

Compound **8m** was synthesized according to general procedure 1. After flash chromatography (0–10% EtOAc in pentane), the product was isolated as an oil (138 mg, 0.382 mmol, 47% over two steps). [α]_D_^25^ = −10.00 (*c* = 0.1, THF). ^1^H NMR (400 MHz, acetonitrile-*d*_3_) δ 8.17 (dd, *J* = 4.7, 1.7 Hz, 1H), 7.83 (dd, *J* = 7.7, 1.7 Hz, 1H), 6.78 (dd, *J* = 7.7, 4.7 Hz, 1H), 5.65–5.59 (m, 3H), 5.06–4.95 (m, 1H), 4.07–3.94 (m, 2H), 2.93–2.73 (m, 5H), 2.61–2.43 (m, 2H), 2.14 (s, 6H). ^13^C NMR (101 MHz, acetonitrile-*d*_3_) δ 160.7, 147.2, 143.5, 141.0, 128.3, 126.3, 119.0, 112.1, 107.1, 54.6, 53.6, 41.2, 40.1, 39.7, 13.7. HRMS: calcd. for C_18_H_23_N_3_Br [M + H]^+^ 360.1075; found: 360.1081.

#### (*S*)-2-Bromo-*N*-((4-(2,5-dimethyl-1*H*-pyrrol-1-yl)cyclopent-1-en-1-yl)methyl)-*N*-methylaniline (**8n**)

Compound **8n** was synthesized according to general procedure 1 on a larger scale (allyl chloride **5**, 680 mg, 3.24 mmol). After flash chromatography (0–4% EtOAc in pentane), the product was isolated as a white solid (1038 mg, 2.88 mmol, 89% over two steps). [α]_D_^25^ = 19.01 (*c* = 0.1, THF). ^1^H NMR (400 MHz, acetonitrile-*d*_3_) δ 7.56 (dd, *J* = 8.0, 1.5 Hz, 1H), 7.33–7.27 (m, 1H), 7.22 (dd, *J* = 8.1, 1.6 Hz, 1H), 6.97–6.91 (m, 1H), 5.66–5.62 (m, 1H), 5.61 (s, 2H), 5.05–4.95 (m, 1H), 3.75 (d, *J* = 14.0 Hz, 1H), 3.64 (d, *J* = 14.0 Hz, 1H), 2.92–2.80 (m, 2H), 2.70 (s, 3H), 2.58–2.48 (m, 2H), 2.09 (s, 6H). ^13^C NMR (101 MHz, Aacetonitrile-*d*_3_) δ 152.0, 141.1, 134.6, 129.3, 128.3, 126.8, 125.6, 123.5, 120.9, 107.1, 56.9, 53.5, 42.2, 41.3, 40.1, 13.6. HRMS: calcd. for C_19_H_24_BrN_2_ [M + H]^+^ 359.1123; found: 359.1130.

General procedure 2 for synthesis of *N*-methylspiroindolines **9a**–**9n**. To an oven-dried reaction vial, Pd(*t*-Bu_3_P)_2_ (0.1 equiv) and Et_3_N (2 equiv) were added. The vial was sealed and purged three times with nitrogen. DMF (1 mL) was added to the vial, which was stirred at room temperature for 5 min. A solution of *N*-methylallylaniline (1 equiv) in DMF (1 mL) was added, and the mixture heated at 80 °C for 16 h. Ethyl acetate (10 mL) was added, and the organic phase washed with water (3 × 10 mL), followed by brine (10 mL). The organic phase was dried with MgSO_4_ and evaporated. The product was isolated by automated flash chromatography.

#### (*R*)-2-Bromo-*N*-((4-(2,5-dimethyl-1*H*-pyrrol-1-yl)cyclopent-1-en-1-yl)methyl)-*N*-methylaniline (**9a**)

Compound **9a** was synthesized according to general procedure 2. After automated flash chromatography (0–12% EtOAc in pentane), the product was isolated as a white solid (67.0 mg, 0.241 mmol, 79%). [α]_D_^25^ = −324.1 (*c* = 0.1, THF). ^1^H NMR (400 MHz, Acetonitrile-*d*_3_) δ 7.09 (td, *J* = 7.7, 1.3 Hz, 1H), 7.00 (dd, *J* = 7.3, 1.0 Hz, 1H), 6.68 (td, *J* = 7.3, 1.0 Hz, 1H), 6.54 (d, *J* = 7.7 Hz, 1H), 5.96 (dd, *J* = 5.5, 2.0 Hz, 1H), 5.87 (dd, *J* = 5.5, 2.6 Hz, 1H), 5.65–5.57 (m, 3H), 3.45 (d, *J* = 9.1 Hz, 1H), 3.29 (d, *J* = 9.1 Hz, 1H), 2.73 (s, 3H), 2.66 (dd, *J* = 13.4, 8.5 Hz, 1H), 2.22 (s, 6H), 2.10 (dd, *J* = 13.4, 8.2 Hz, 1H). ^13^C NMR (101 MHz, acetonitrile-*d*_3_) δ 153.2, 138.0, 137.0, 132.9, 129.1, 128.7, 123.1, 119.2, 108.5, 107.3, 69.1, 61.5, 57.5, 46.7, 36.2, 14.2. HRMS: calcd. for C_19_H_23_N_2_ [M + H]^+^ 279.1861; found: 279.1860.

#### (1*R*,4*S*)-4-(2,5-Dimethyl-1*H*-pyrrol-1-yl)-1′-methylspiro[cyclopentane-1,3′-indolin]-2-ene-5′-carbonitrile (**9b**)

Compound **9b** was synthesized according to general procedure 2. After automated flash chromatography (0–12% EtOAc in pentane), the product was isolated as a white solid (70.4 mg, 0.232 mmol, 77%). [α]_D_^25^ = −294.9 (*c* = 1.0, THF). ^1^H NMR (400 MHz, acetonitrile-*d*_3_) δ 7.42 (dd, *J* = 8.2, 1.7 Hz, 1H), 7.25 (d, *J* = 1.7 Hz, 1H), 6.50 (d, *J* = 8.2 Hz, 1H), 5.99 (dd, *J* = 5.5, 2.0 Hz, 1H), 5.84 (dd, *J* = 5.5, 2.6 Hz, 1H), 5.67–5.59 (m, 3H), 3.68 (d, *J* = 9.7 Hz, 1H), 3.50 (d, *J* = 9.7 Hz, 1H), 2.84 (s, 3H), 2.71 (dd, *J* = 13.6, 8.5 Hz, 1H), 2.21 (s, 6H), 2.08 (dd, *J* = 13.6, 8.1 Hz, 1H). ^13^C NMR (101 MHz, acetonitrile-*d*_3_) δ 155.8, 137.8, 137.2, 134.9, 133.8, 128.8, 126.8, 121.5, 107.3, 107.1, 99.1, 68.0, 61.3, 57.0, 47.3, 34.3, 14.2. HRMS: calcd. for C_20_H_22_N_3_ [M + H]^+^ 304.1814; found: 304.1819.

#### (1*R*,4*S*)-5′-Chloro-4-(2,5-dimethyl-1*H*-pyrrol-1-yl)-1′-methylspiro[cyclopentane-1,3′-indolin]-2-ene (**9c**)

Compound **9c** was synthesized according to general procedure 2. After automated flash chromatography (0–12% EtOAc in pentane), the product was isolated as an oil (38.0 mg, 0.121 mmol, 61%). [α]_D_^25^ = −420.24 (*c* = 0.1, THF). ^1^H NMR (400 MHz, acetonitrile-*d*_3_) δ 7.06 (dd, *J* = 8.3, 2.2 Hz, 1H), 6.98 (d, *J* = 2.2 Hz, 1H), 6.48 (d, *J* = 8.3 Hz, 1H), 5.98 (dd, *J* = 5.5, 2.0 Hz, 1H), 5.85 (dd, *J* = 5.5, 2.6 Hz, 1H), 5.65–5.56 (m, 3H), 3.49 (d, *J* = 9.2 Hz, 1H), 3.33 (d, *J* = 9.2 Hz, 1H), 2.73 (s, 3H), 2.68 (dd, *J* = 13.6, 8.6 Hz, 1H), 2.21 (s, 6H), 2.09 (dd, *J* = 13.6, 8.1 Hz, 1H). ^13^C NMR (101 MHz, acetonitrile-*d*_3_) δ 152.0, 139.3, 137.3, 133.6, 128.8, 128.7, 123.4, 122.9, 109.3, 107.3, 69.1, 61.4, 57.5, 46.5, 36.0, 14.2. HRMS: calcd. for C_19_H_22_N_2_Cl [M + H]^+^ 313.1472; found: 313.1467.

#### (1*R*,4*S*)-4-(2,5-Dimethyl-1*H*-pyrrol-1-yl)-1′-methyl-4′-(trifluoromethyl)spiro[cyclopentane-1,3′-indolin]-2-ene (**9d**)

Compound **9d** was synthesized according to general procedure 2. After flash chromatography (0–12% EtOAc in pentane), the product was isolated as an oil (19.0 mg, 0.055 mmol, 59%). [α]_D_^25^ = −462.16 (*c* = 0.1, THF). ^1^H NMR (400 MHz, acetonitrile-*d*_3_) δ 7.12 (d, *J* = 7.6 Hz, 1H), 6.96 (d, *J* = 7.6 Hz, 1H), 6.74 (s, 1H), 6.02 (dd, *J* = 5.5, 2.0 Hz, 1H), 5.88 (dd, *J* = 5.5, 2.6 Hz, 1H), 5.66–5.58 (m, 3H), 3.57 (d, *J* = 9.3 Hz, 1H), 3.41 (d, *J* = 9.3 Hz, 1H), 2.79 (s, 3H), 2.70 (dd, *J* = 13.6, 8.5 Hz, 1H), 2.21 (s, 6H), 2.13–2.08 (m, 1H). ^13^C NMR (101 MHz, acetonitrile-*d*_3_) δ 153.5, 141.5, 137.2, 133.8, 130.9 (q, *J* = 31.2 Hz), 128.8, 125.6 (q, *J* = 271.1 Hz, determined through HMBC), 123.5, 115.6 (q, *J* = 4.0 Hz), 107.3, 104.1 (q, *J* = 3.8 Hz), 68.7, 61.4, 57.5, 46.8, 35.5, 14.1. HRMS: calcd. for C_20_H_22_N_2_F_3_ [M + H]^+^ 347.1735; found: 347.1723.

#### (1*R*,4*S*)-4-(2,5-Dimethyl-1*H*-pyrrol-1-yl)-1′,4′-dimethylspiro[cyclopentane-1,3′-indolin]-2-ene (**9e**)

Compound **9e** was synthesized according to general procedure 2. After flash chromatography (0–6% EtOAc in pentane), the product was isolated as a white solid (54.9 mg, 0.188 mmol, 66%). [α]_D_^25^ = −406.14 (*c* = 0.1, THF). ^1^H NMR (400 MHz, acetonitrile-*d*_3_) δ 6.99 (dd, *J* = 7.8, 7.6 Hz, 1H), 6.48 (dt, *J* = 7.6, 0.8 Hz, 1H), 6.40 (d, *J* = 7.8 Hz, 1H), 5.91 (dd, *J* = 5.5, 2.1 Hz, 1H), 5.81 (dd, *J* = 5.5, 2.6 Hz, 1H), 5.65 (s, 2H), 5.60–5.52 (m, 1H), 3.46 (d, *J* = 9.0 Hz, 1H), 3.22 (d, *J* = 9.0 Hz, 1H), 2.83 (dd, *J* = 14.4, 9.4 Hz, 1H), 2.70 (s, 3H), 2.23 (s, 6H), 2.22 (s, 3H), 1.94 (dd, *J* = 14.4, 7.0 Hz, 1H). ^13^C NMR (101 MHz, acetonitrile-*d*_3_) δ 154.0, 137.2, 134.9, 133.1, 132.1, 129.1, 128.8, 121.9, 107.2, 106.6, 70.3, 62.5, 57.8, 44.3, 36.2, 17.6, 14.2. HRMS: calcd. for C_20_H_25_N_2_ [M + H]^+^ 293.2018; found: 293.2013.

#### (1*R*,4*S*)-4-(2,5-Dimethyl-1*H*-pyrrol-1-yl)-1′,5′-dimethylspiro[cyclopentane-1,3′-indolin]-2-ene (**9f**)

Compound **9f** was synthesized according to general procedure 2. After flash chromatography (0–12% EtOAc in pentane) the product was isolated as a white solid (40.9 mg, 0.140 mmol, 59%). [α]_D_^25^ = −381.23 (*c* = 0.1, THF). ^1^H NMR (400 MHz, acetonitrile-*d*_3_) δ 6.93–6.88 (m, 1H), 6.85 (dq, *J* = 1.8, 0.6 Hz, 1H), 6.45 (d, *J* = 7.9 Hz, 1H), 5.95 (dd, *J* = 5.5, 2.0 Hz, 1H), 5.85 (dd, *J* = 5.5, 2.6 Hz, 1H), 5.66–5.56 (m, 3H), 3.41 (d, *J* = 9.0 Hz, 1H), 3.24 (d, *J* = 9.0 Hz, 1H), 2.69 (s, 3H), 2.64 (dd, *J* = 13.4, 8.5 Hz, 1H), 2.24–2.21 (m, 9H), 2.09 (dd, *J* = 13.4, 8.1 Hz, 1H). ^13^C NMR (101 MHz, acetonitrile-*d*_3_) δ 151.2, 138.1, 137.3, 132.8, 129.3, 128.8, 128.5, 123.9, 108.6, 107.2, 69.4, 61.5, 57.6, 46.5, 36.7, 20.8, 14.2. HRMS: calcd. for C_20_H_25_N_2_ [M + H]^+^ 293.2018; found: 293.2010.

#### (1*R*,4*S*)-4-(2,5-Dimethyl-1*H*-pyrrol-1-yl)-1′,6′-dimethylspiro[cyclopentane-1,3′-indolin]-2-ene (**9g**)

Compound **9g** was synthesized according to general procedure 2. After flash chromatography (0–12% EtOAc in pentane), the product was isolated as a white solid (69.0 mg, 0.236 mmol, 65%). [α]_D_^25^ = −449.31 (*c* = 0.1, THF). ^1^H NMR (400 MHz, acetonitrile-*d*_3_) δ 6.88 (d, *J* = 7.4 Hz, 1H), 6.51 (dq, *J* = 7.4, 0.7 Hz, 1H), 6.40–6.37 (m, 1H), 5.94 (dd, *J* = 5.5, 2.0 Hz, 1H), 5.85 (dd, *J* = 5.5, 2.6 Hz, 1H), 5.64 (s, 2H), 5.62–5.55 (m, 1H), 3.44 (d, *J* = 9.1 Hz, 1H), 3.27 (d, *J* = 9.1 Hz, 1H), 2.72 (s, 3H), 2.63 (dd, *J* = 13.4, 8.5 Hz, 1H), 2.27 (s, 3H), 2.22 (s, 6H), 2.09 (dd, *J* = 13.4, 8.2 Hz, 1H). ^13^C NMR (101 MHz, acetonitrile-*d*_3_) δ 153.4, 138.9, 138.2, 134.3, 132.7, 128.7, 122.9, 119.8, 109.4, 107.2, 69.3, 61.5, 57.2, 46.7, 36.2, 21.8, 14.2. HRMS: calcd. for C_20_H_25_N_2_ [M + H]^+^ 293.2018; found: 293.2023.

#### (1*R*,4*S*)-4-(2,5-Dimethyl-1*H*-pyrrol-1-yl)-1′,7′-dimethylspiro[cyclopentane-1,3′-indolin]-2-ene (**9h**)

Compound **9h** was synthesized according to general procedure 2. After flash chromatography (0–6% EtOAc in pentane), the product was isolated as a white solid (69.0 mg, 0.236 mmol, 80%). [α]_D_^25^ = −433.33 (c = 0.1, THF). ^1^H NMR (400 MHz, acetonitrile-*d*_3_) δ 6.89–6.84 (m, 2H), 6.66 (t, *J* = 7.4 Hz, 1H), 5.94 (dd, *J* = 5.5, 2.0 Hz, 1H), 5.84 (dd, *J* = 5.5, 2.6 Hz, 1H), 5.63 (s, 2H), 5.60–5.54 (m, 1H), 3.45 (d, *J* = 9.8 Hz, 1H), 3.29 (d, *J* = 9.8 Hz, 1H), 2.92 (s, 3H), 2.61 (dd, *J* = 13.4, 8.5 Hz, 1H), 2.35 (s, 3H), 2.21 (s, 6H), 2.08 (dd, *J* = 13.4, 8.1 Hz, 1H). ^13^C NMR (101 MHz, Acetonitrile-*d*_3_) δ 151.1, 138.5, 138.1, 132.7, 132.2, 128.8, 121.5, 121.2, 120.6, 107.2, 70.5, 61.5, 57.6, 47.3, 40.2, 19.6, 14.2. HRMS: calcd. for C_20_H_25_N_2_ [M + H]^+^ 293.2018; found: 293.2016.

#### (1*R*,4*S*)-4-(2,5-Dimethyl-1*H*-pyrrol-1-yl)-5′-fluoro-1′-methylspiro[cyclopentane-1,3′-indolin]-2-ene (**9i**)

Compound **9i** was synthesized according to general procedure 2. After flash chromatography (0–7% EtOAc in pentane), the product was isolated as an oil (8.00 mg, 0.027 mmol, 63%). [α]_D_^25^ = −334.79 (*c* = 0.1, THF). ^1^H NMR (400 MHz, acetonitrile-*d*_3_) δ 6.87–6.77 (m, 2H), 6.48 (dd, *J* = 8.4, 4.2 Hz, 1H), 5.99 (dd, *J* = 5.5, 2.0 Hz, 1H), 5.86 (dd, *J* = 5.5, 2.6 Hz, 1H), 5.66–5.56 (m, 3H), 3.45 (d, *J* = 9.1 Hz, 1H), 3.30 (d, *J* = 9.1 Hz, 1H), 2.70 (s, 3H), 2.67 (dd, *J* = 13.6, 8.6 Hz, 1H), 2.21 (s, 6H), 2.10 (dd, *J* = 13.6, 8.1 Hz, 1H). ^13^C NMR (101 MHz, acetonitrile-*d*_3_) δ 157.7 (d, *J* = 232.9 Hz), 149.7, 139.0 (d, *J* = 7.2 Hz), 137.3, 133.6, 128.8, 114.7 (d, *J* = 23.1 Hz), 110.7 (d, *J* = 23.9 Hz), 108.9 (d, *J* = 8.0 Hz), 107.3, 69.6, 61.4, 57.6, 46.3, 36.8, 14.2. HRMS: calcd. for C_19_H_22_N_2_F [M + H]^+^ 297.1767; found: 297.1767.

#### (1*R*,4*S*)-5′-Bromo-4-(2,5-dimethyl-1*H*-pyrrol-1-yl)-1′-methylspiro[cyclopentane-1,3′-indolin]-2-ene (**9j**)

Compound **9j** was synthesized according to general procedure 2. After flash chromatography (0–12% EtOAc in pentane), the product was isolated as a yellowish oil (57.1 mg, 0.160 mmol, 67%). [α]_D_^25^ = −539.5 (*c* = 0.1, THF). ^1^H NMR (400 MHz, acetonitrile-*d*_3_) δ 7.20 (dd, *J* = 8.3, 2.1 Hz, 1H), 7.10 (d, *J* = 2.1 Hz, 1H), 6.44 (d, *J* = 8.3 Hz, 1H), 5.97 (dd, *J* = 5.5, 2.0 Hz, 1H), 5.85 (dd, *J* = 5.5, 2.6 Hz, 1H), 5.65–5.56 (m, 3H), 3.49 (d, *J* = 9.2 Hz, 1H), 3.33 (d, *J* = 9.2 Hz, 1H), 2.72 (s, 3H), 2.68 (dd, *J* = 13.7, 8.6 Hz, 1H), 2.21 (s, 6H), 2.08 (dd, *J* = 13.7, 8.0 Hz, 1H). ^13^C NMR (101 MHz, acetonitrile-*d*_3_) δ 152.4, 139.7, 137.3, 133.6, 131.6, 128.8, 126.1, 109.9, 109.9, 107.2, 69.0, 61.4, 57.5, 46.5, 35.9, 14.2. HRMS: calcd. for C_19_H_22_N_2_Br [M + H]^+^ 357.0966; found: 357.0966.

#### (1*R*,4*S*)-6′-Bromo-4-(2,5-dimethyl-1*H*-pyrrol-1-yl)-1′-methylspiro[cyclopentane-1,3′-indolin]-2-ene (**9k**)

Compound **9k** was synthesized according to general procedure 2. After flash chromatography (0–12% EtOAc in pentane), the product was isolated as a yellowish oil (117 mg, 0.326 mmol, 65%). [α]_D_^25^ = −397.66 (*c* = 0.1, THF). ^1^H NMR (400 MHz, acetonitrile-*d*_3_) δ 6.88 (d, *J* = 7.8 Hz, 1H), 6.78 (dd, *J* = 7.8, 1.8 Hz, 1H), 6.65 (d, *J* = 1.8 Hz, 1H), 5.97 (dd, *J* = 5.5, 2.0 Hz, 1H), 5.84 (dd, *J* = 5.5, 2.6 Hz, 1H), 5.63 (s, 2H), 5.61–5.54 (m, 1H), 3.52 (d, *J* = 9.2 Hz, 1H), 3.35 (d, *J* = 9.2 Hz, 1H), 2.73 (s, 3H), 2.66 (dd, *J* = 13.5, 8.5 Hz, 1H), 2.20 (s, 6H), 2.08 (dd, *J* = 13.5, 8.2 Hz, 1H). ^13^C NMR (101 MHz, Acetonitrile-*d*_3_) δ 154.6, 137.5, 136.5, 133.4, 128.8, 124.6, 122.3, 121.2, 111.0, 107.3, 68.8, 61.4, 57.2, 46.7, 35.6, 14.2. HRMS: calcd. for C_19_H_22_N_2_Br [M + H]^+^ 357.0966; found: 357.0959.

#### (1*R*,4*S*)-4-(2,5-Dimethyl-1*H*-pyrrol-1-yl)-6′-methoxy-1′-methylspiro[cyclopentane-1,3′-indolin]-2-ene (**9l**)

Compound **9l** was synthesized according to general procedure 2. After flash chromatography (0–6% EtOAc in pentane), the product was isolated as an oil (9.1 mg, 0.030 mmol, 61%). [α]_D_^25^ = −395.23 (*c* = 0.1, THF). ^1^H NMR (400 MHz, acetonitrile-*d*_3_) δ 6.87 (d, *J* = 8.0 Hz, 1H), 6.21 (dd, *J* = 8.0, 2.3 Hz, 1H), 6.12 (d, *J* = 2.3 Hz, 1H), 5.92 (dd, *J* = 5.5, 2.0 Hz, 1H), 5.84 (dd, *J* = 5.5, 2.6 Hz, 1H), 5.63 (s, 2H), 5.61–5.54 (m, 1H), 3.74 (s, 3H), 3.47 (d, *J* = 9.1 Hz, 1H), 3.30 (d, *J* = 9.1 Hz, 1H), 2.72 (s, 3H), 2.62 (dd, *J* = 13.4, 8.5 Hz, 1H), 2.21 (s, 6H), 2.07 (dd, *J* = 13.4, 8.1 Hz, 1H). ^13^C NMR (101 MHz, acetonitrile-*d*_3_) δ 161.9, 154.5, 138.3, 132.5, 129.4, 128.8, 123.5, 107.2, 103.5, 95.6, 69.5, 61.5, 56.9, 55.9, 46.8, 36.0, 14.2. HRMS: calcd. for C_20_H_25_N_2_O [M + H]^+^ 309.1967; found: 309.1959.

#### (1*R*,4*S*)-4-(2,5-Dimethyl-1*H*-pyrrol-1-yl)-1′-methyl-1′,2′-dihydrospiro[cyclopentane-1,3′-pyrrolo[2,3-*b*]pyridin]-2-ene (**9m**)

Compound **9m** was synthesized according to general procedure 2. After flash chromatography (0–20% EtOAc in pentane), the product was isolated as a white solid (85.9 mg, 0.307 mmol, 81%). [α]_D_^25^ = −243.08 (*c* = 0.1, THF). ^1^H NMR (400 MHz, acetonitrile-*d*_3_) δ 7.86 (dd, *J* = 5.3, 1.6 Hz, 1H), 7.20 (dd, *J* = 7.1, 1.6 Hz, 1H), 6.49 (dd, *J* = 7.1, 5.3 Hz, 1H), 5.99 (dd, *J* = 5.5, 2.0 Hz, 1H), 5.86 (dd, *J* = 5.5, 2.6 Hz, 1H), 5.64 (s, 2H), 5.62–5.56 (m, 1H), 3.60 (d, *J* = 9.4 Hz, 1H), 3.42 (d, *J* = 9.4 Hz, 1H), 2.88 (s, 3H), 2.71 (dd, *J* = 13.5, 8.4 Hz, 1H), 2.21 (s, 6H), 2.08 (dd, *J* = 13.5, 8.1 Hz, 1H). ^13^C NMR (101 MHz, acetonitrile-*d*_3_) δ 163.3, 147.8, 137.5, 133.4, 130.1, 130.0, 128.8, 113.7, 107.3, 65.7, 61.4, 55.2, 47.4, 32.8, 14.2. HRMS: calcd. for C_18_H_22_N_3_ [M + H]^+^ 280.1814; found: 280.1815.

#### (1*S*,4*R*)-4-(2,5-Dimethyl-1*H*-pyrrol-1-yl)-1′-methylspiro[cyclopentane-1,3′-indolin]-2-ene (**9n**)

Compound **9n** was synthesized according to general procedure 2. After flash chromatography (0–5% EtOAc in pentane), the product was isolated as a white solid (68.0 mg, 0.243 mmol, 73%). [α]_D_^25^ = 315.1 (*c* = 0.1, THF). ^1^H NMR (400 MHz, acetonitrile-*d*_3_) δ 7.09 (td, *J* = 7.8, 1.4 Hz, 1H), 7.01 (dd, *J* = 7.4, 1.4 Hz, 1H), 6.68 (ddd, *J* = 7.4, 0.9 Hz, 1H), 6.54 (d, *J* = 7.8 Hz, 1H), 5.96 (dd, *J* = 5.5, 2.0 Hz, 1H), 5.87 (dd, *J* = 5.5, 2.6 Hz, 1H), 5.64 (s, 2H), 5.63–5.56 (m, 1H), 3.46 (d, *J* = 9.1 Hz, 1H), 3.29 (d, *J* = 9.1 Hz, 1H), 2.74 (s, 3H), 2.66 (dd, *J* = 13.5, 8.5 Hz, 1H), 2.22 (s, 6H), 2.11 (dd, *J* = 13.5, 8.2 Hz, 1H). ^13^C NMR (101 MHz, acetonitrile-*d*_3_) δ 153.2, 138.0, 137.0, 132.9, 129.1, 128.8, 123.1, 119.1, 108.5, 107.2, 69.0, 61.5, 57.5, 46.7, 36.2, 14.2. HRMS: calcd. for C_19_H_23_N_2_ [M + H]^+^ 279.1861; found: 279.1861.

#### Benzyl-(1*R*,4*S*)-4-(2,5-dimethyl-1*H*-pyrrol-1-yl)-1′-methylspiro[cyclopentane-1,3′-indolin]-2-ene-6′-carboxylate (**10**)

To the first tube of a 5 mL H-tube equipped with Teflon-coated stirring bars were added **9k** (100 mg, 0.280 mmol), palladium acetate (1.3 mg, 5.6 μmol), XantPhos (6.5 mg, 11 μmol), and Et_3_N (78 μL, 0.56 mmol). To the other was added Mo(CO)_6_ (148 mg, 0.560 mmol). The H-tube was sealed and purged three times with nitrogen gas. To each of the chambers was added benzyl alcohol (1 mL). DBU (125 μL, 0.840 mmol) was added to the Mo(CO)_6_-containing chamber, and the reaction was stirred at 120 °C for 2 h. Ethyl acetate (10 mL) was added, and the organic phase was washed with water (2 × 10 mL), followed by brine (10 mL). The organic phase was evaporated, and the crude product was purified by automatic flash chromatography (0–10% EtOAc in pentane) to provide the product as an oil (76 mg, 0.18 mmol, 64%). [α]_D_^25^ = −285.10 (*c* = 0.1, THF). ^1^H NMR (400 MHz, acetonitrile-*d*_3_) δ 7.48–7.43 (m, 1H), 7.43–7.32 (m, 5H), 7.11 (d, *J* = 1.5 Hz, 1H), 7.08 (dd, *J* = 7.6, 0.5 Hz, 1H), 6.01 (dd, *J* = 5.5, 2.0 Hz, 1H), 5.87 (dd, *J* = 5.5, 2.6 Hz, 1H), 5.64 (s, 2H), 5.62–5.56 (m, 1H), 5.32 (s, 2H), 3.54 (d, *J* = 9.2 Hz, 1H), 3.37 (d, *J* = 9.2 Hz, 1H), 2.78 (s, 3H), 2.70 (dd, *J* = 13.6, 7.5 Hz, 1H), 2.21 (s, 6H), 2.10 (dd, *J* = 13.6, 8.1 Hz, 1H). ^13^C NMR (101 MHz, acetonitrile-*d*_3_) δ 167.4, 153.4, 142.8, 137.8, 137.3, 133.7, 131.3, 129.6, 129.1, 128.9, 128.8, 123.1, 121.1, 108.3, 107.3, 68.8, 67.2, 61.4, 57.6, 46.6, 35.8, 14.2. HRMS: calcd. for C_27_H_29_N_2_O_2_ [M + H]^+^ 413.2229; found: 413.2233.

#### Benzyl (1*R*,4*S*)-4-Amino-1′-methylspiro[cyclopentane-1,3′-indolin]-2-ene-6′-carboxylate (**11**)

A reaction vial was loaded with hydroxylamine hydrochloride (84.2 mg, 1.21 mmol) and **10** (50.0 mg, 0.121 mmol). Next, EtOH:H_2_O (2:1, 1 mL) was added, and the mixture was heated to 100 °C and stirred at that temperature for 16 h. The crude product was isolated through automated reverse phase flash chromatography (5–100% MeCN in water (0.05% formic acid)) to provide the product as a white solid (21.7 mg, 0.0649 mmol, 54%). [α]_D_^25^ = −202.0 (*c* = 0.1, THF). ^1^H NMR (500 MHz, methanol-*d*_4_) δ 8.56 (bs, 2H), 7.47–7.41 (m, 3H), 7.41–7.36 (m, 2H), 7.36–7.30 (m, 1H), 7.16 (s, 1H), 6.98 (d, *J* = 7.6 Hz, 1H), 6.08 (dd, *J* = 5.6, 1.6 Hz, 1H), 5.92 (dd, *J* = 5.6, 1.9 Hz, 1H), 5.33 (s, 2H), 4.57–4.50 (m, 1H), 3.47 (d, *J* = 9.3 Hz, 1H), 3.36 (d, *J* = 9.3 Hz, 1H), 2.81 (s, 3H), 2.65 (dd, *J* = 14.2, 8.1 Hz, 1H), 2.07 (dd, *J* = 14.2, 5.6 Hz, 1H). ^13^C NMR (126 MHz, methanol-*d*_4_) δ 168.2, 153.8, 142.7, 142.0, 137.7, 131.8, 129.6, 129.3, 129.2, 123.2, 121.7, 109.0, 69.0, 67.7, 58.6, 57.5, 44.3, 35.8. HRMS: calcd. for C_21_H_23_N_2_O_2_ [M + H]^+^ 335.1760; found: 335.1766.

#### (1*R*,4*S*)-4-(2,5-Dimethyl-1*H*-pyrrol-1-yl)-1′-methylspiro[cyclopentane-1,3′-indolin]-2-ene-6′-carboxylic acid (**12**)

A reaction vial was loaded with **10** (68.3 mg, 0.166 mmol) and lithium hydroxide (20.0 mg, 0.835 mmol), and a THF:H_2_O mixture (3:1, 1 mL) was added. The mixture was heated to 50 °C and stirred at that temperature for 20 h. The reaction mixture was diluted with 10 mL water and washed with EtOAc (3 × 10 mL). The water phase was acidified with 2 M HCl and extracted with EtOAc (3 × 10 mL). The organic phase was dried with MgSO_4_, filtered, and evaporated to yield the product as a dark brown oil (44.4 mg, 0.138 mmol), 83%). The sample was too dark to obtain value for optical rotation. ^1^H NMR (400 MHz, chloroform-*d*) δ 7.57 (dd, *J* = 7.6, 1.4 Hz, 1H), 7.25 (d, *J* = 1.4 Hz, 1H), 7.04 (d, *J* = 7.6 Hz, 1H), 6.07 (dd, *J* = 5.5, 2.0 Hz, 1H), 5.93 (dd, *J* = 5.5, 2.5 Hz, 1H), 5.78 (s, 2H), 5.62–5.54 (m, 1H), 3.60 (d, *J* = 9.1 Hz, 1H), 3.39 (d, *J* = 9.1 Hz, 1H), 2.87 (s, 3H), 2.78 (dd, *J* = 13.8, 8.5 Hz, 1H), 2.28 (s, 6H), 2.25 (dd, *J* = 13.8, 8.1 Hz, 1H). ^13^C NMR (101 MHz, chloroform-*d*) δ 172.1, 151.6, 142.4, 136.5, 132.8, 129.6, 128.2, 122.0, 121.8, 108.9, 106.4, 68.3, 60.6, 56.9, 46.1, 35.9, 14.1. HRMS: calcd. for C_20_H_23_N_2_O_2_ [M + H]^+^ 323.1760; found: 323.1759.

## Supporting Information Available

Copies of all ^1^H NMR, ^19^F and ^13^C NMR spectra, NMR 2D-spectra, structural information, computational and X-ray crystallography data detailed in the experimental section (PDF)
Crystallographic information file for compound 9m(CIF)
PDB file for X-ray crystal structure of compound 9m (PDB)
ORTEP representation of X-ray crystal structure of compound 9m (PDF)
X-ray crystallography data for compound 9m (PDF)

## Supplementary Material

ao2c04111_si_001.pdf

ao2c04111_si_002.cif

ao2c04111_si_003.pdb
