# Supplementary material for: Diastereoselective Synthesis of N-Methylspiroindolines by Intramolecular Mizoroki–Heck Annulations
Source: ACS Omega. 2022 Aug 26;7(36):32525–35. doi: 10.1021/acsomega.2c04111 (PMC9476516; doi:10.1021/acsomega.2c04111)
Supplement: Supplementary file 1 — ao2c04111_si_001.pdf [file ao2c04111_si_001.pdf]

# Diastereoselective Synthesis of *N*-Methylspiroindolines by Intramolecular Mizoroki-Heck Annulations

## *Supporting Information*

*Jens Lindman, Greeshma Gopalan, Carlos Palo-Nieto, Peter Brandt*[ǂ], *Johan Gising, Mats Larhed*[*]

Mats.larhed@ilk.uu.se

Uppsala University, Department of Medicinal Chemistry, Husargatan 3, SE-751 23 Uppsala, Sweden

ǂ Beactica AB, Virdings allé 2, SE-754 50 Uppsala, Sweden

## Table of Contents

**Spiroindoline-containing bioactive compounds** ........ **S2**

**Reported synthetic methods for the synthesis of spiroindolines** ........ **S3**

**Computational details** ........ **S4**

**NOESY** ........ **S10**

**X-ray crystallography data** ........ **S11**

**NMR-spectra of compounds 5 - 12** ........ **S12**

**Cartesian coordinates for structures S2 – S11** ........ **S51**

**References** ........ **S72**

# Spiroindoline-containing bioactive compounds

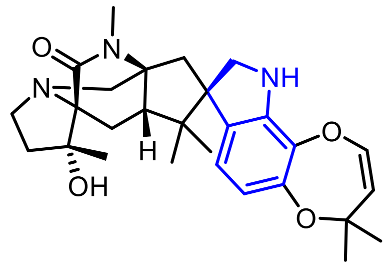

Derquantel
antihelminthicum

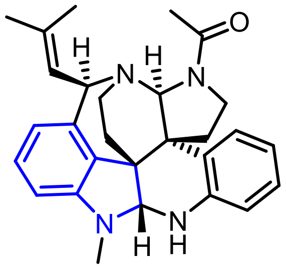

Communesin F
cytotoxic alkaloid

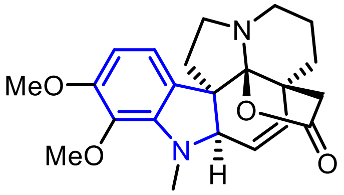

Aspidophytine
insecticidal alkaloid

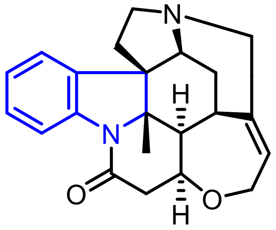

Strychnine
alkaloid neurotoxin

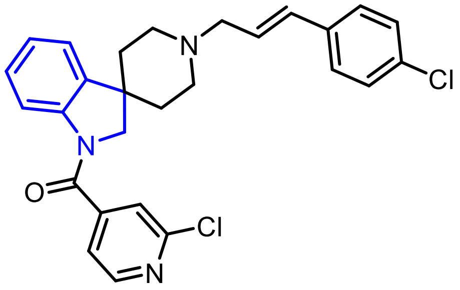

SYN876
VAChT ligand

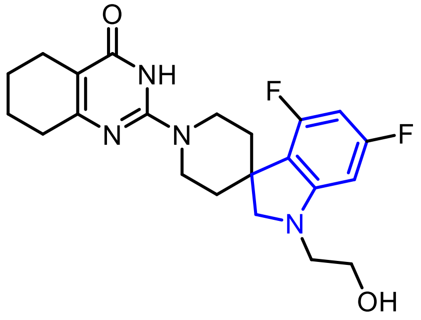

RK-287107
TNKS/TNKS2 inhibitor

**Figure S1.** Bioactive compounds containing a spiroindoline motif. [1,2] The spiroindoline structure is highlighted in blue.

# Reported synthetic methods for the synthesis of spiroindolines

*Catalytic asymmetric dearomatization by You et al.*[3]

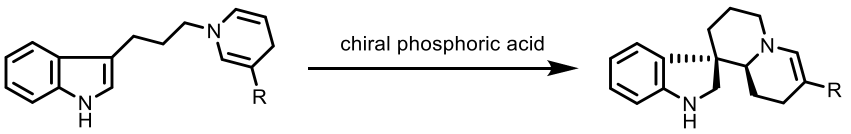


*Enantioselective dearomative spirocyclization by Xia et al.*[4]

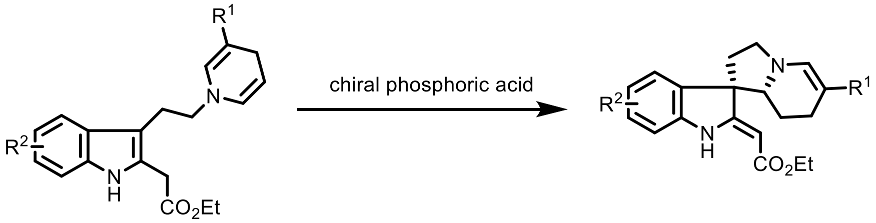


*Photocatalytic dearomative spirocyclization by You et al.*[5]

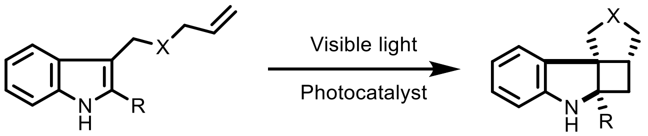


*Silver-catalyzed dearomative spirocyclization by Pereshivko, Peshkov et al.*[6]

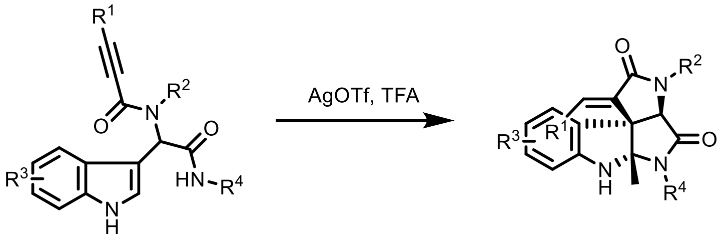


*Silver triflate/N-fluorobenzenesulfonamide-catalyzed cycloisomerization by Liu et al.*[7]

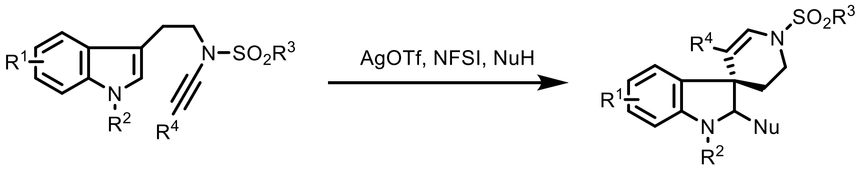


*Palladium-catalyzed dearomatization by You et al.*[8]

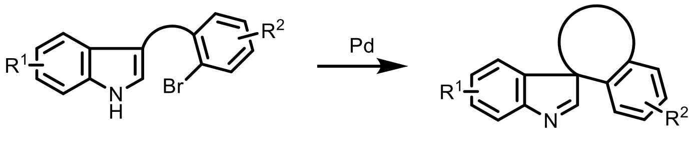

## Computational details

Density functional theory calculations were performed essentially as described previously for similar systems.[9,10] This implies the use of the B3LYP-D3 a posteriori-corrected functional[11] together with the LACVP** basis set[12] for geometry optimizations, solvation effects, zero point energies and contributions to free energies at 80°C. Final energies were calculated using B3LYP-D3/LACV3P+**. Solvation effects were estimated by use of PBF; a Poisson-Boltzmann continuum solvation model.[13] The density functional calculations were performed in Jaguar version 12.9.123, release 2021-3, Schrodinger, Inc., New York, NY, 2016.[14] The results from the DFT calculations are displayed in Table S1 and Figure S2 – S11.

**Table S1. Relative energies in kcal/mol for Heck reaction transition states leading to the *anti* and *syn* product.**

| Entry | Gas phase energy LACVP** | Total free energy at 298.15 K | Free energy contribution | Gas phase energy LACV3P+** | Solution phase energy | Solvation effect | $\Delta G_{sol}$ |
|---|---|---|---|---|---|---|---|
| **π complex intermediate (R) *Anti*** | 0.0 | 0.0 | 0.0 | 0.0 | 0.0 | 0.0 | 0.0 |
| **Migratory insertion transition state (MI) *Anti*** | 11.1 | 10.0 | -1.1 | 11.2 | 10.7 | -0.5 | 9.6 |
| **σ complex intermediate (I) *Anti*** | -12.1 | -12.6 | -0.4 | -11.2 | -11.3 | -0.1 | -11.7 |
| **β-hydride elimination transition state (BHE) *Anti*** | -1.8 | -5.5 | -3.7 | -3.4 | -5.3 | -1.9 | -9.0 |
| **Product (P) *Anti*** | -8.6 | -11.2 | -2.6 | -9.8 | -13.9 | -4.0 | -16.5 |
| **π complex intermediate (R) *Syn*** | 0.8 | 0.4 | -0.5 | 0.7 | -0.5 | -1.2 | -1.0 |
| **Migratory insertion transition state (MI) *Syn*** | 15.3 | 13.9 | -1.4 | 15.2 | 13.9 | -1.3 | 12.5 |
| **σ complex intermediate (I) *Syn*** | -11.7 | -12.1 | -0.4 | -10.7 | -10.8 | -0.1 | -11.2 |
| **β-hydride elimination transition state (BHE) *Syn*** | -4.0 | -6.6 | -2.6 | -4.7 | -7.4 | -2.7 | -10.0 |
| **Product (P) *Syn*** | -13.0 | -14.3 | -1.4 | -13.5 | -15.8 | -2.3 | -17.2 |

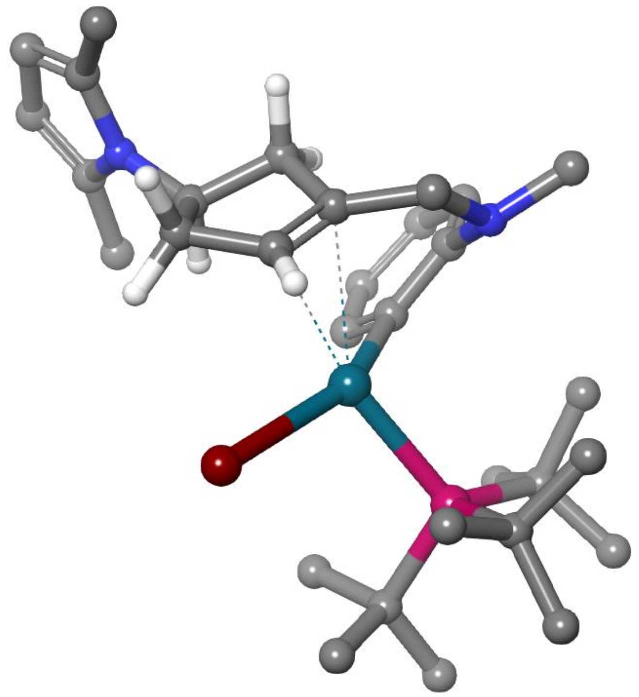

**Figure S2.** π complex intermediate (R) leading to *anti* product.

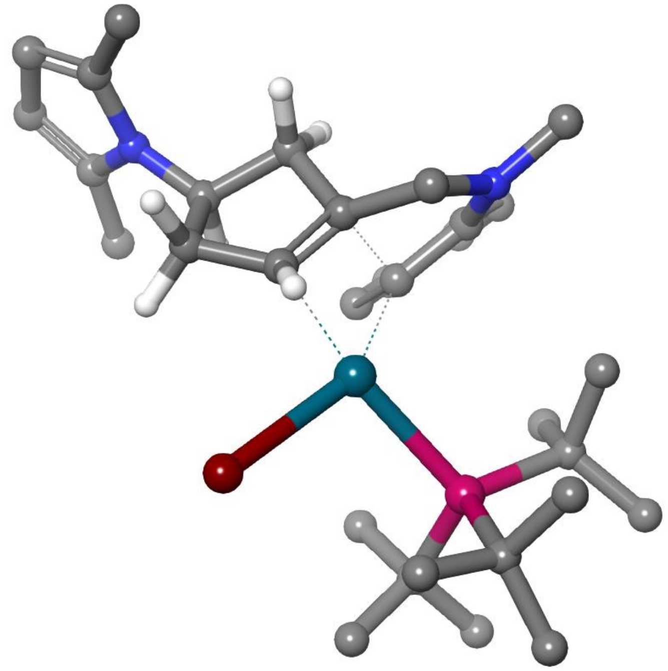

**Figure S3.** Migratory insertion transition step (MI) leading to *anti* product.

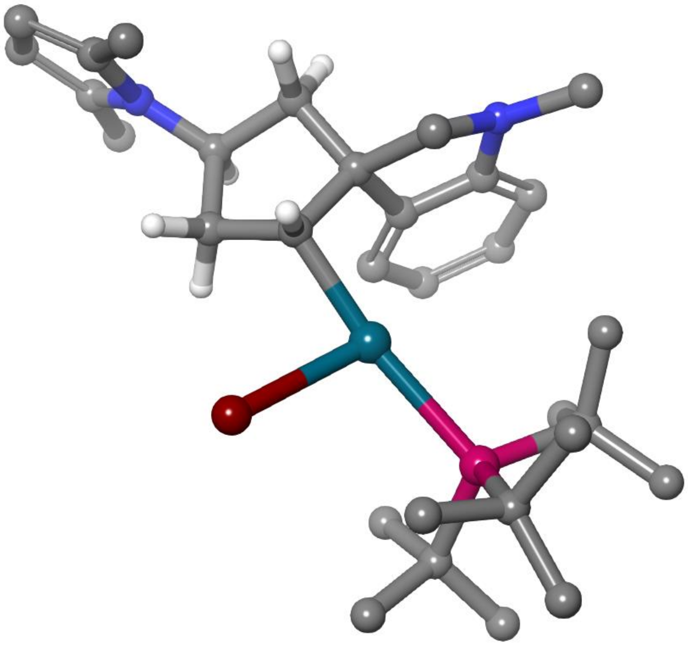

**Figure S4.** σ complex intermediate (I) leading to *anti* product.

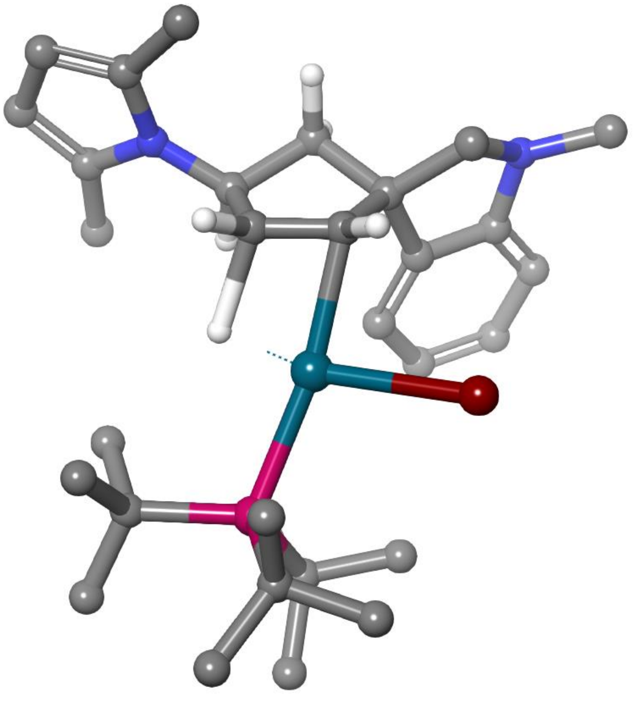

**Figure S5.** β-hydride elimination transition step (BHE) leading to *anti* product.

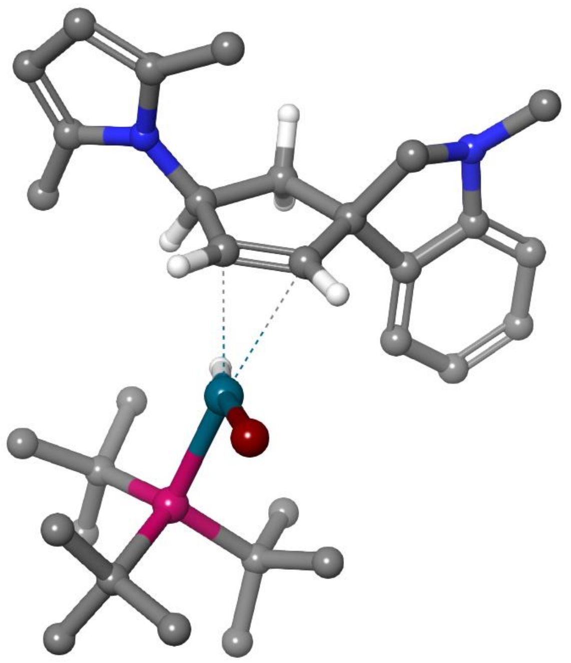

**Figure S6.** Product **9a** (P) resulting from *anti* Heck pathway.

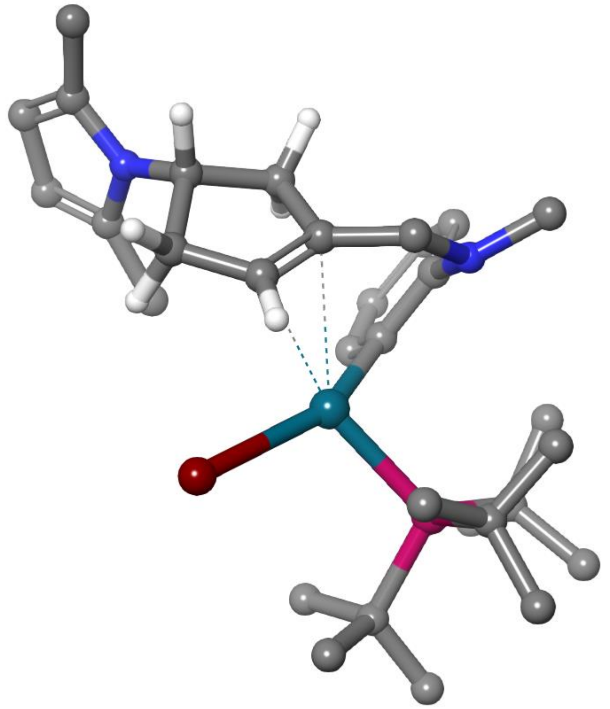

**Figure S7.** π complex intermediate (R) leading to *syn* product.

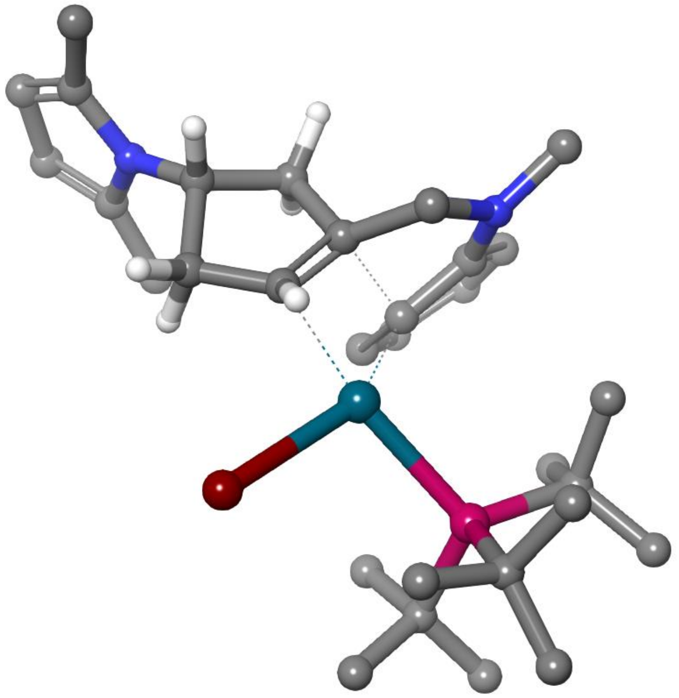

**Figure S8.** Migratory insertion transition step (MI) leading to *syn* product.

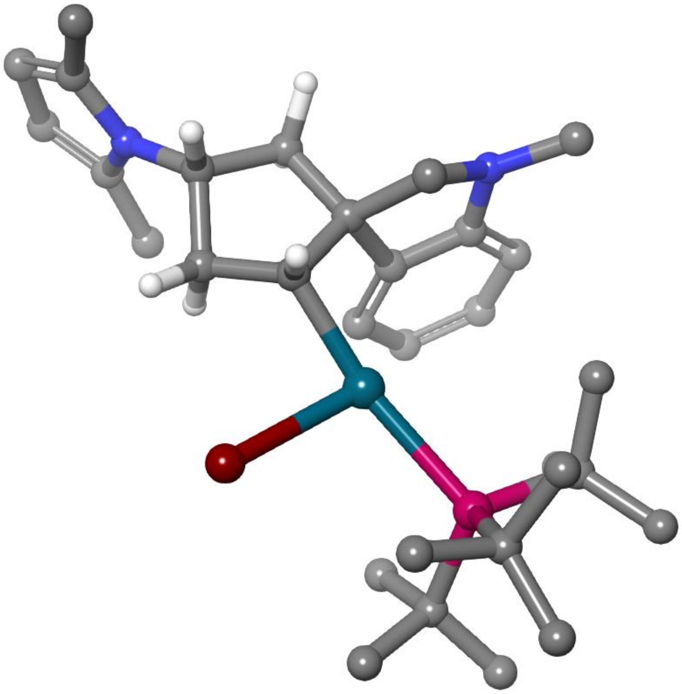

**Figure S9.** σ complex intermediate (I) leading to *syn* product.

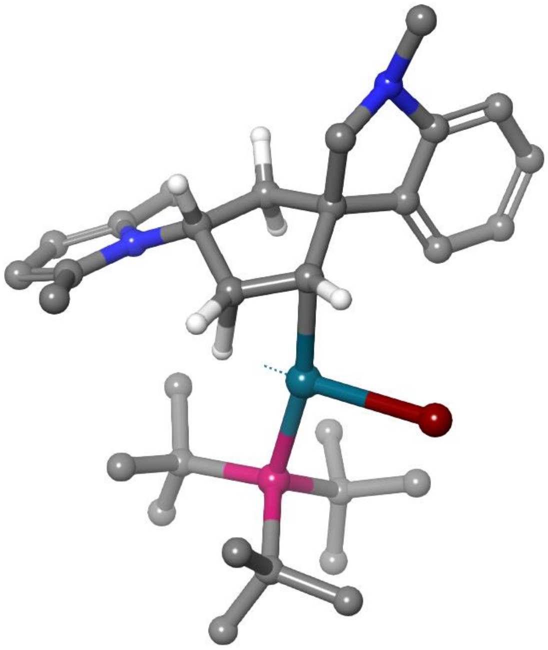

**Figure S10.** β-hydride elimination transition step (BHE) leading to *syn* product.

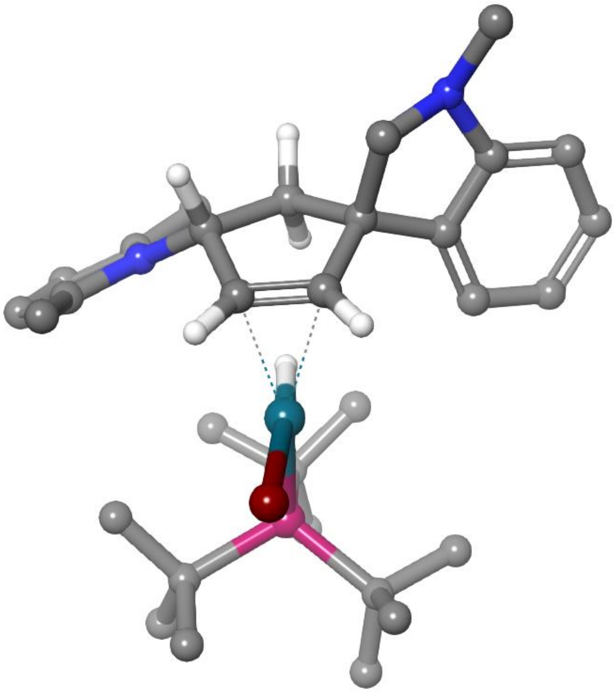

**Figure S11.** Product (P) resulting from *syn* Heck pathway.

NOESY

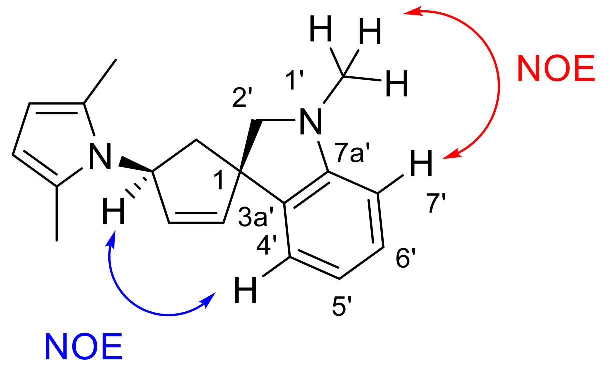


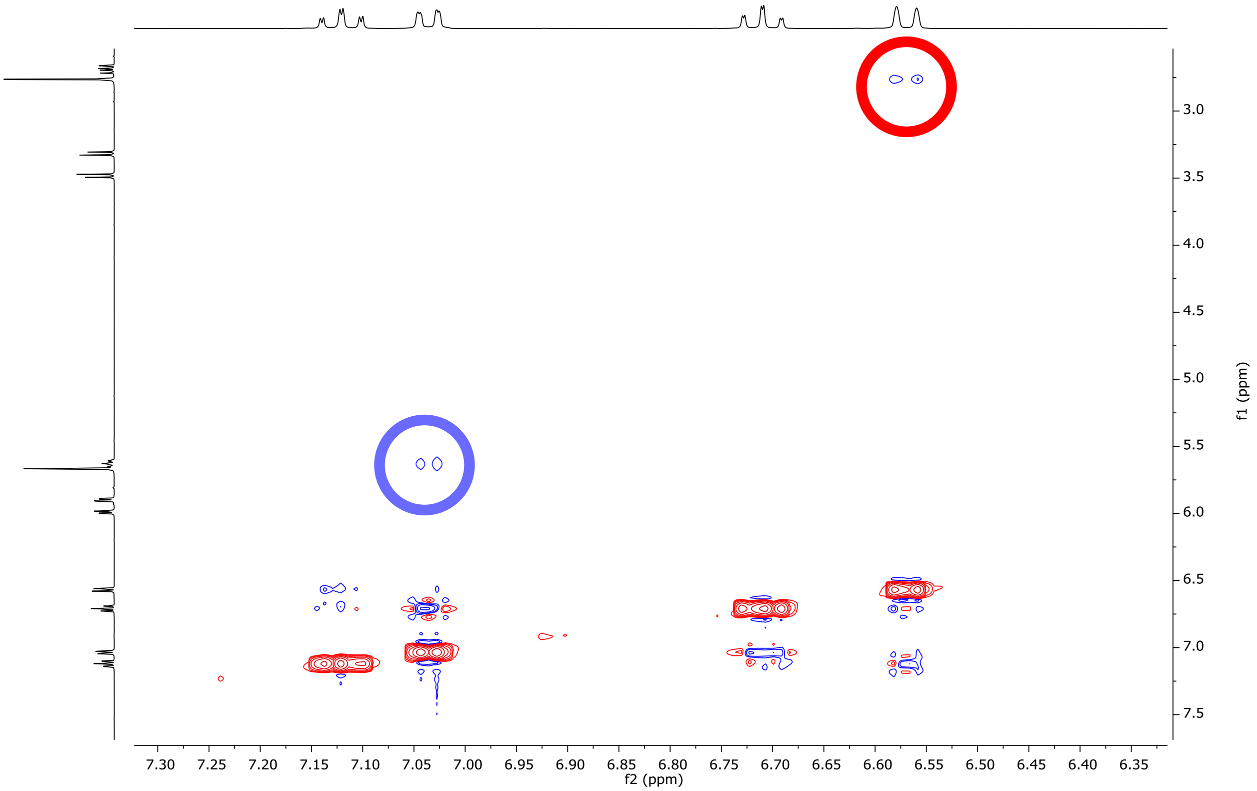


**Figure S12.** 2D- NOESY spectrum of compound **9a** displaying NOE cross-peaks between indoline proton H-4’ and the 2,5-dimethylpyrrole-protected amine α-proton (blue marking). An NOE is also visible between indoline proton H-7’ and the N-methyl protons (red marking).

# X-ray crystallography data

Single crystals of **9m** were investigated with the help of a Rigaku, XtaLAB Synergy, Dualflex, HyPix diffractometer. The crystal was kept at 170.0(2) K during data collection. Using Olex2[15] the structure was solved with the SHELXT[16] structure solution program using Intrinsic Phasing and refined with the olex2.refine[17] refinement package using Levenberg-Marquardt minimization.

**Crystal Data** for For **9m** ($C_{18}H_{21}N_3$, M = 279.39 g/mol): monoclinic, space group *C*2 (no. 5), $a$ = 14.3755(4) Å, $b$ = 6.9430(2) Å, $c$ = 16.3630(4) Å, β = 110.471(3)°, $V$ = 1530.04(7) Å$^3$, $Z$ = 4, $T$ = 170.0(2) K, μ(Cu Kα) = 0.562 mm$^{-1}$, $Dcalc$ = 1.2128 g/cm$^3$, 9236 reflections measured (2Θ ≤ 155.0°), 2989 unique ($R_{int}$ = 0.0251, $R_{sigma}$ = 0.0199) which were used in all calculations. The final $R_1$ was 0.0323 ($I > 2\sigma(I)$) and $wR_2$ was 0.0861 (all data).

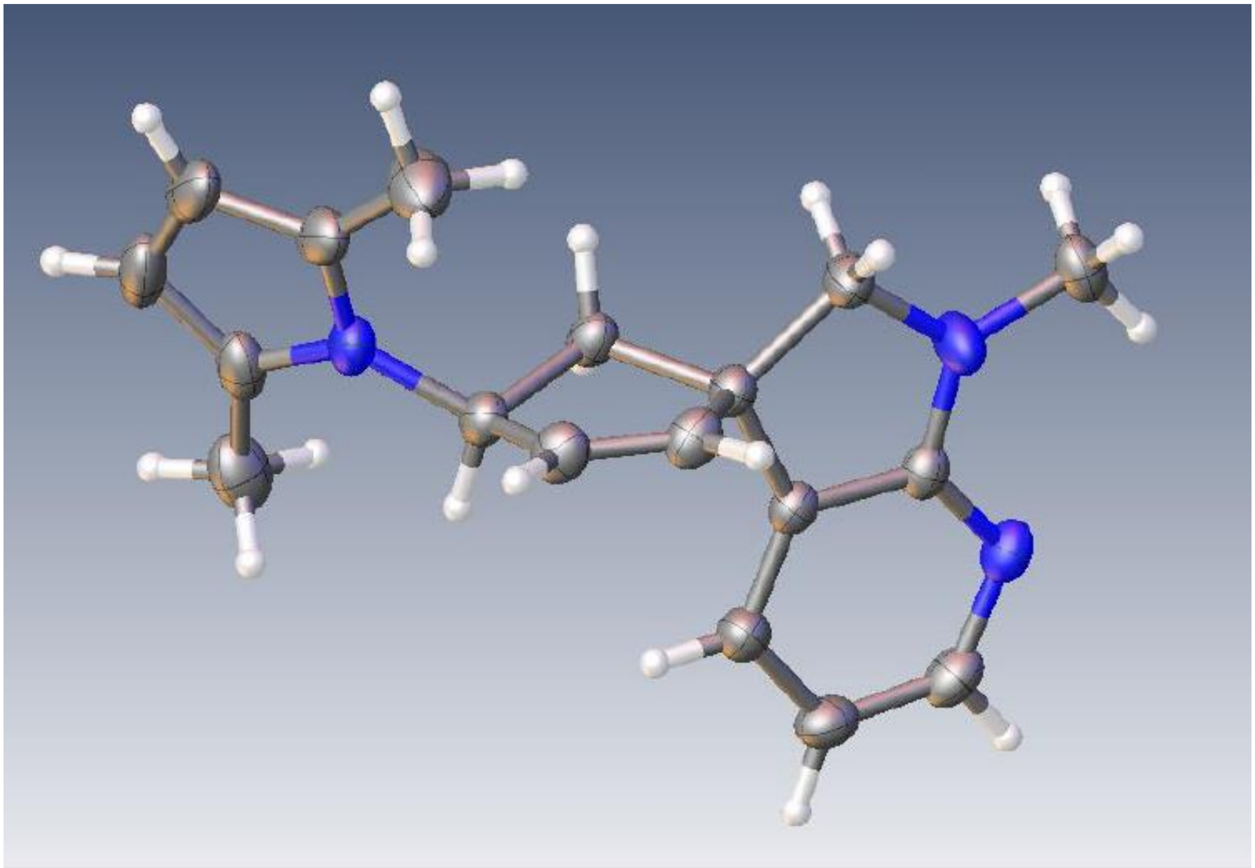

**Figure S13.** ORTEP representation of X-ray crystallography of **9m** with the thermal ellipsoid drawn at 50% probability.

# NMR-spectra of compounds **5** – **12**

**<u>(*R*)-1-(3-(chloromethyl)cyclopent-3-en-1-yl)-2,5-dimethyl-1H-pyrrole (5)</u>**

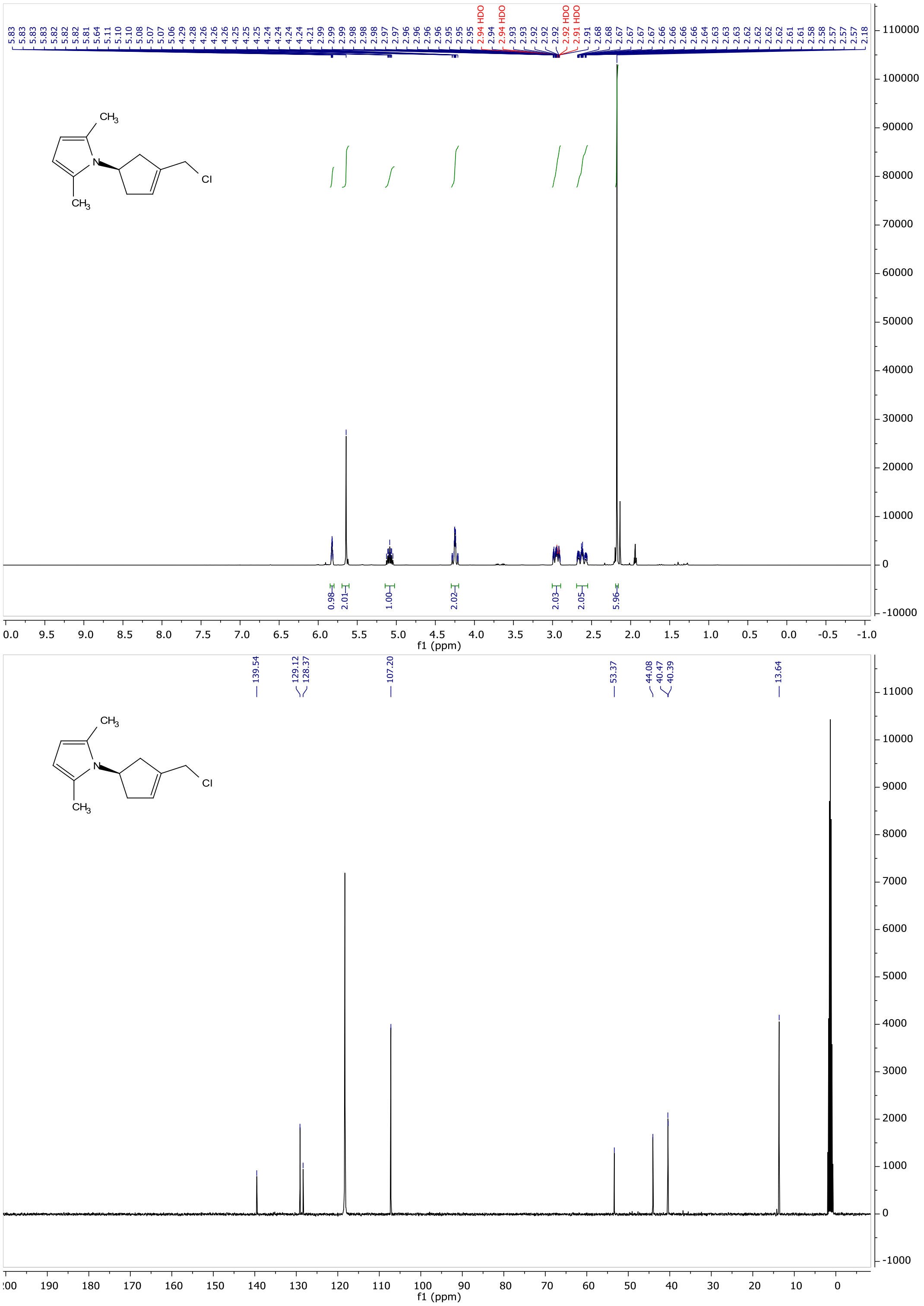


**Figure S14.** $^{1}H$ and $^{13}C\{^{1}H\}$ NMR spectra of **5** in $CD_3CN$.

**(*R*)-*N*- 2-bromo- -((4-(2,5-dimethyl-1H-pyrrol-1-yl)cyclopent-1-en-1-yl)methyl)aniline (6)**

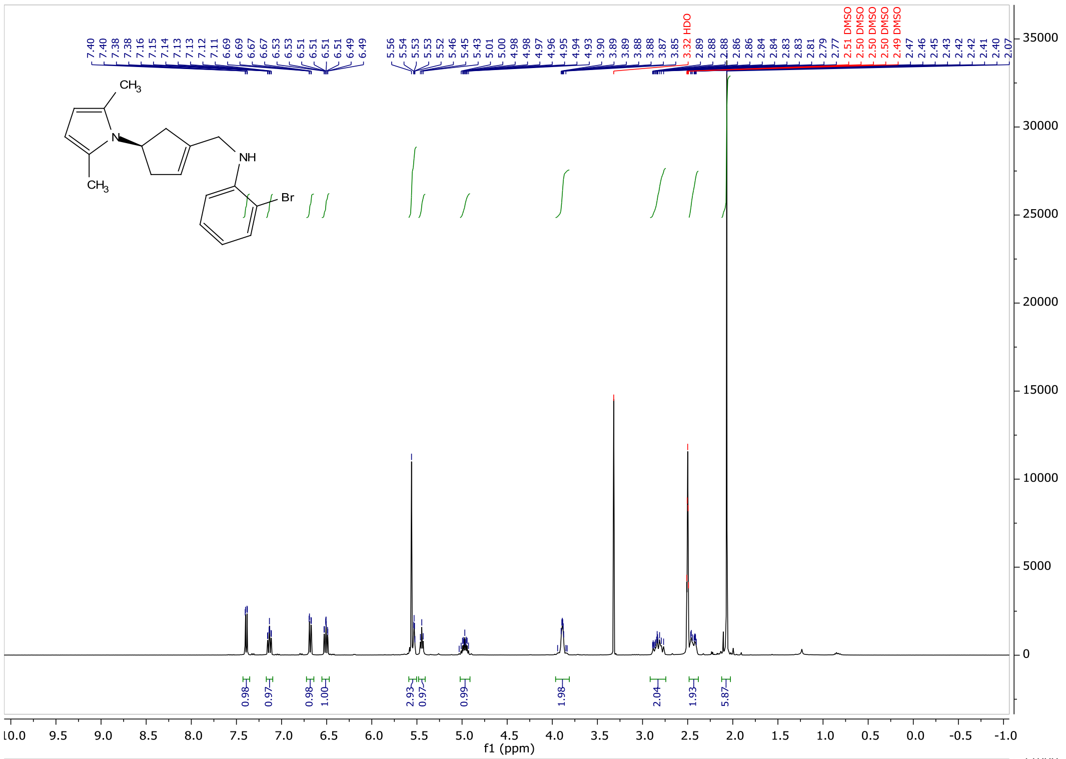

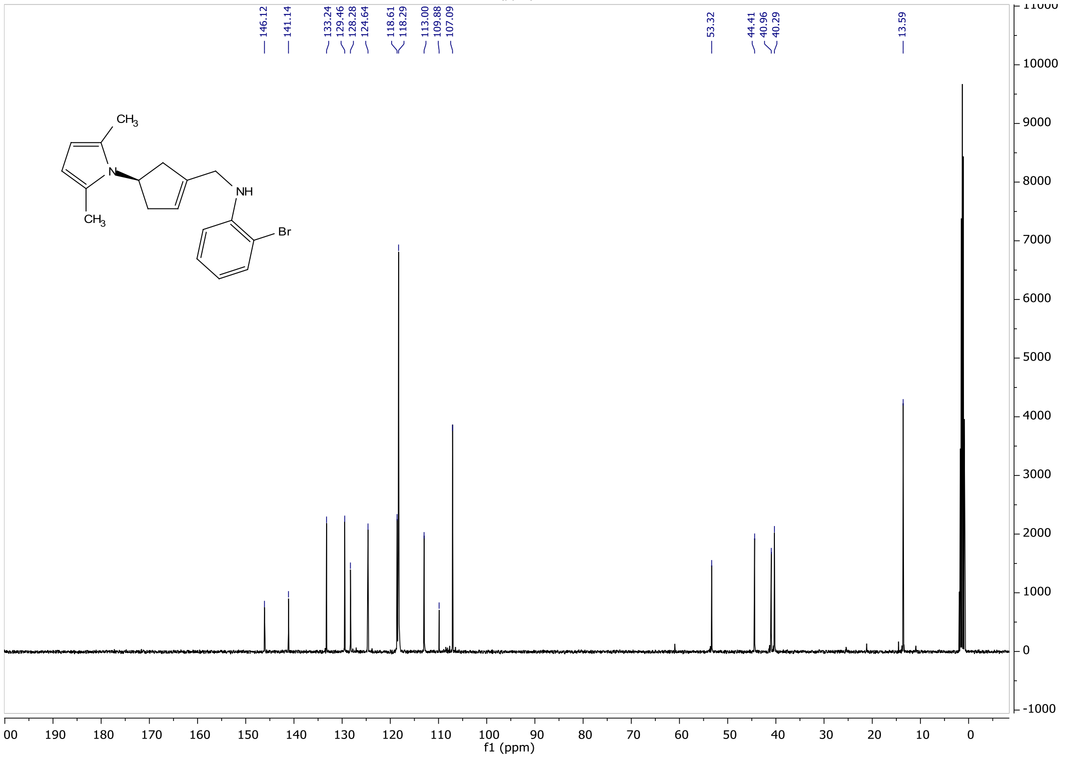

**Figure S15.** $^{1}H$ and $^{13}C\{^{1}H\}$ NMR spectra of **6** in DMSO-d6.

**(1*R*,4*S*)-4-(2,5-dimethyl-1H-pyrrol-1-yl)spiro[cyclopentane-1,3'-indolin]-2-ene (7)**

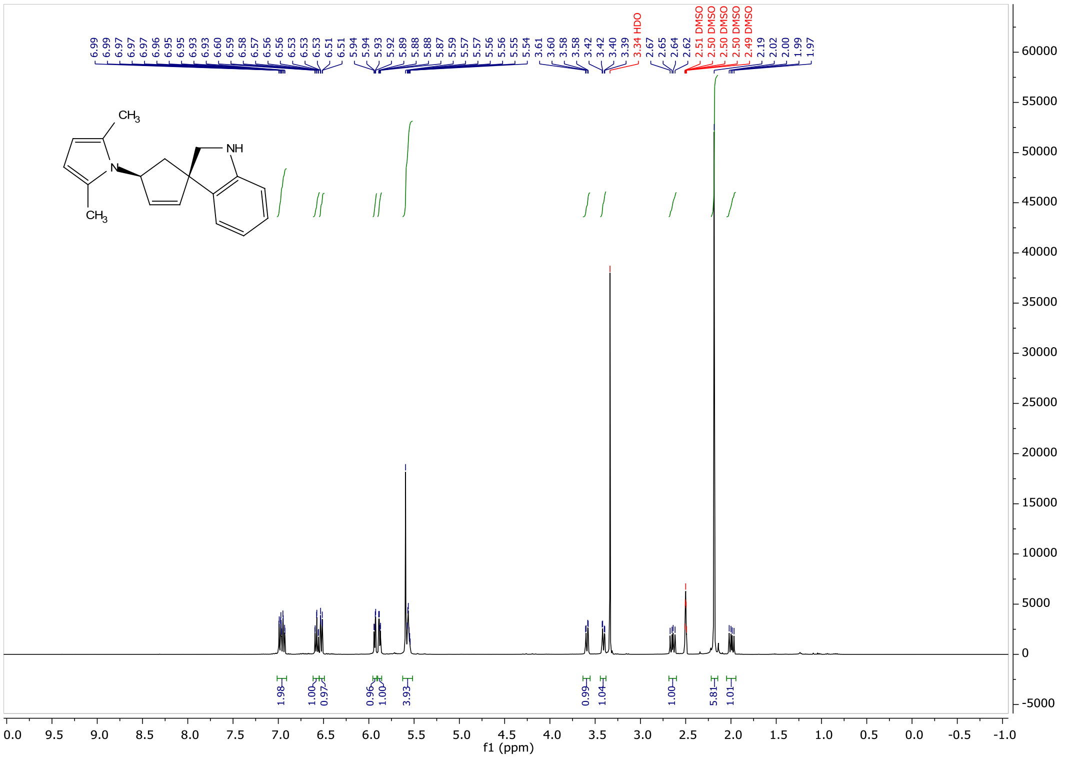

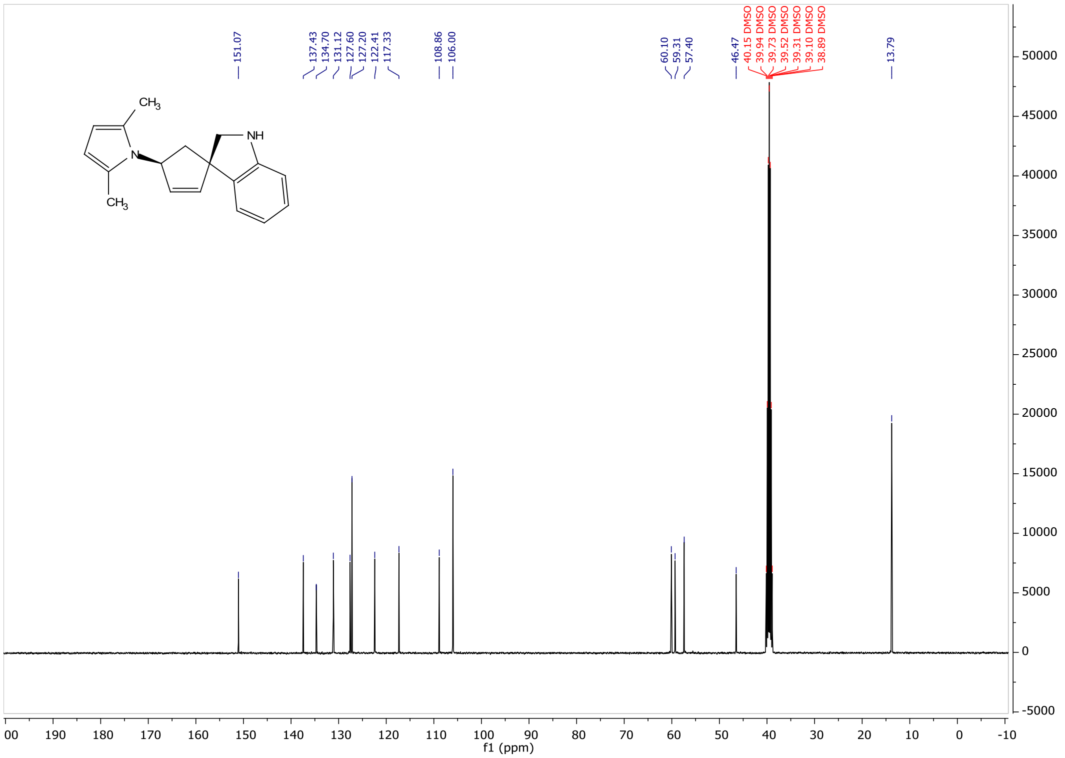

**Figure S16.** $^{1}H$ and $^{13}C\{^{1}H\}$ NMR spectra of **7** in DMSO-d6.

## (*R*)-2-bromo-*N*-((4-(2,5-dimethyl-1H-pyrrol-1-yl)cyclopent-1-en-1-yl)methyl)-*N*-methylaniline (8a)

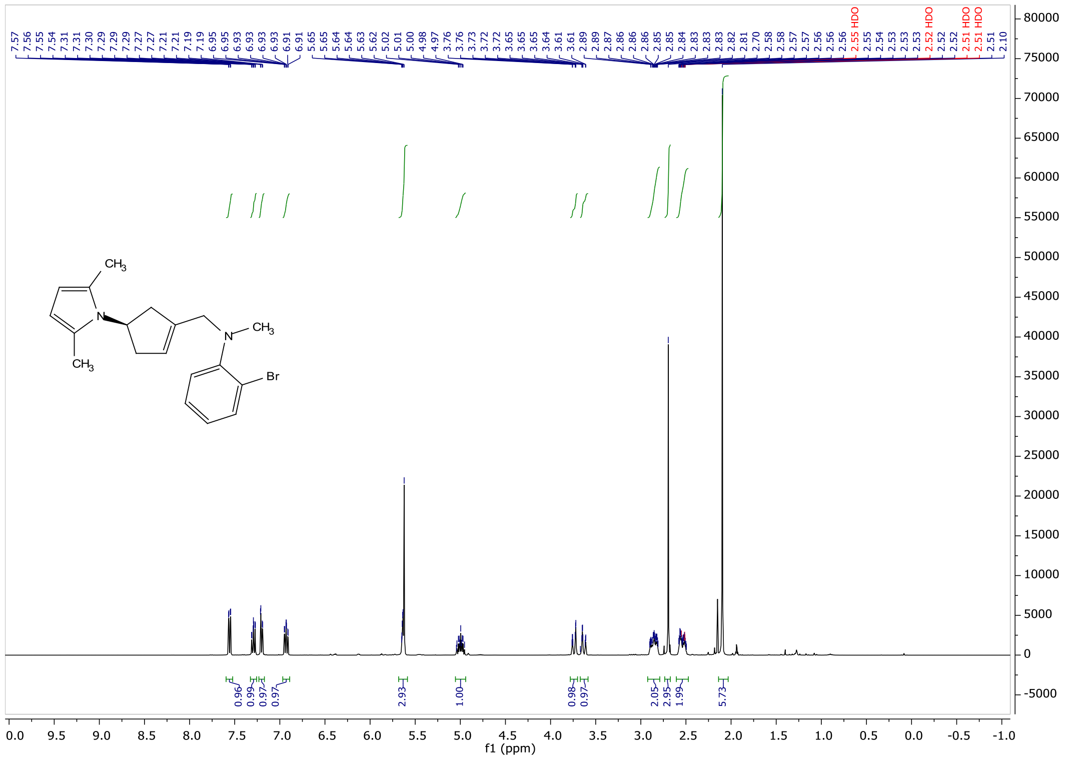


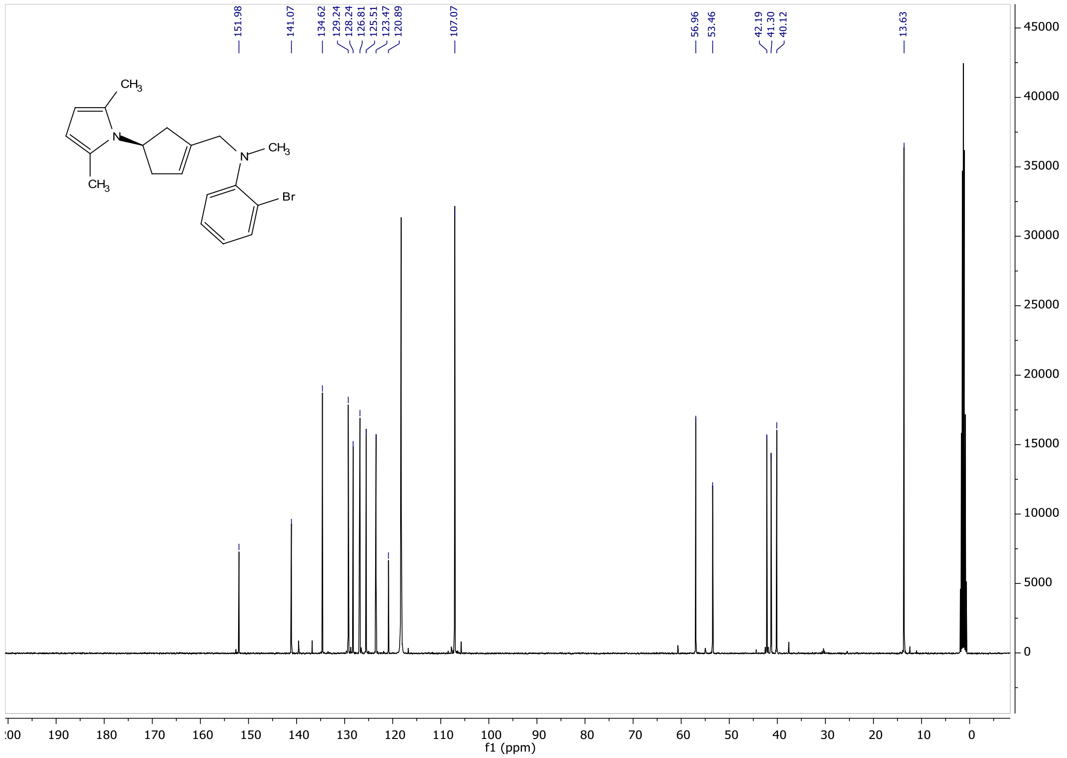


**Figure S17.** $^{1}$H and $^{13}$C{$^{1}$H} NMR spectra of **8a** in $CD_3CN$.

**(*R*)-3-bromo-4-(((4-(2,5-dimethyl-1H-pyrrol-1-yl)cyclopent-1-en-1-yl)methyl)(methyl)amino)benzonitrile (8b)**

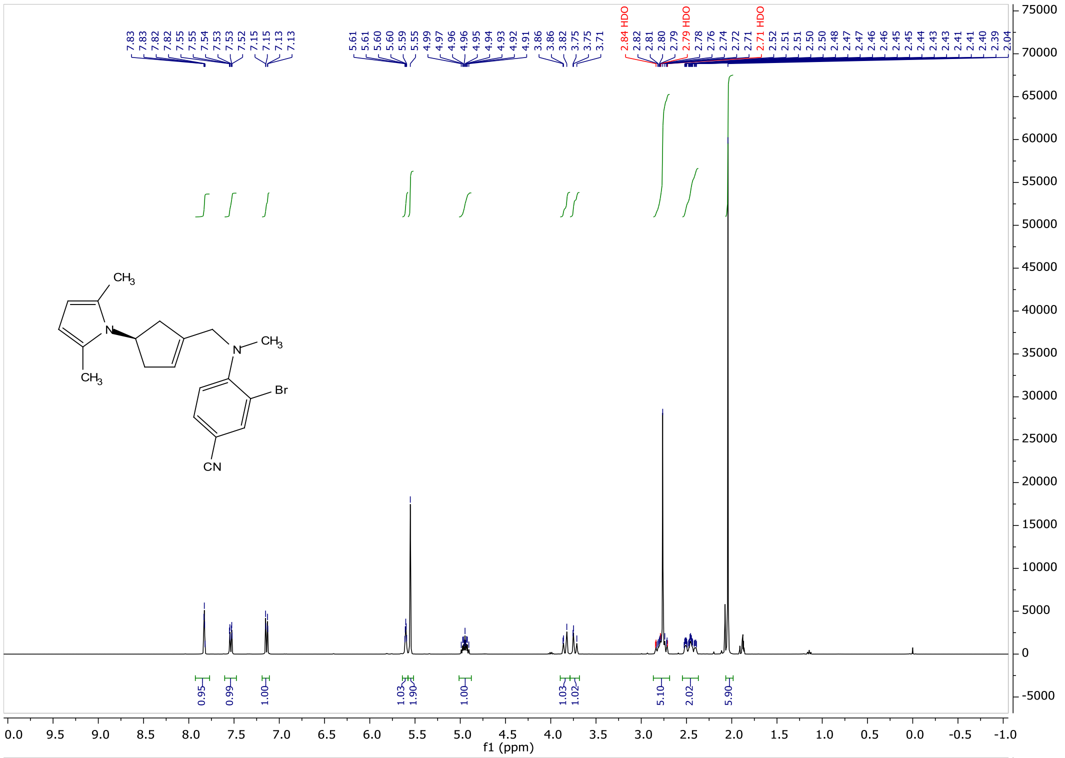

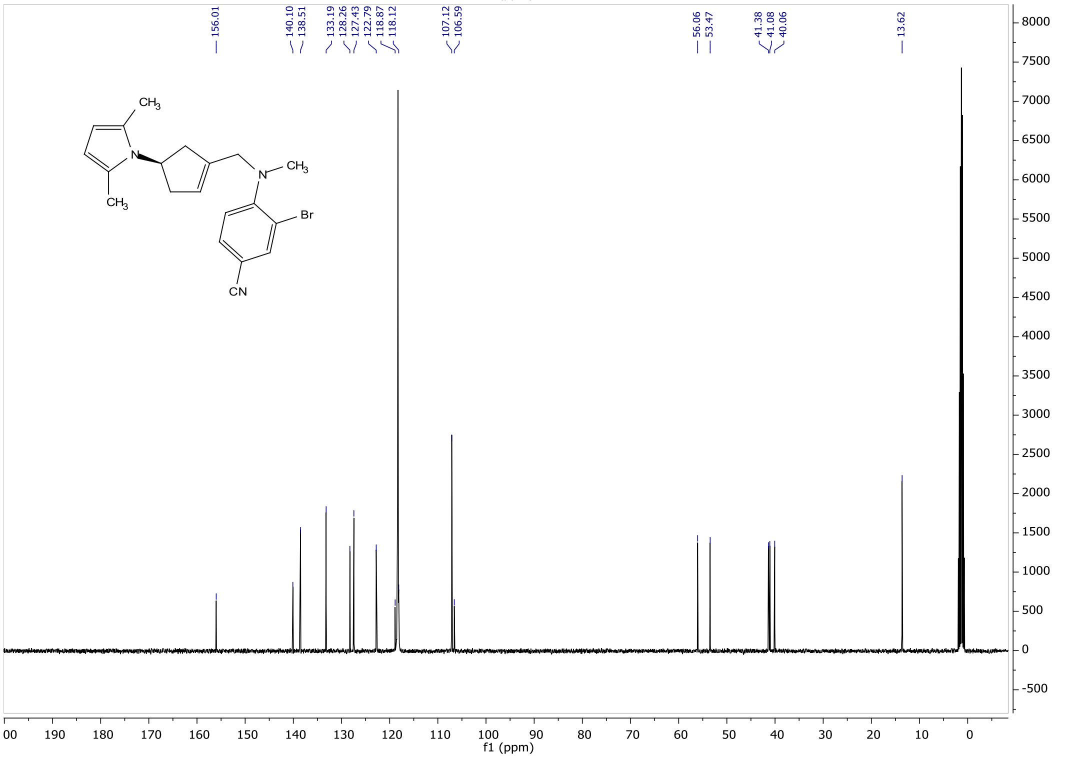

**Figure S18.** $^1$H and $^{13}$C{$^1$H} NMR spectra of **8b** in $CD_3CN$.

**(*R*)-2-bromo-4-chloro-*N*-((4-(2,5-dimethyl-1H-pyrrol-1-yl)cyclopent-1-en-1-yl)methyl)-*N*-methylaniline (8c)**

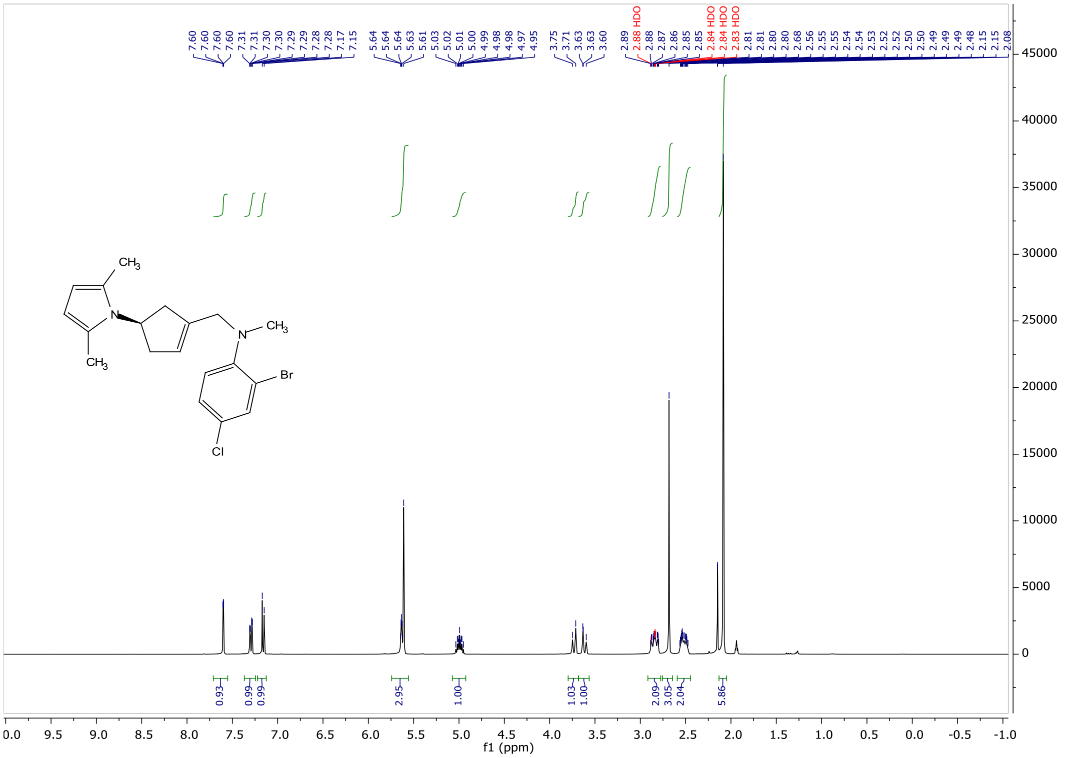

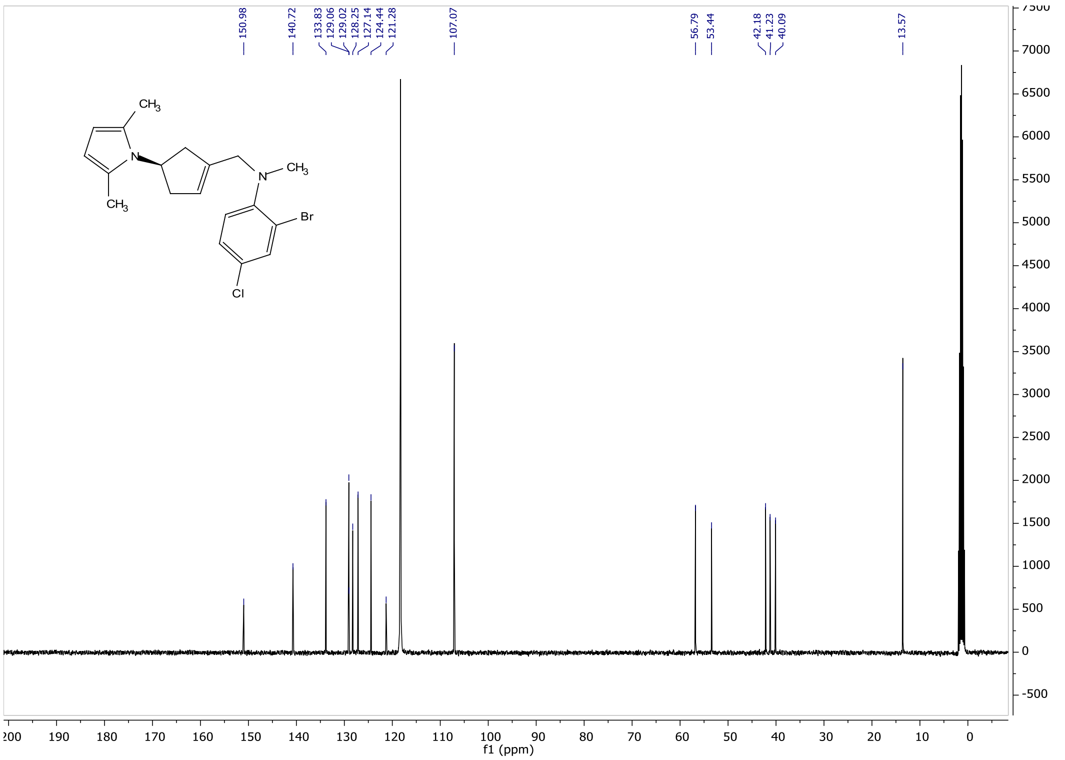

**Figure S19.** $^{1}H$ and $^{13}C\{^{1}H\}$ NMR spectra of **8c** in $CD_3CN$.

## (*R*)-2-bromo-*N*-((4-(2,5-dimethyl-1H-pyrrol-1-yl)cyclopent-1-en-1-yl)methyl)-*N*-methyl-5-(trifluoromethyl)aniline (8d)

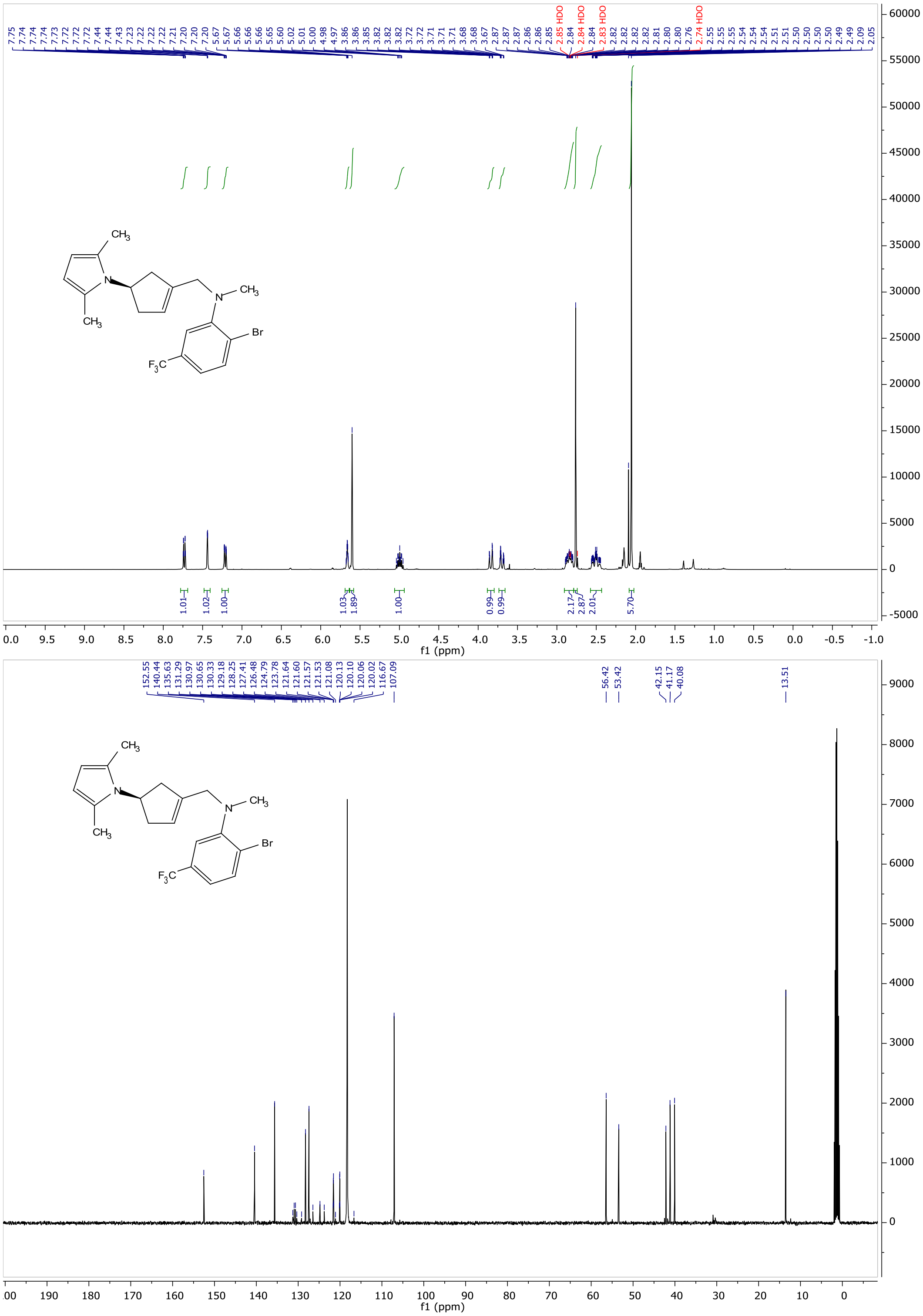


**Figure S20.** $^{1}H$ and $^{13}C\{^{1}H\}$ NMR spectra of **8d** in $CD_3CN$.

**(*R*)-2-bromo-*N*-((4-(2,5-dimethyl-1H-pyrrol-1-yl)cyclopent-1-en-1-yl)methyl)-*N*,3-dimethylaniline (8e)**

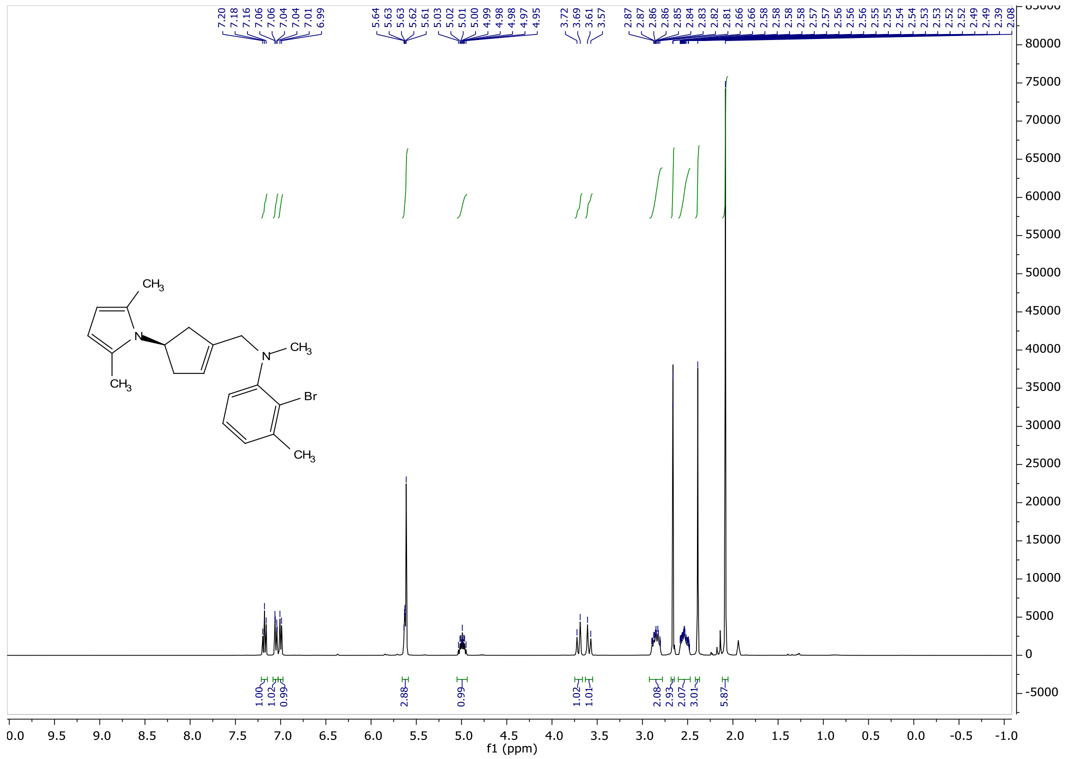

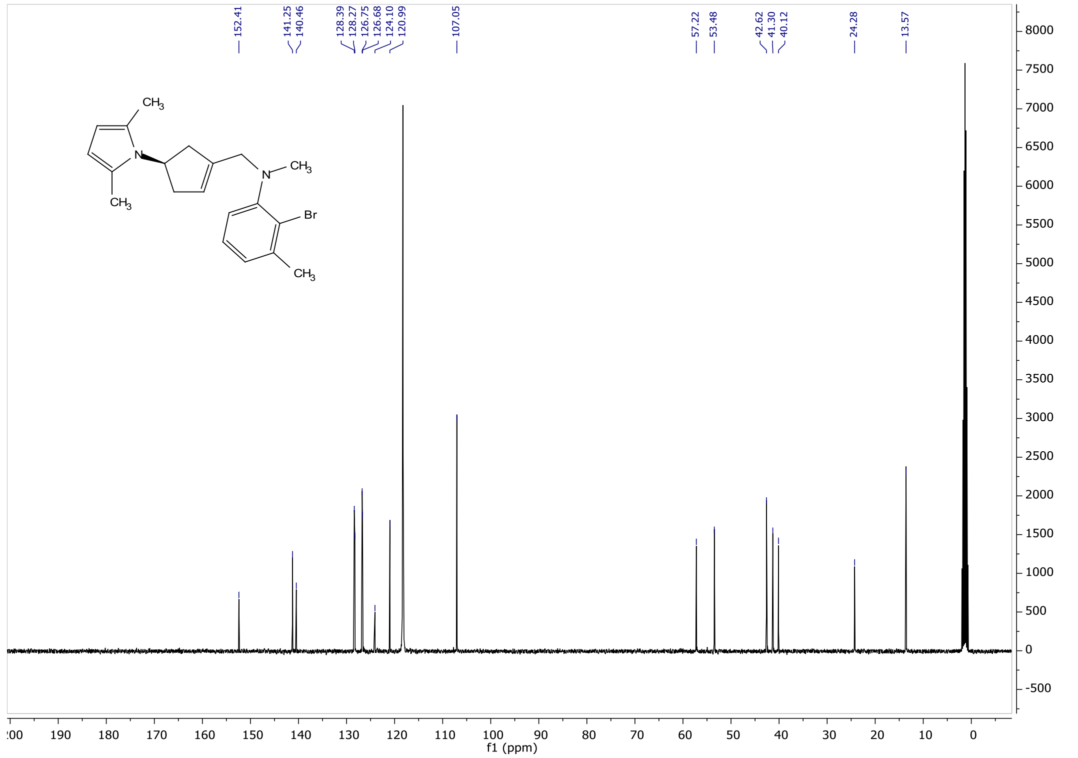

**Figure S21.** $^{1}H$ and $^{13}C\{^{1}H\}$ NMR spectra of **8e** in $CD_3CN$.

**(*R*)-2-bromo-*N*-((4-(2,5-dimethyl-1H-pyrrol-1-yl)cyclopent-1-en-1-yl)methyl)-*N*,4-dimethylaniline (8f)**

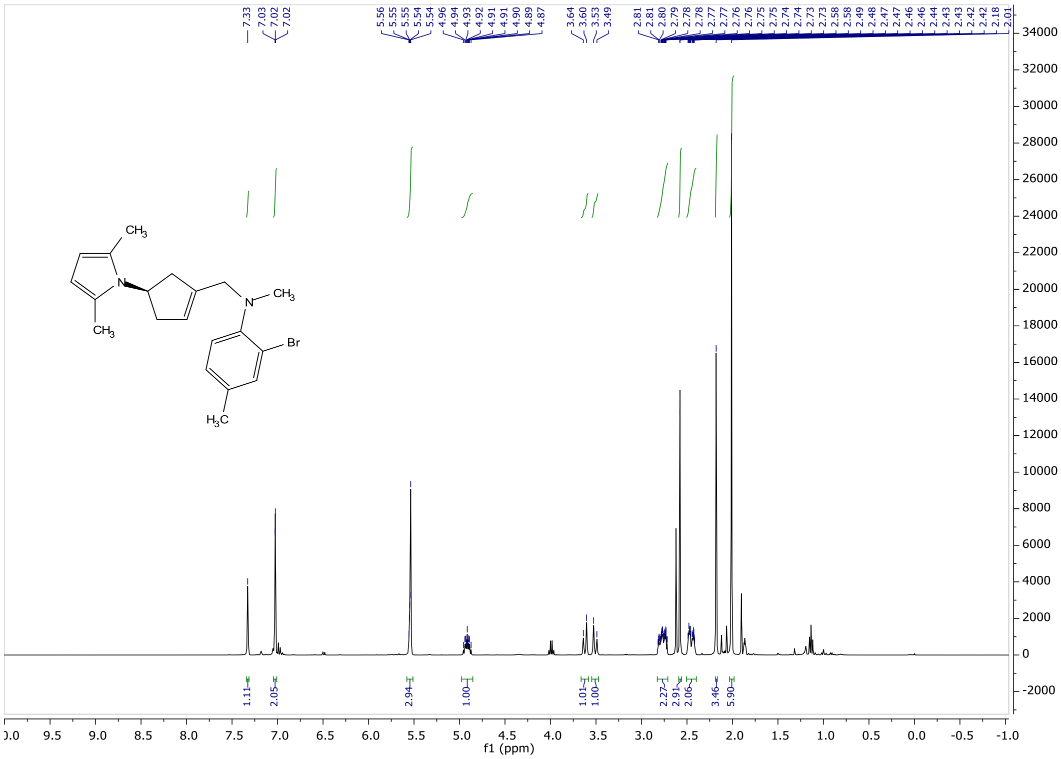

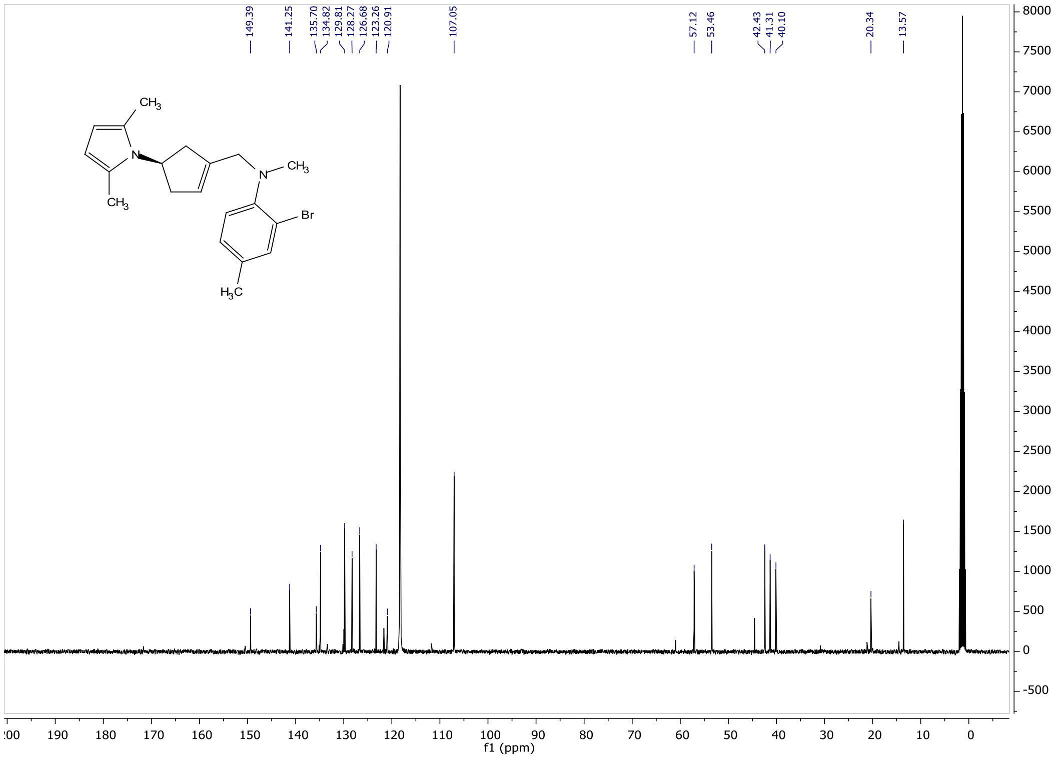

**Figure S22.** $^{1}H$ and $^{13}C\{^{1}H\}$ NMR spectra of **8f** in $CD_3CN$.

**(*R*)-2-bromo-*N*-((4-(2,5-dimethyl-1H-pyrrol-1-yl)cyclopent-1-en-1-yl)methyl)-*N*,5-dimethylaniline (8g)**

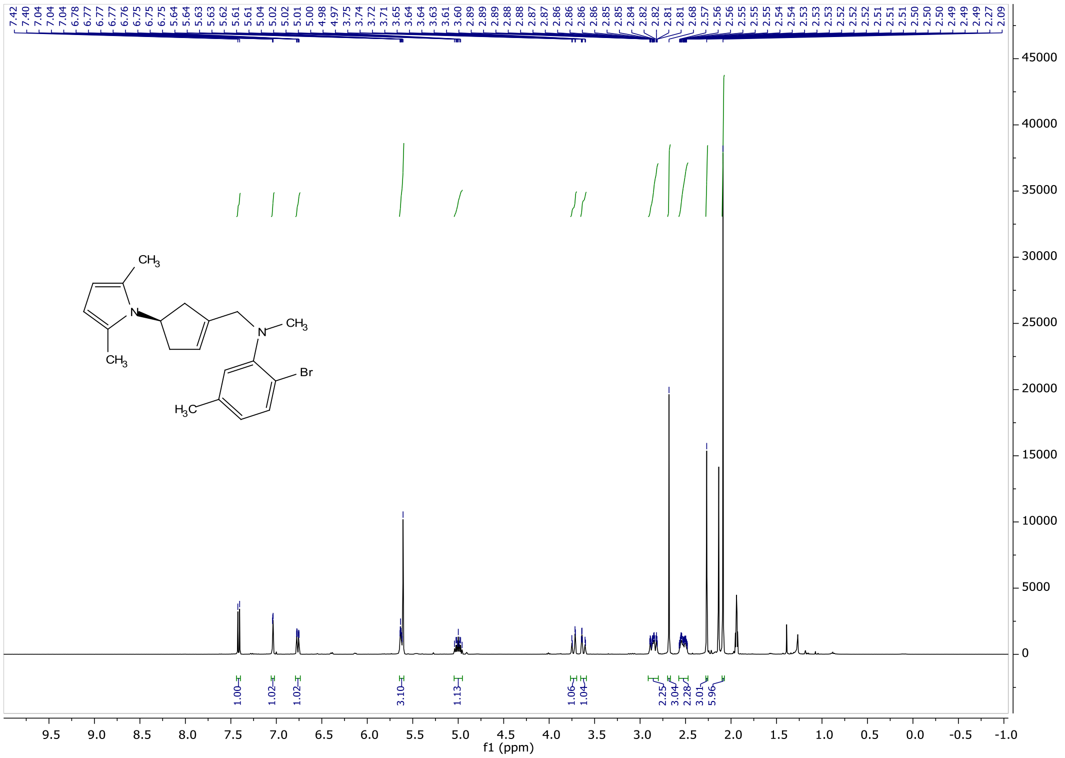

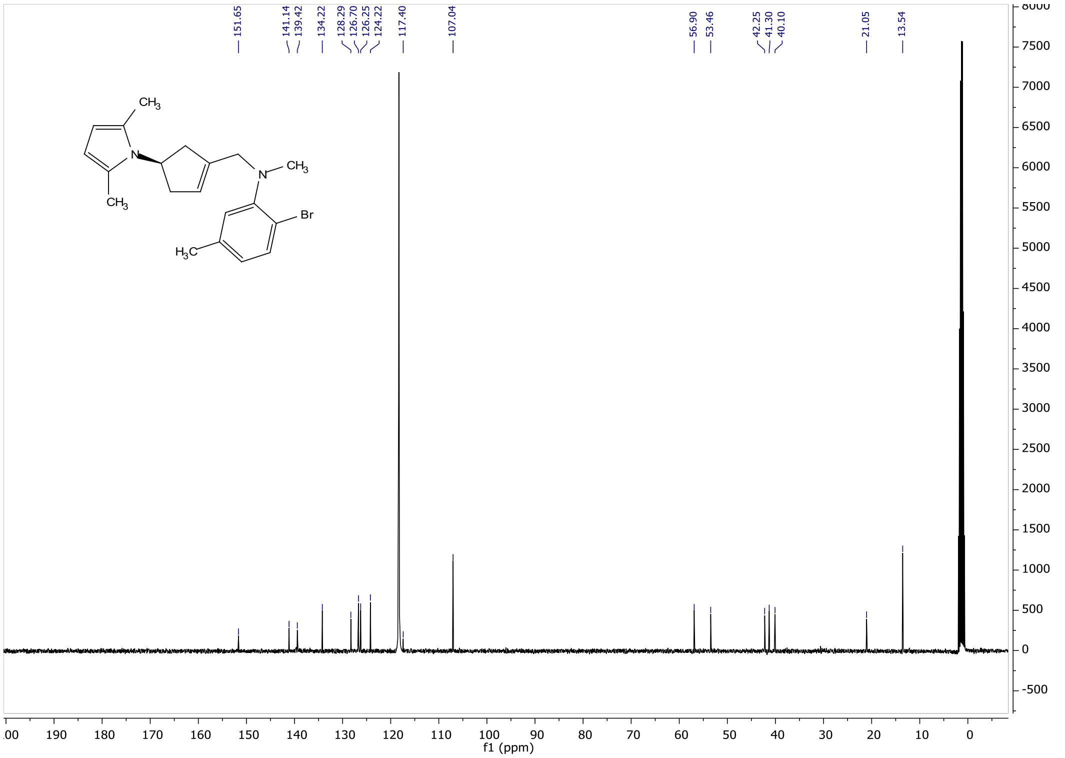

**Figure S23.** $^{1}$H and $^{13}$C{$^{1}$H} NMR spectra of **8g** in $CD_3CN$.

**(*R*)-2-bromo-*N*-((4-(2,5-dimethyl-1H-pyrrol-1-yl)cyclopent-1-en-1-yl)methyl)-*N*,6-dimethylaniline (8h)**

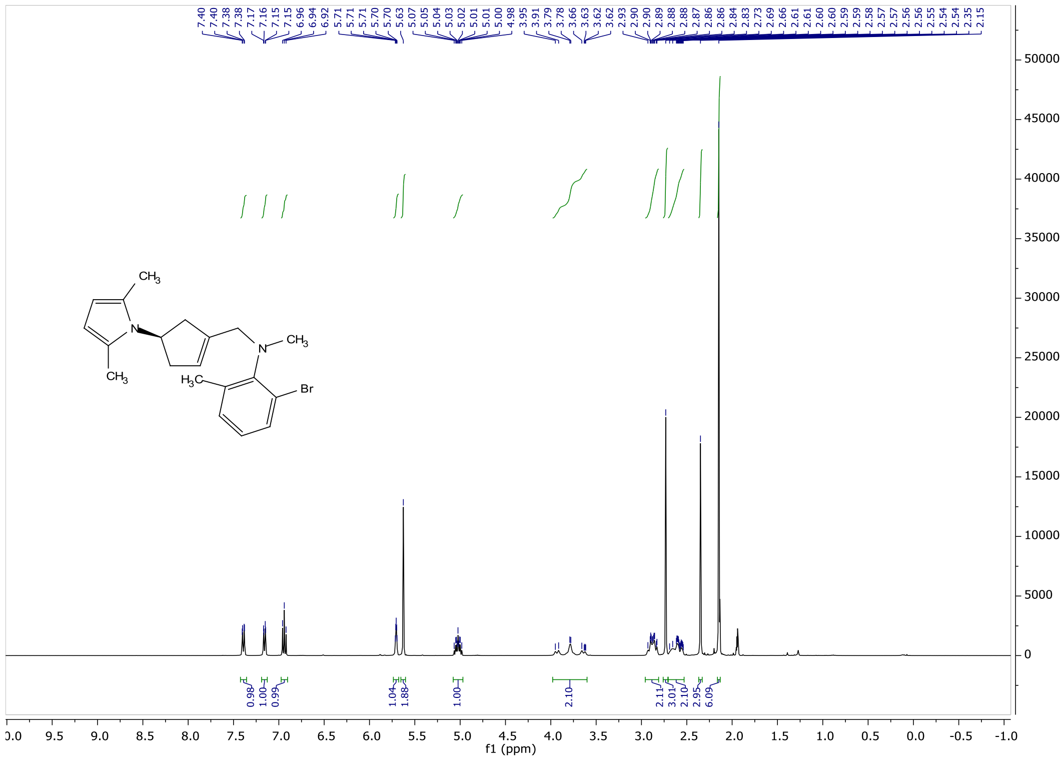

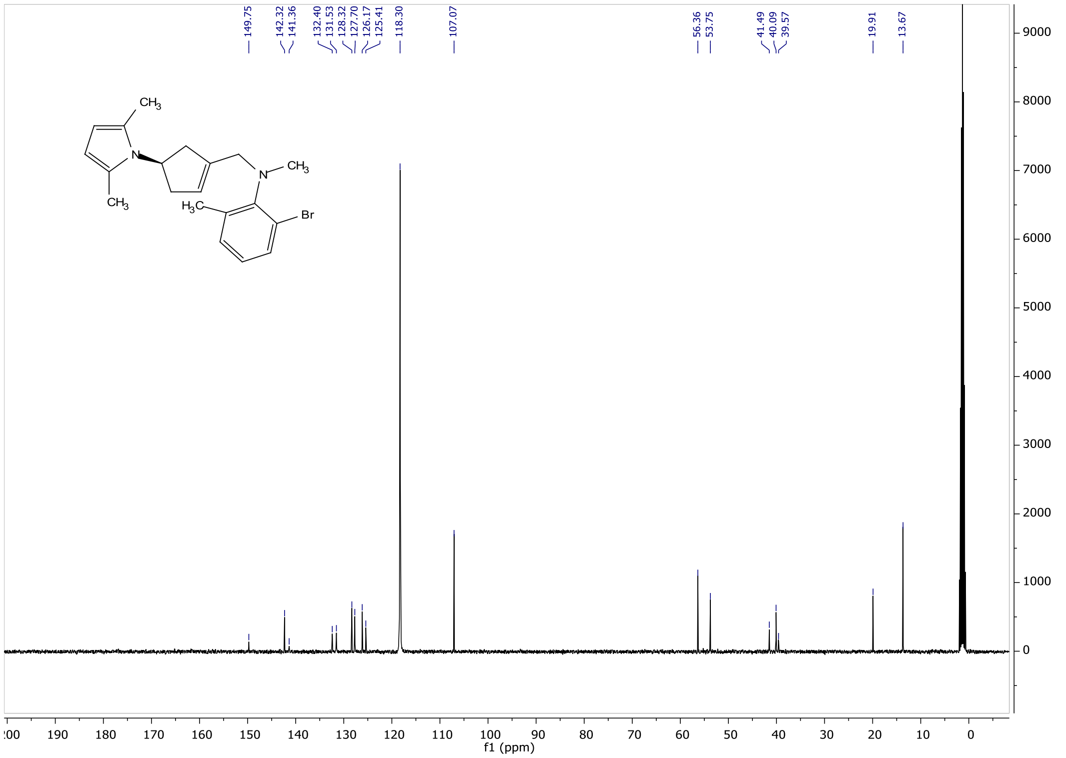

**Figure S24.** $^{1}H$ and $^{13}C\{^{1}H\}$ NMR spectra of **8h** in $CD_3CN$.

**(*R*)-2-bromo-*N*-((4-(2,5-dimethyl-1H-pyrrol-1-yl)cyclopent-1-en-1-yl)methyl)-4-fluoro-*N*-methylaniline (8i)**

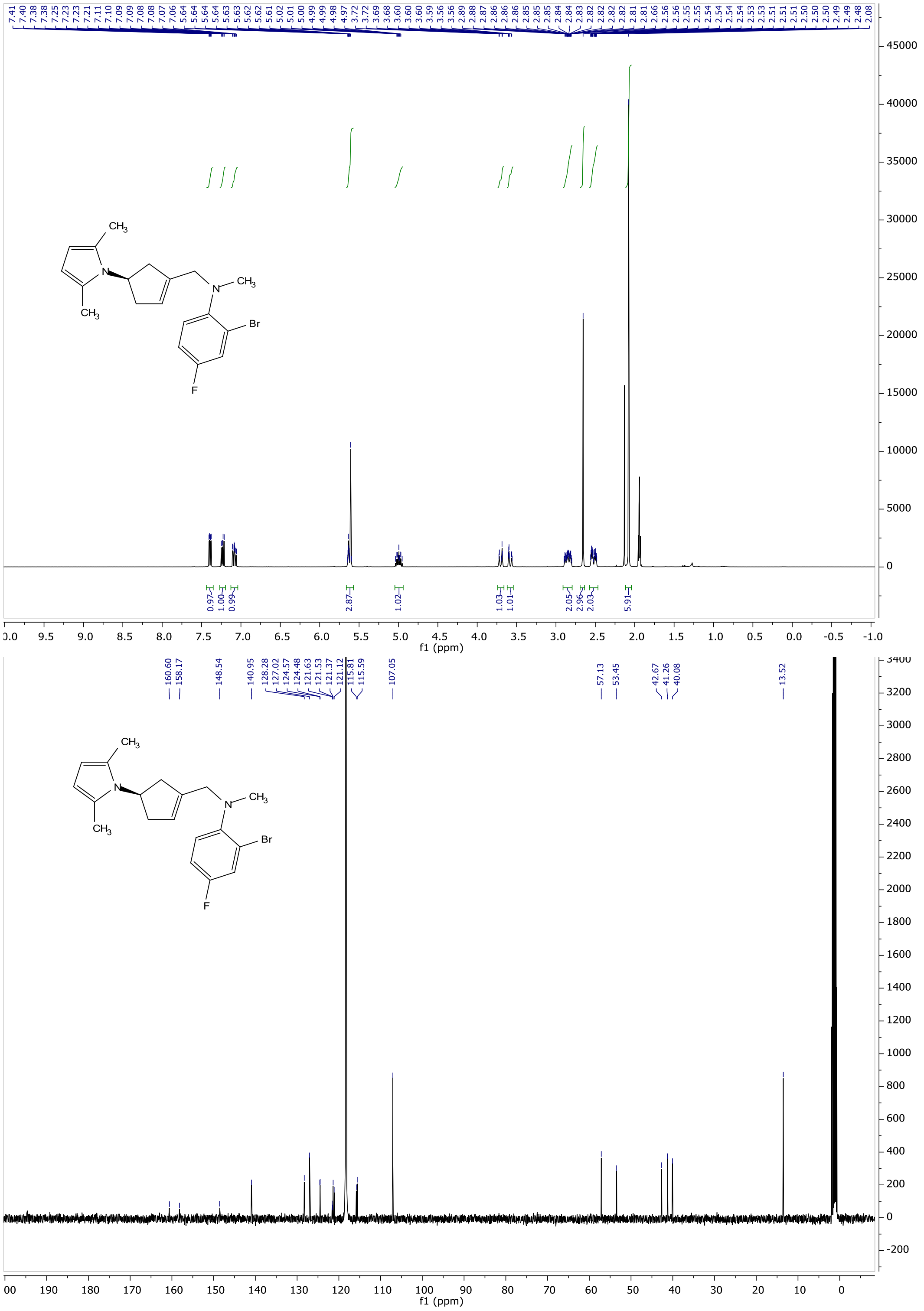


**Figure S25.** $^{1}H$ and $^{13}C\{^{1}H\}$ NMR spectra of **8i** in $CD_3CN$.

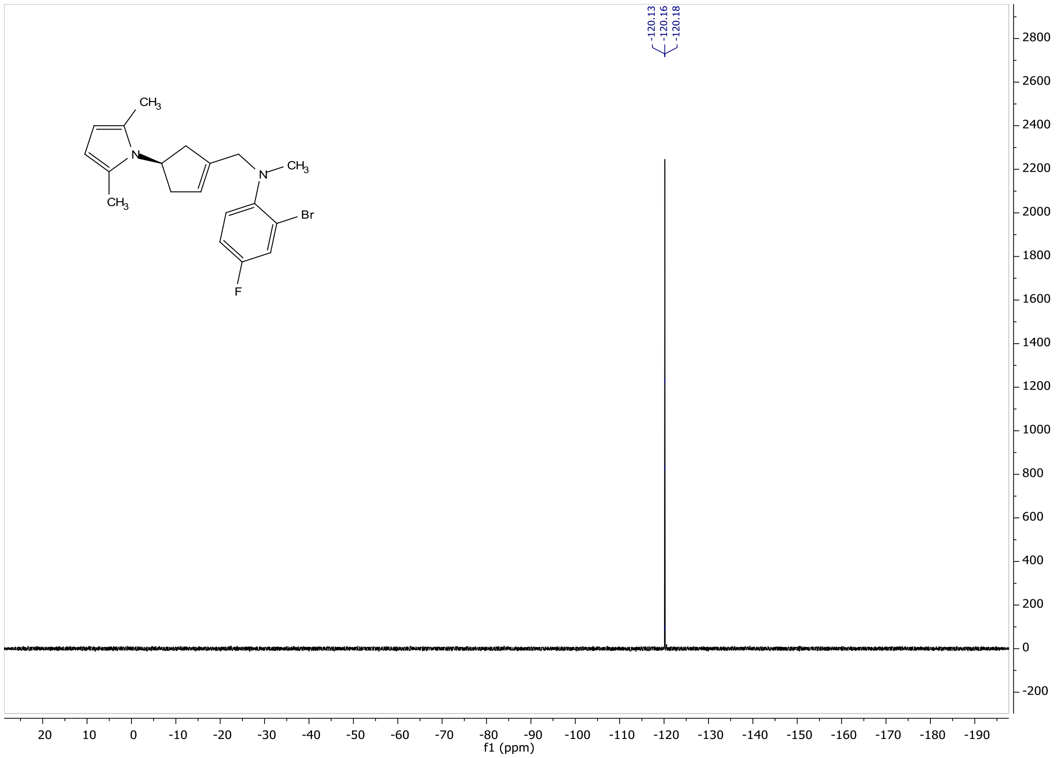


**Figure S26.** $^{19}F$ spectrum of **8i** in $CD_3CN$.

**(*R*)-2,4-dibromo-*N*-((4-(2,5-dimethyl-1H-pyrrol-1-yl)cyclopent-1-en-1-yl)methyl)-*N*-methylaniline (8j)**

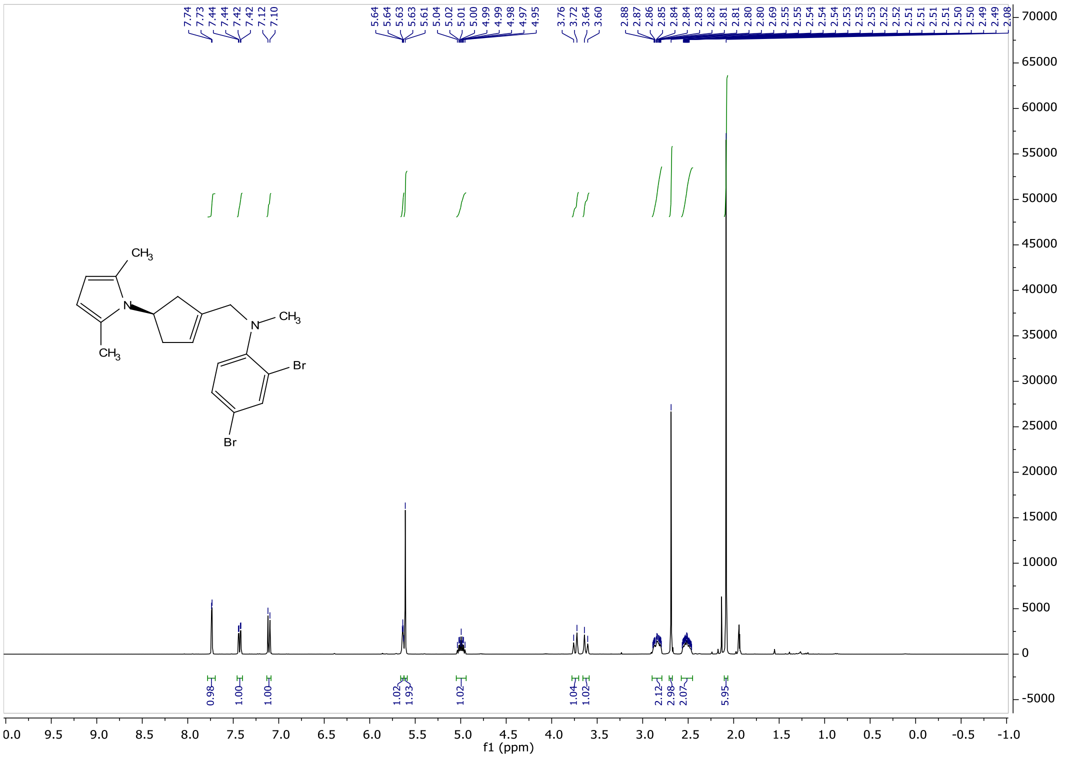


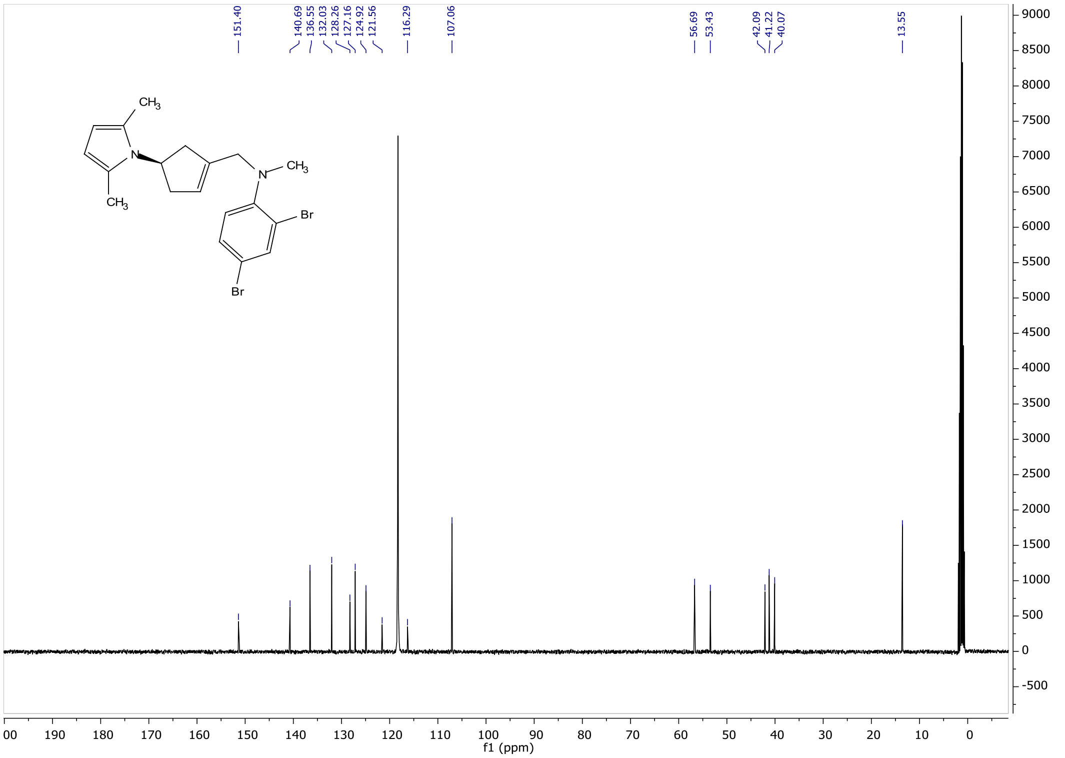


**Figure S27.** $^{1}H$ and $^{13}C\{^{1}H\}$ NMR spectra of **8j** in $CD_3CN$.

**(*R*)-2,5-dibromo-*N*-((4-(2,5-dimethyl-1H-pyrrol-1-yl)cyclopent-1-en-1-yl)methyl)-*N*-methylaniline (8k)**

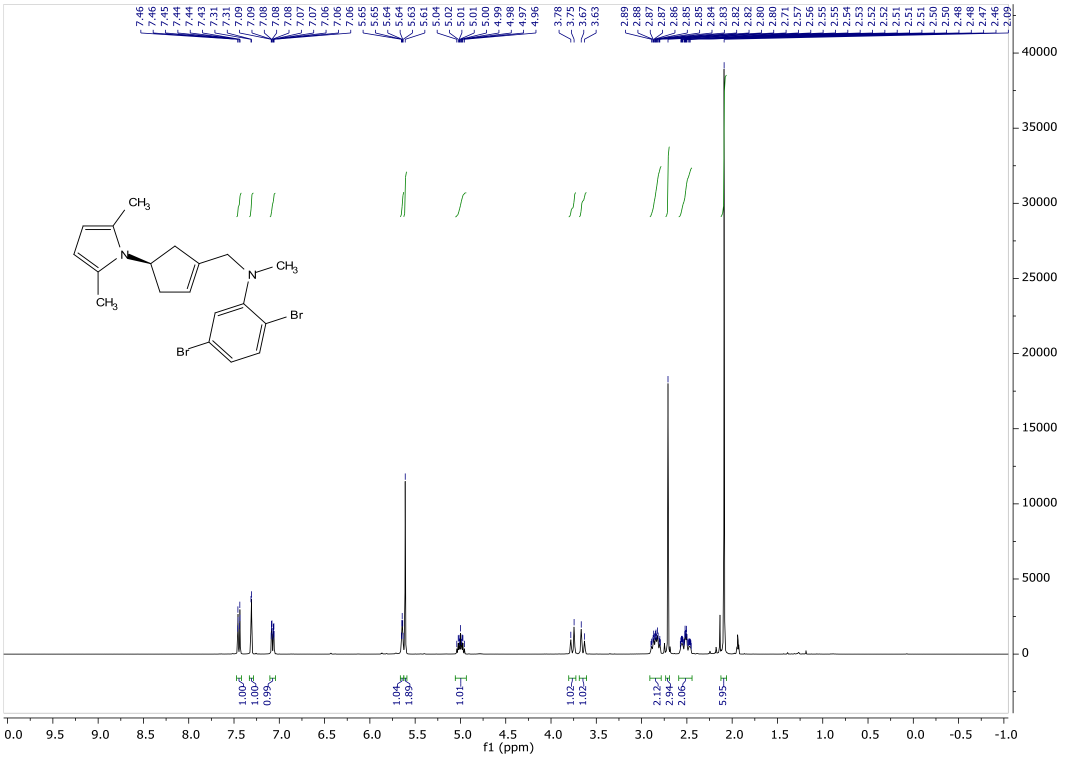

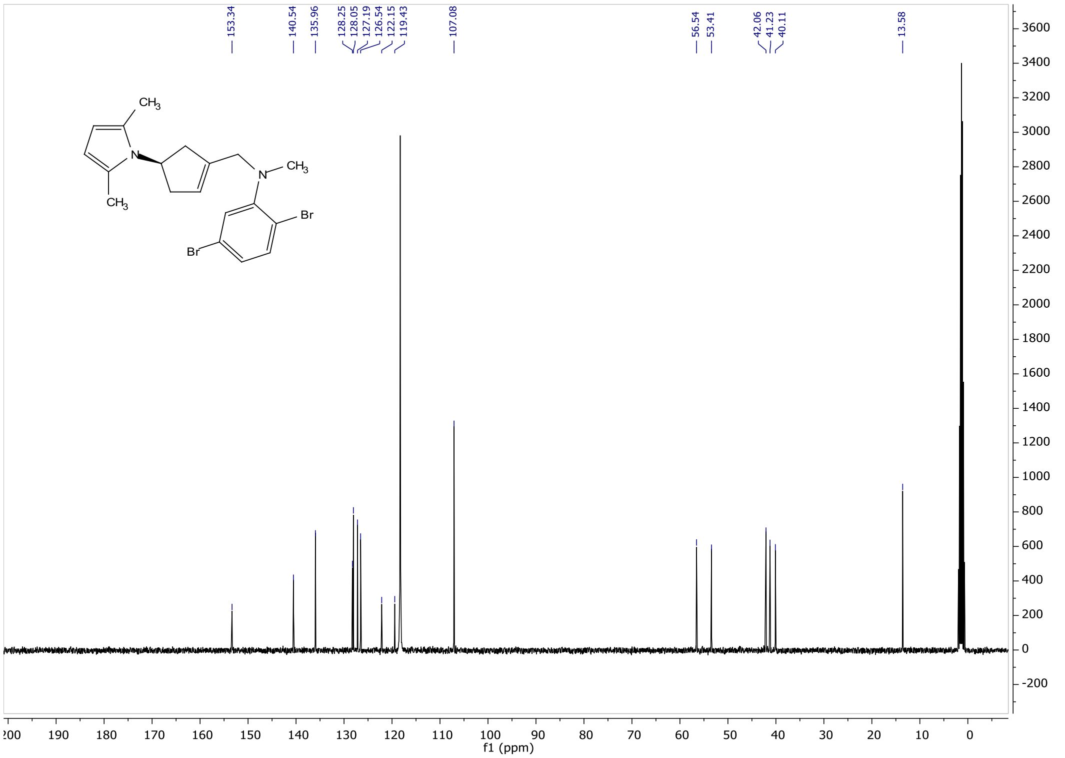

**Figure S28.** $^{1}H$ and $^{13}C\{^{1}H\}$ NMR spectra of **8k** in $CD_3CN$.

**(*R*)-2-bromo-*N*-((4-(2,5-dimethyl-1H-pyrrol-1-yl)cyclopent-1-en-1-yl)methyl)-5-methoxy-*N*-methylaniline (8l)**

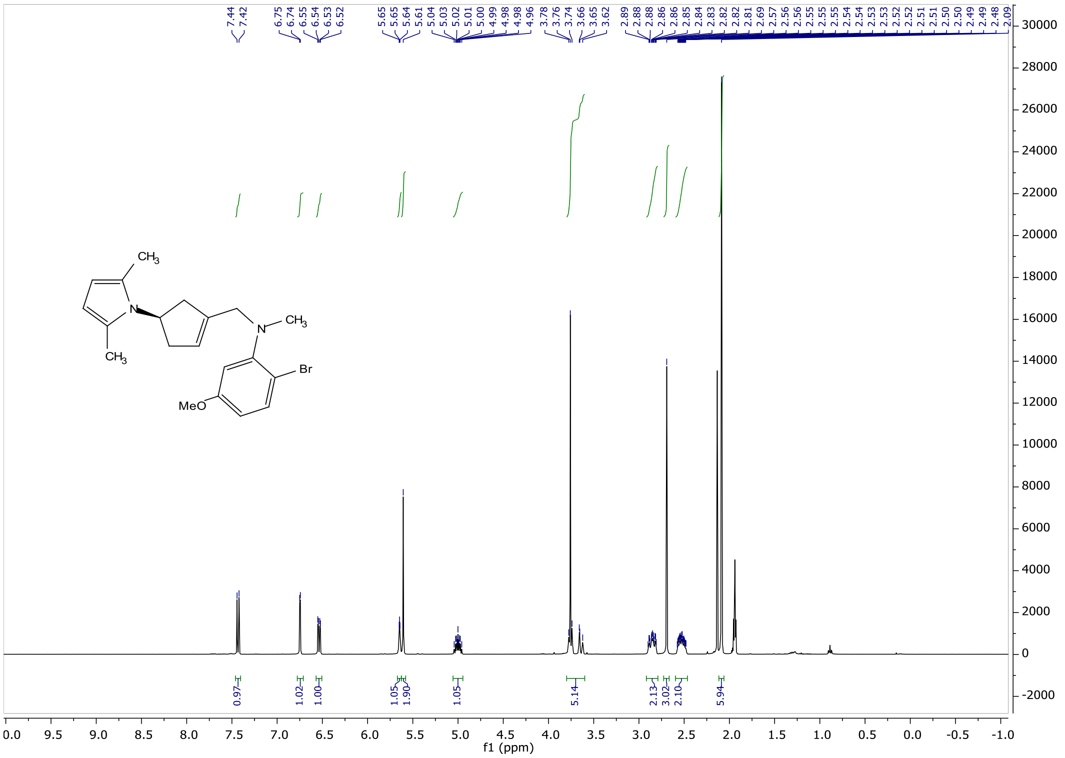

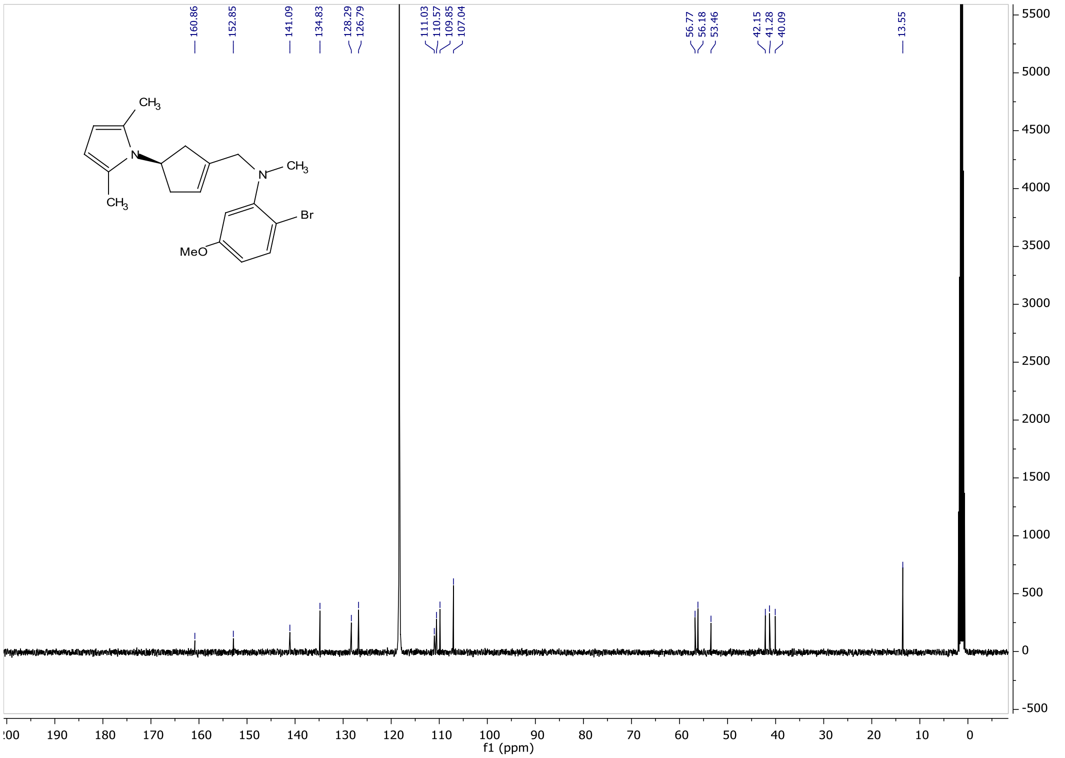

**Figure S29.** $^{1}H$ and $^{13}C\{^{1}H\}$ NMR spectra of **8l** in $CD_3CN$.

**(*R*)-3-bromo-*N*-((4-(2,5-dimethyl-1H-pyrrol-1-yl)cyclopent-1-en-1-yl)methyl)-*N*-methylpyridin-2-amine (8m)**

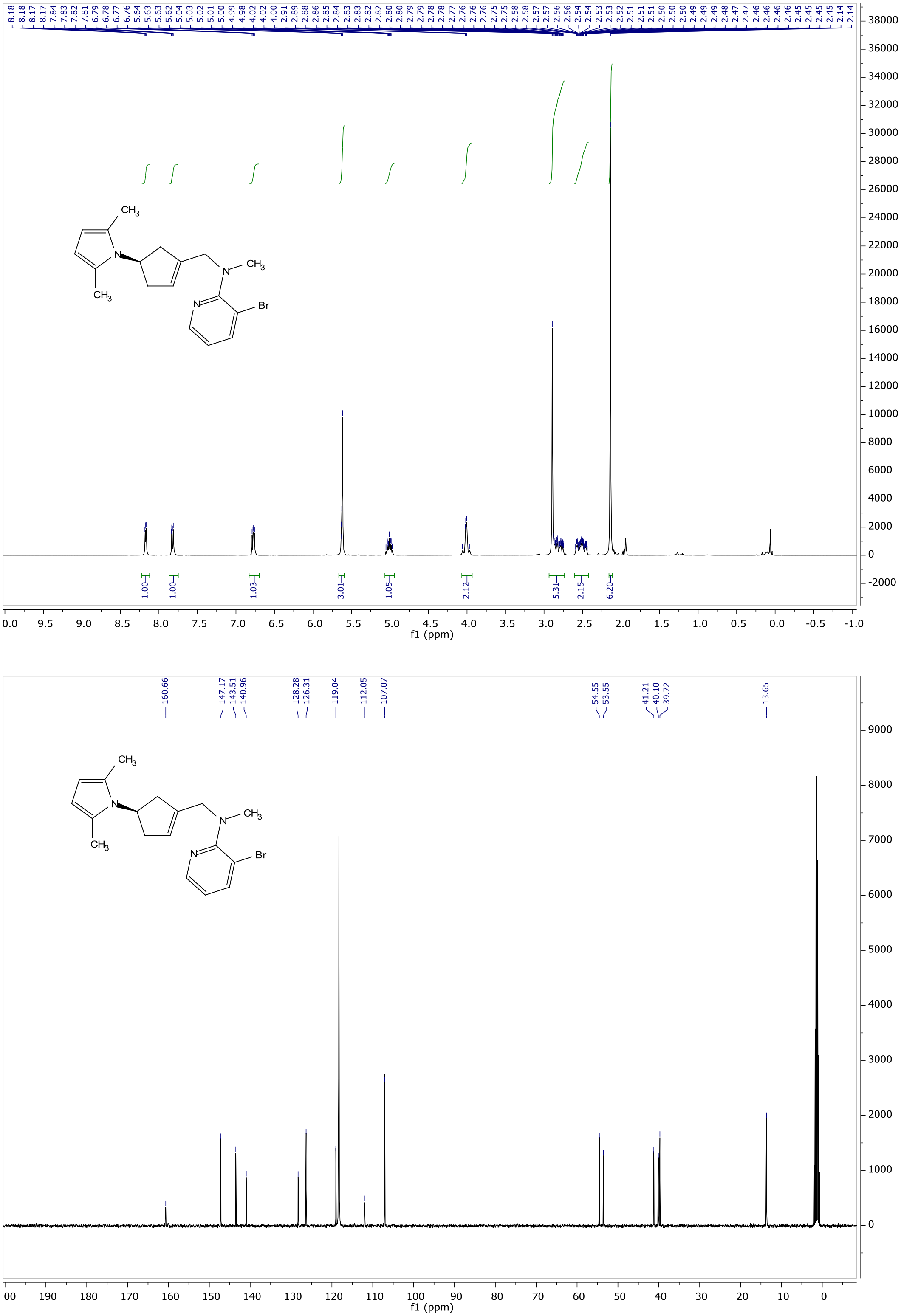


**Figure S30.** $^{1}H$ and $^{13}C\{^{1}H\}$ NMR spectra of **8m** in $CD_3CN$.

**(*S*)-2-bromo-*N*-((4-(2,5-dimethyl-1H-pyrrol-1-yl)cyclopent-1-en-1-yl)methyl)-*N*-methylaniline (8n)**

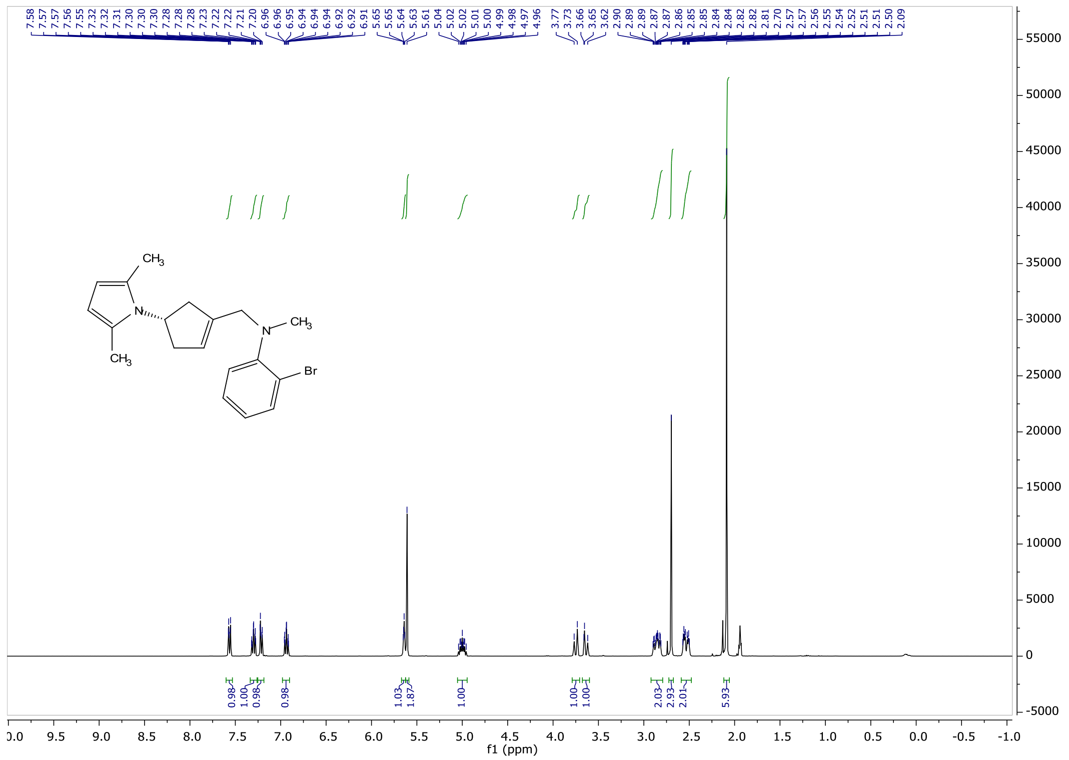


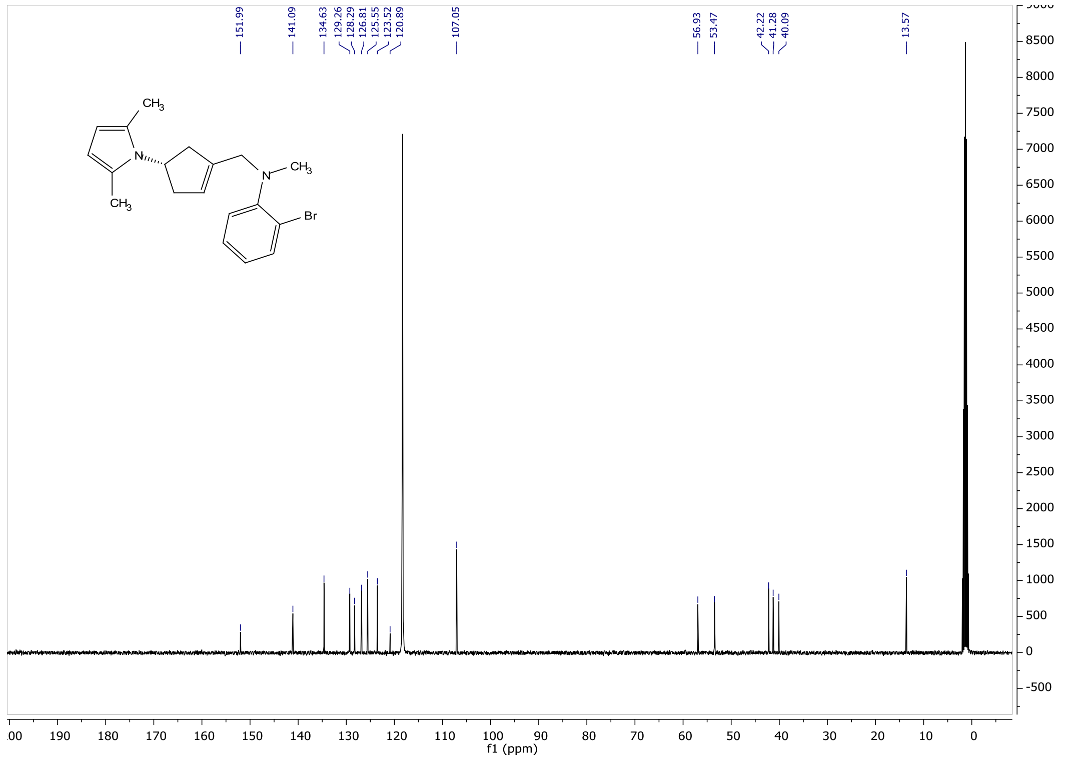


**Figure S31.** $^{1}H$ and $^{13}C\{^{1}H\}$ NMR spectra of **8n** in $CD_3CN$.

**(1*R*,4*S*)-4-(2,5-dimethyl-1H-pyrrol-1-yl)-1'-methylspiro[cyclopentane-1,3'-indolin]-2-ene (9a)**

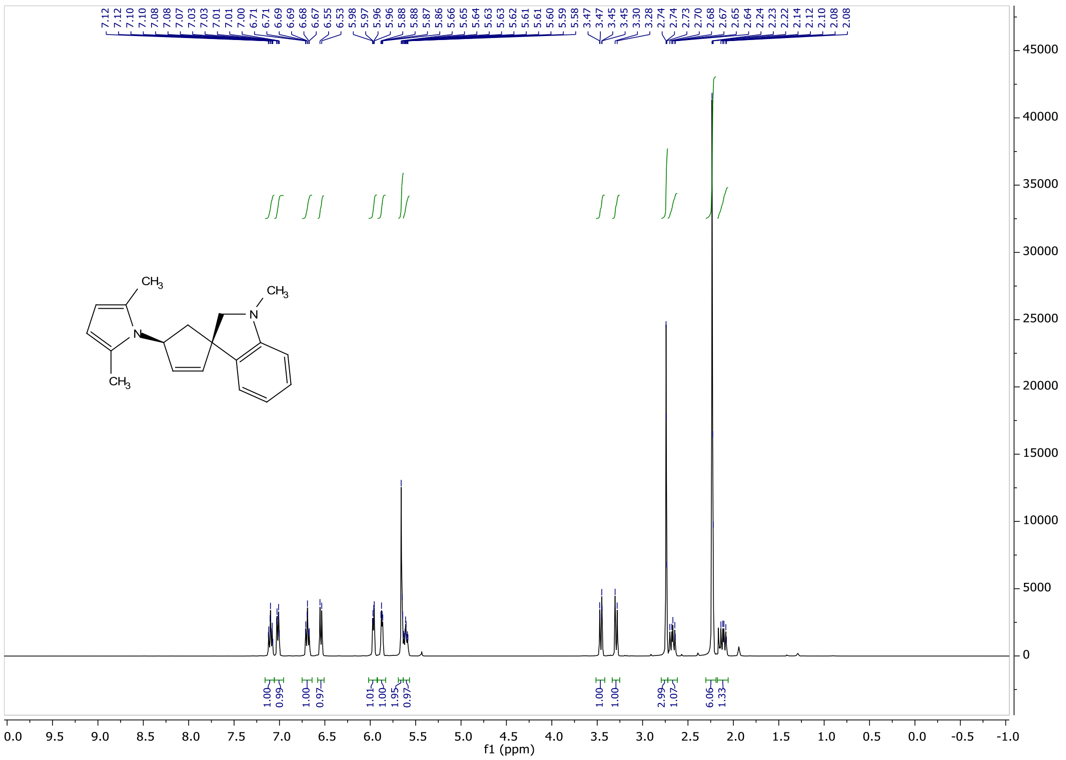


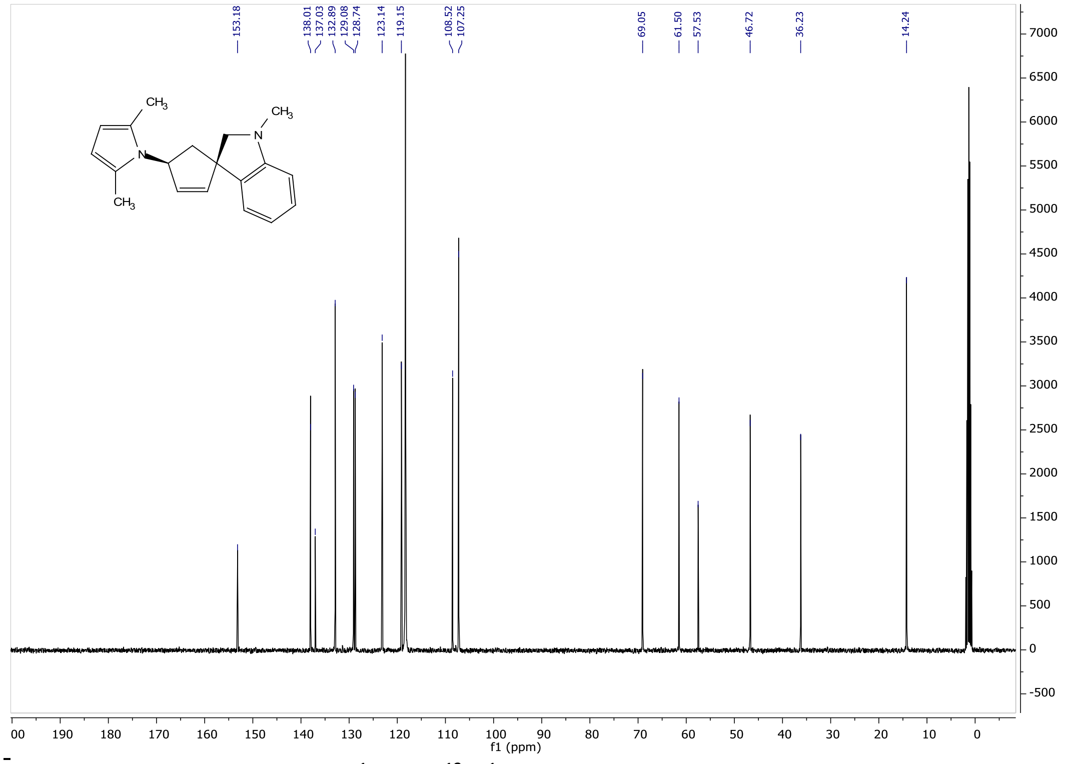


**Figure S32.** $^{1}H$ and $^{13}C\{^{1}H\}$ NMR spectra of **9a** in $CD_3CN$.

**(1*R*,4*S*)-4-(2,5-dimethyl-1H-pyrrol-1-yl)-1'-methylspiro[cyclopentane-1,3'-indolin]-2-ene-5'-carbonitrile (9b)**

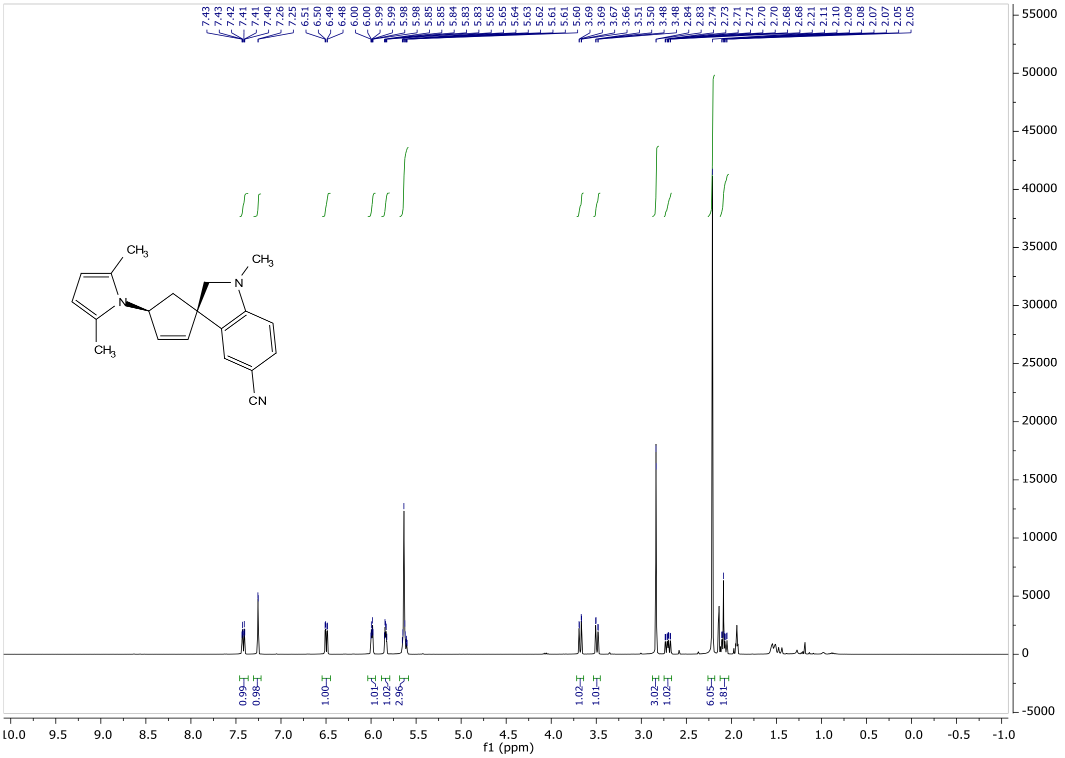


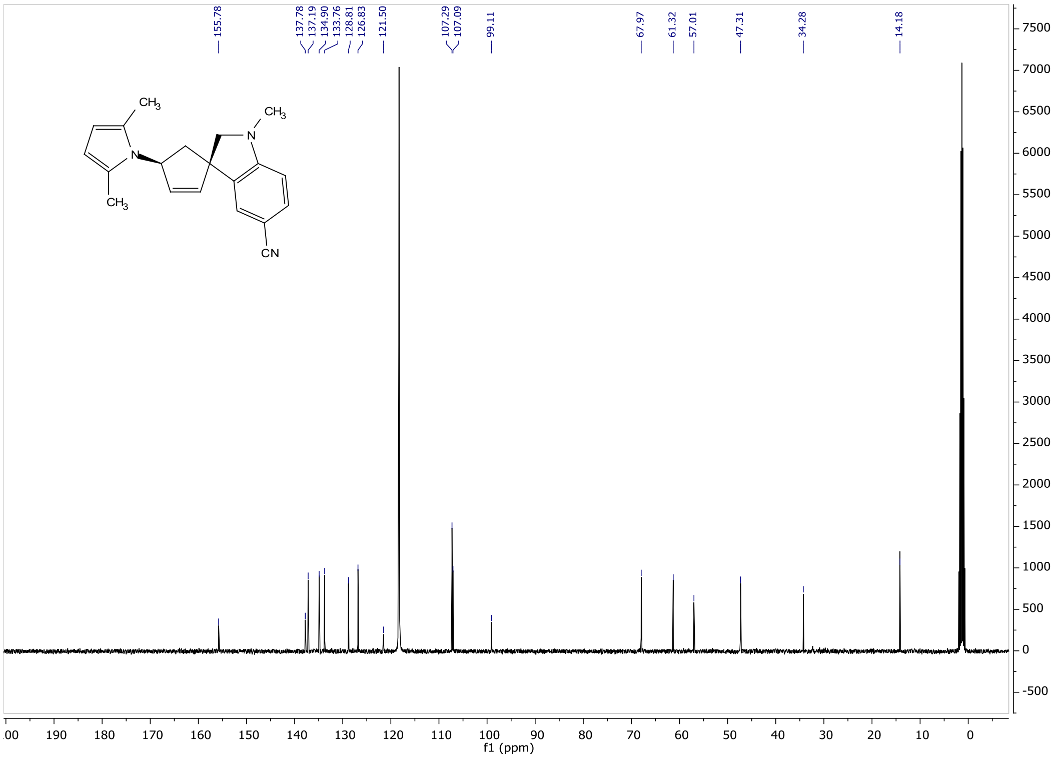


**Figure S33.** $^1$H and $^{13}$C{$^1$H} NMR spectra of **9b** in $CD_3CN$.

**(1*R*,4*S*)-5'-chloro-4-(2,5-dimethyl-1H-pyrrol-1-yl)-1'-methylspiro[cyclopentane-1,3'-indolin]-2-ene (9c)**

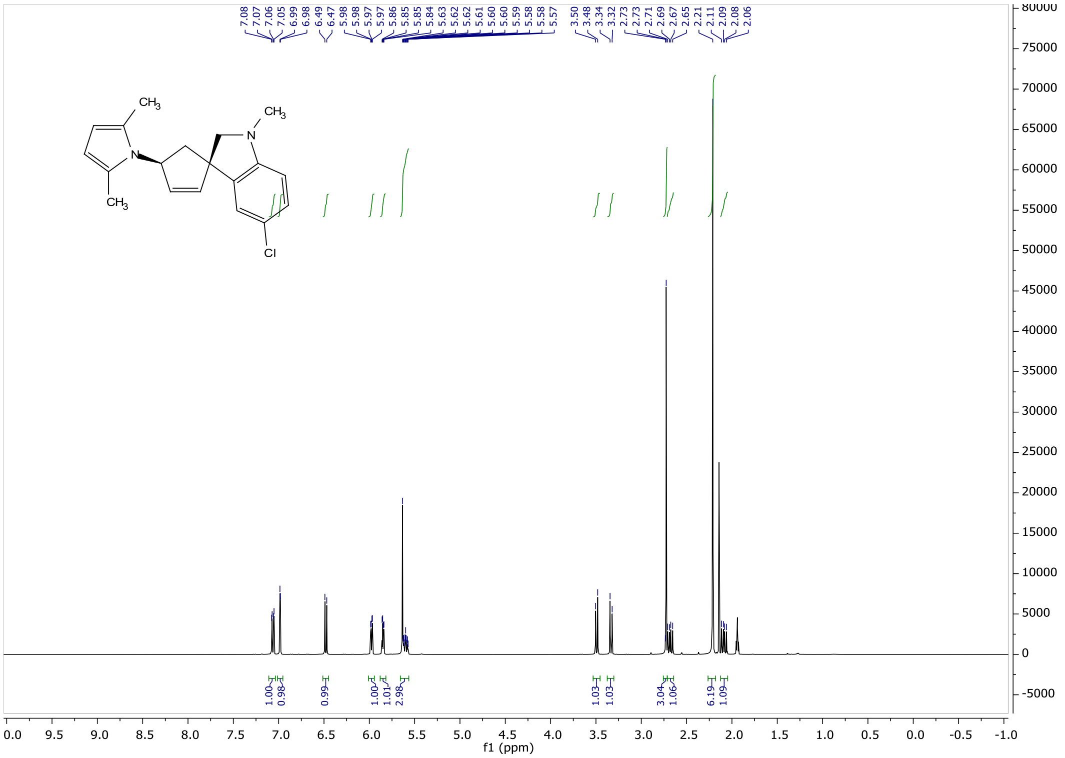

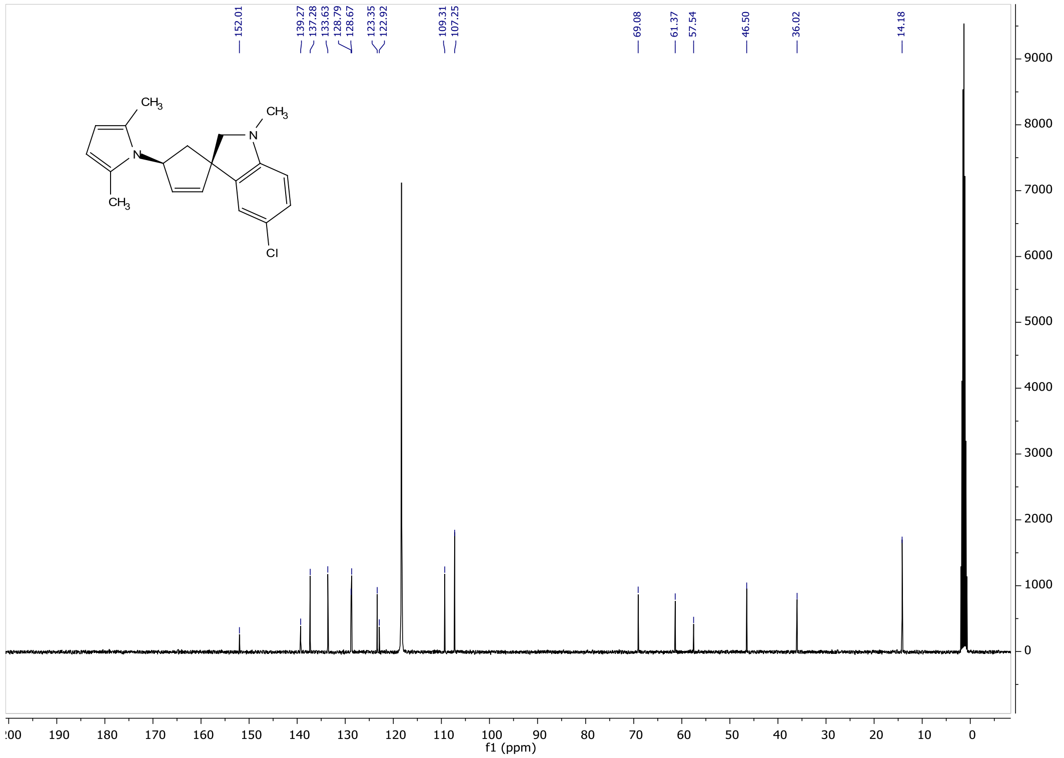

**Figure S34.** $^1H$ and $^{13}C\{^1H\}$ NMR spectra of **9c** in $CD_3CN$.

**(1*R*,4*S*)-4-(2,5-dimethyl-1H-pyrrol-1-yl)-1'-methyl-4'-(trifluoromethyl)spiro[cyclopentane-1,3'-indolin]-2-ene (9d)**

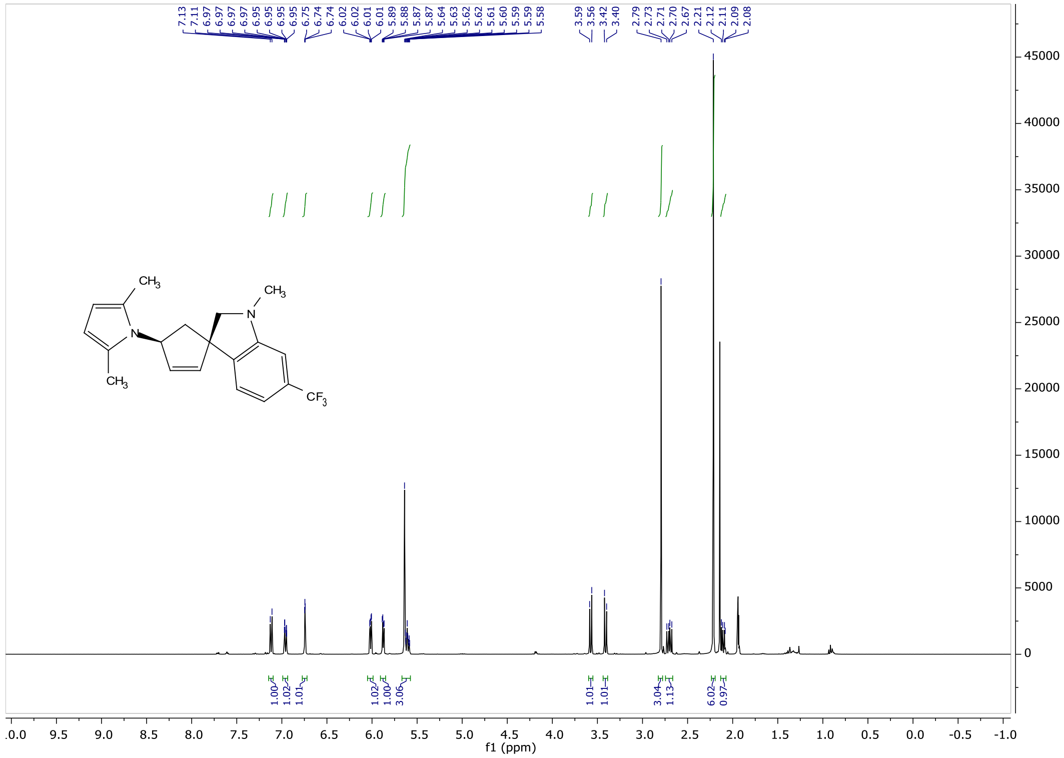

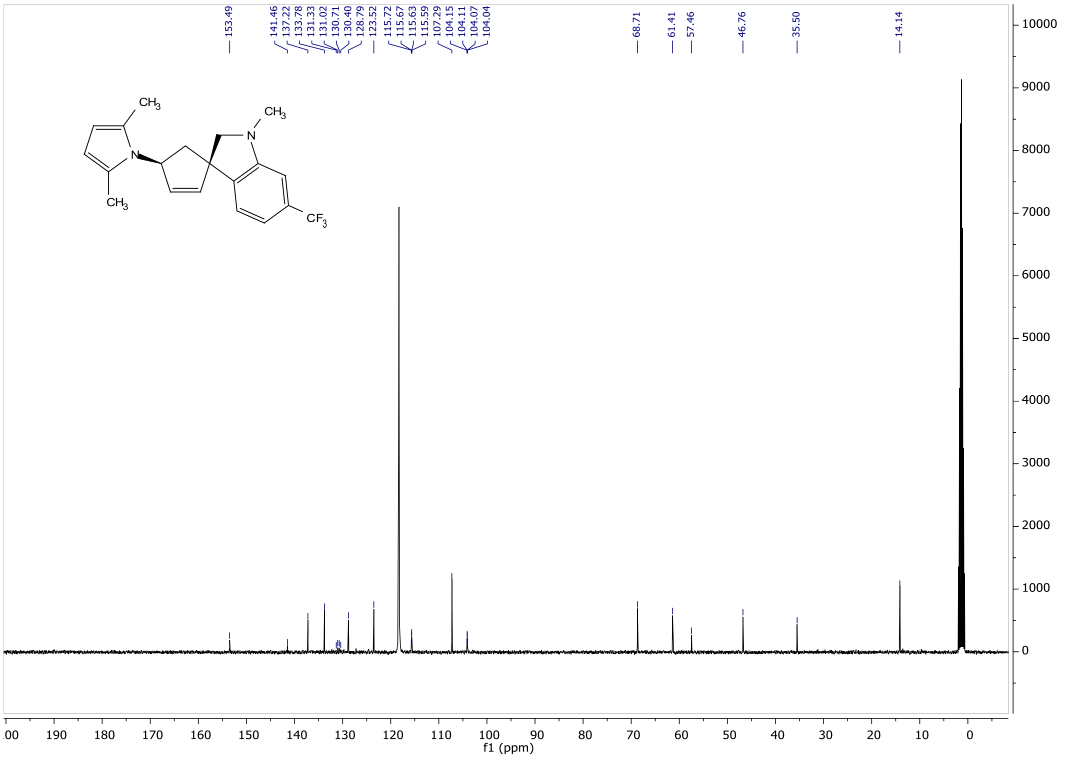

**Figure S35.** $^{1}H$ and $^{13}C\{^{1}H\}$ NMR spectra of **9d** in $CD_3CN$.

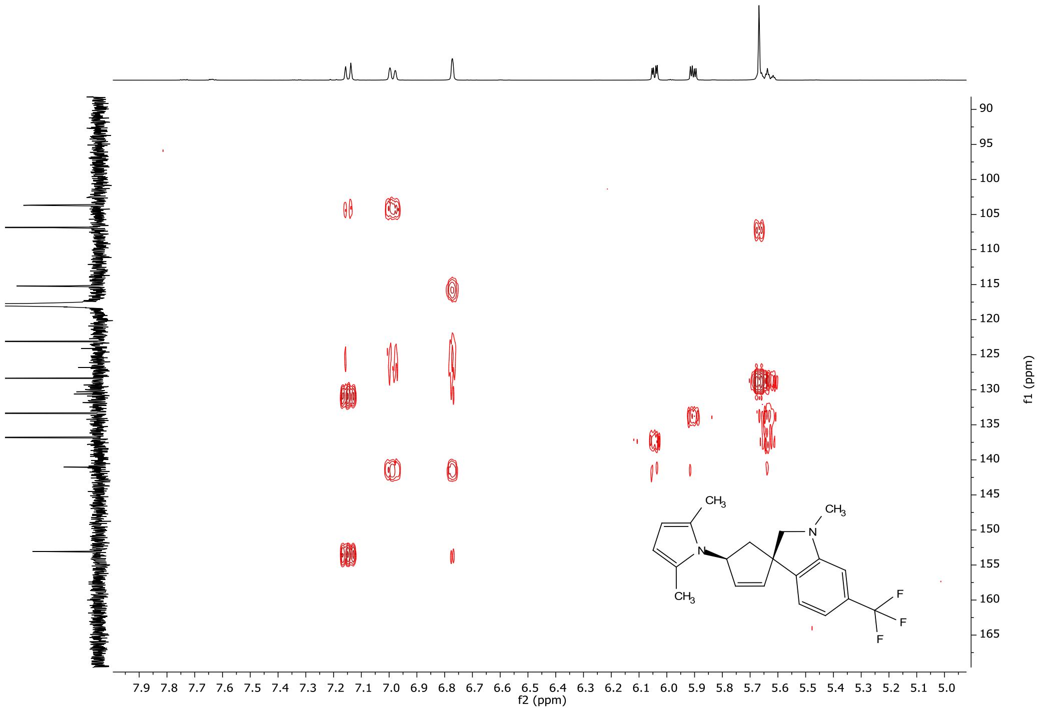


**Figure S36.** HMBC spectrum of **9d** indicating shift of the missing ipso carbon ~125.5 ppm.

**(1*R*,4*S*)-4-(2,5-dimethyl-1H-pyrrol-1-yl)-1',4'-dimethylspiro[cyclopentane-1,3'-indolin]-2-ene** (9e)

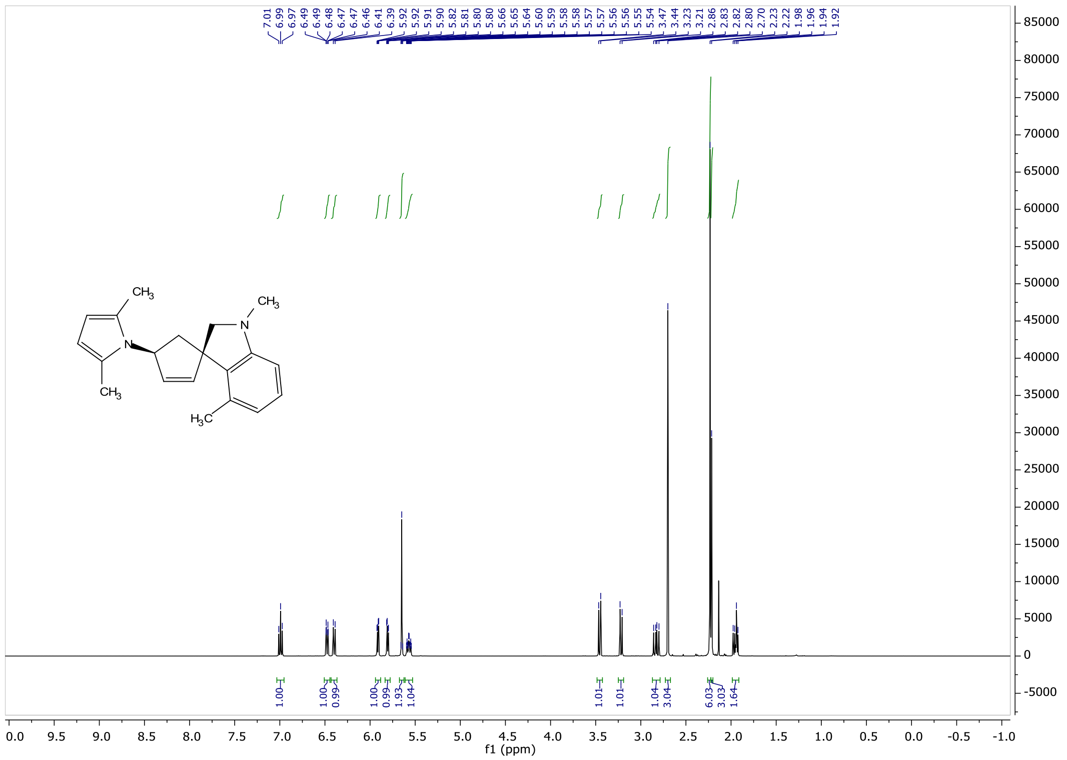


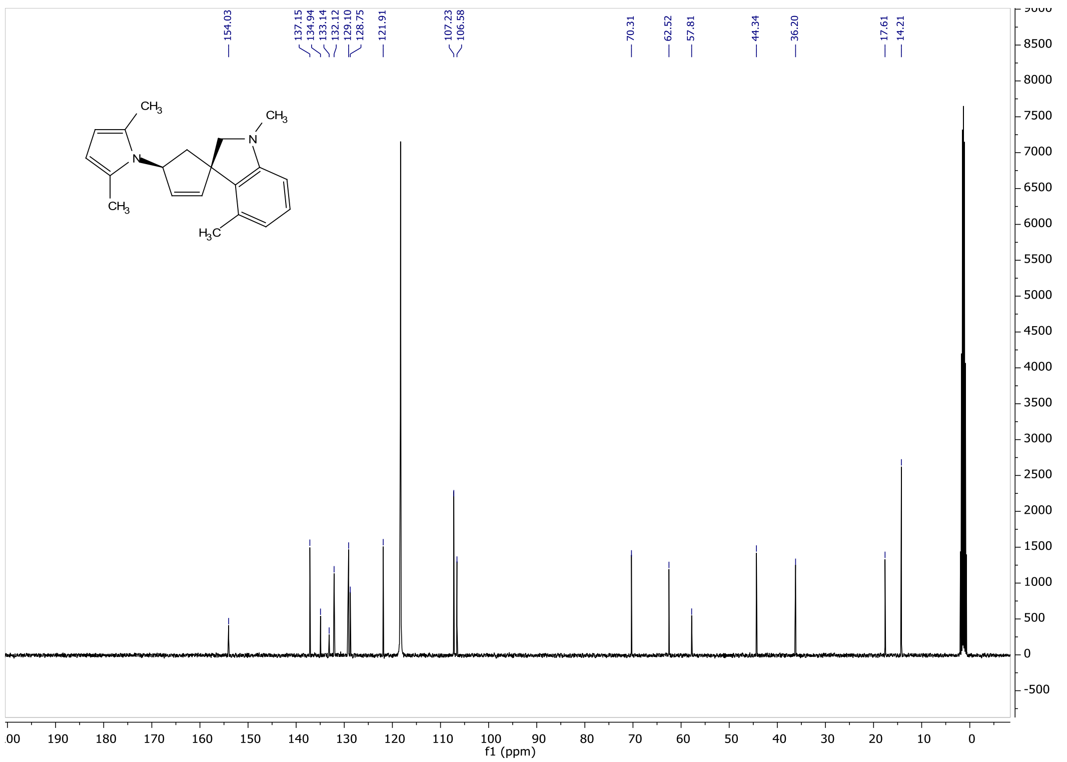


**Figure S37.** $^{1}H$ and $^{13}C\{^{1}H\}$ NMR spectra of **9e** in $CD_3CN$.

**(1*R*,4*S*)-4-(2,5-dimethyl-1H-pyrrol-1-yl)-1',5'-dimethylspiro[cyclopentane-1,3'-indolin]-2-ene** (**9f**)

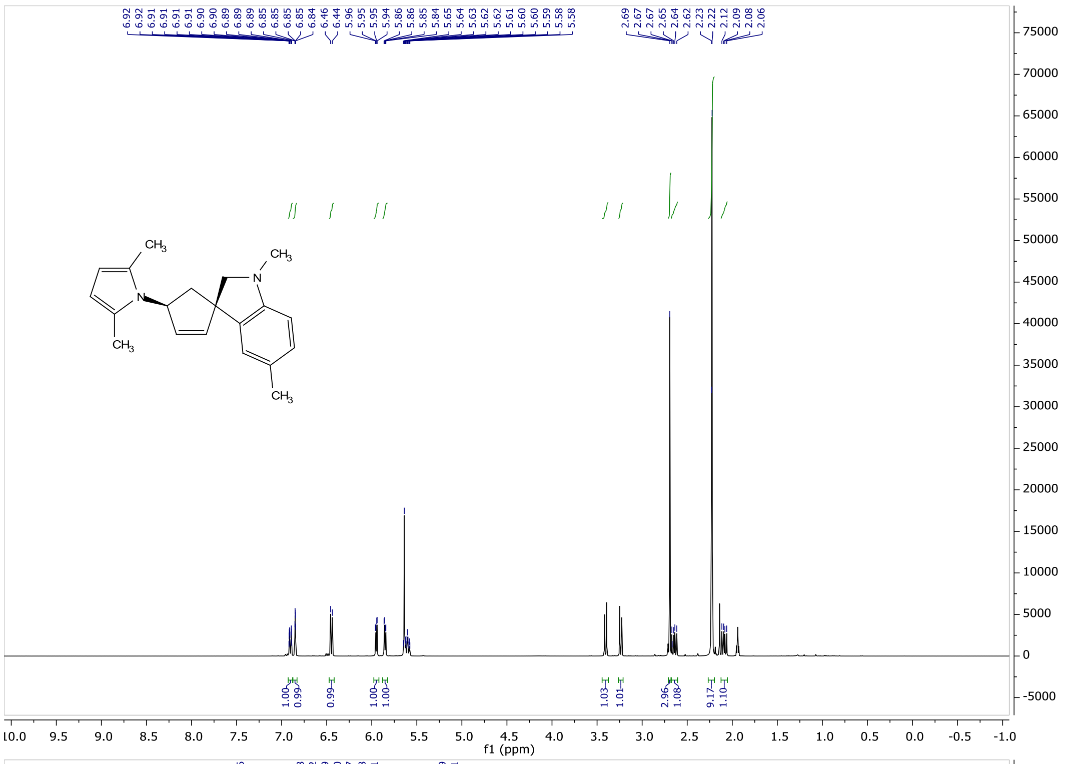


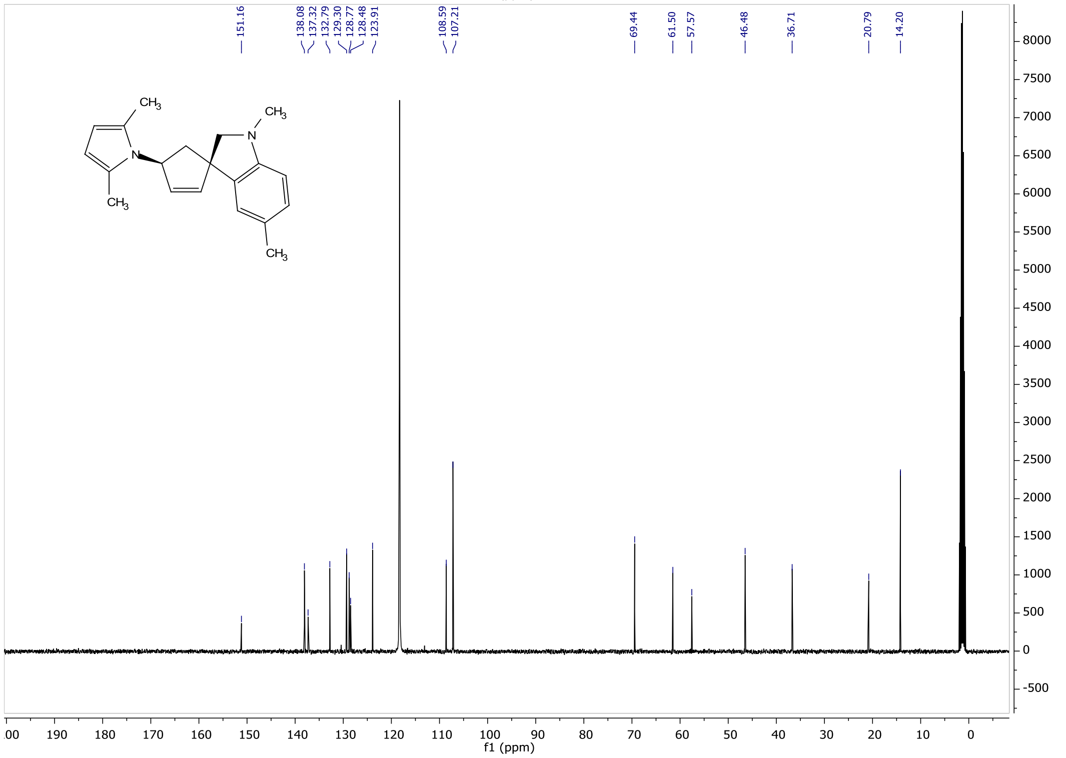


**Figure S38.** $^{1}H$ and $^{13}C\{^{1}H\}$ NMR spectra of **9f** in $CD_3CN$.

**(1*R*,4*S*)-4-(2,5-dimethyl-1H-pyrrol-1-yl)-1',6'-dimethylspiro[cyclopentane-1,3'-indolin]-2-ene (9g)**

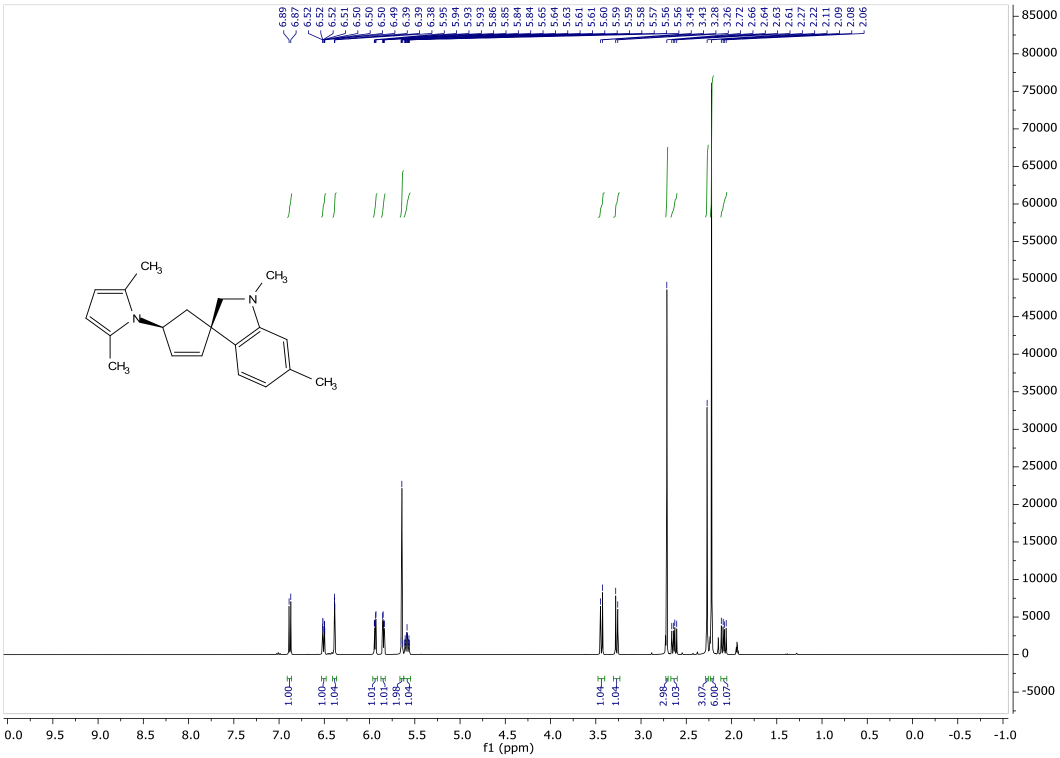


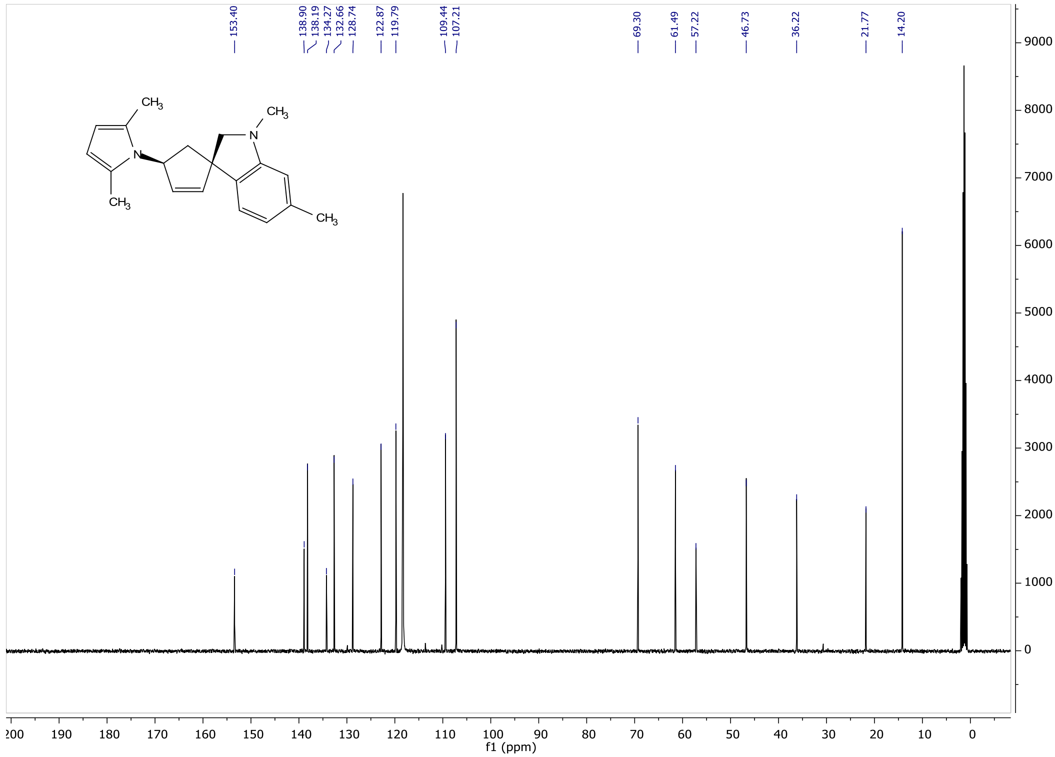


**Figure S39.** $^{1}H$ and $^{13}C\{^{1}H\}$ NMR spectra of **9g** in $CD_3CN$.

**(1*R*,4*S*)-4-(2,5-dimethyl-1H-pyrrol-1-yl)-1',7'-dimethylspiro[cyclopentane-1,3'-indolin]-2-ene (9h)**

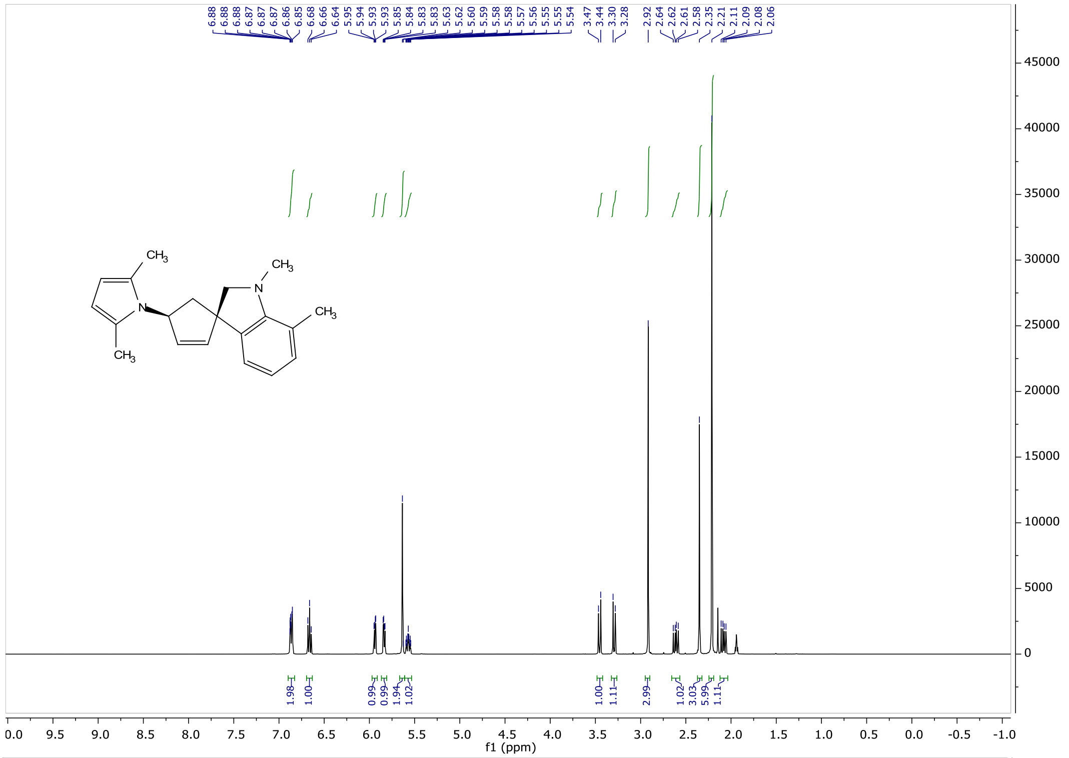


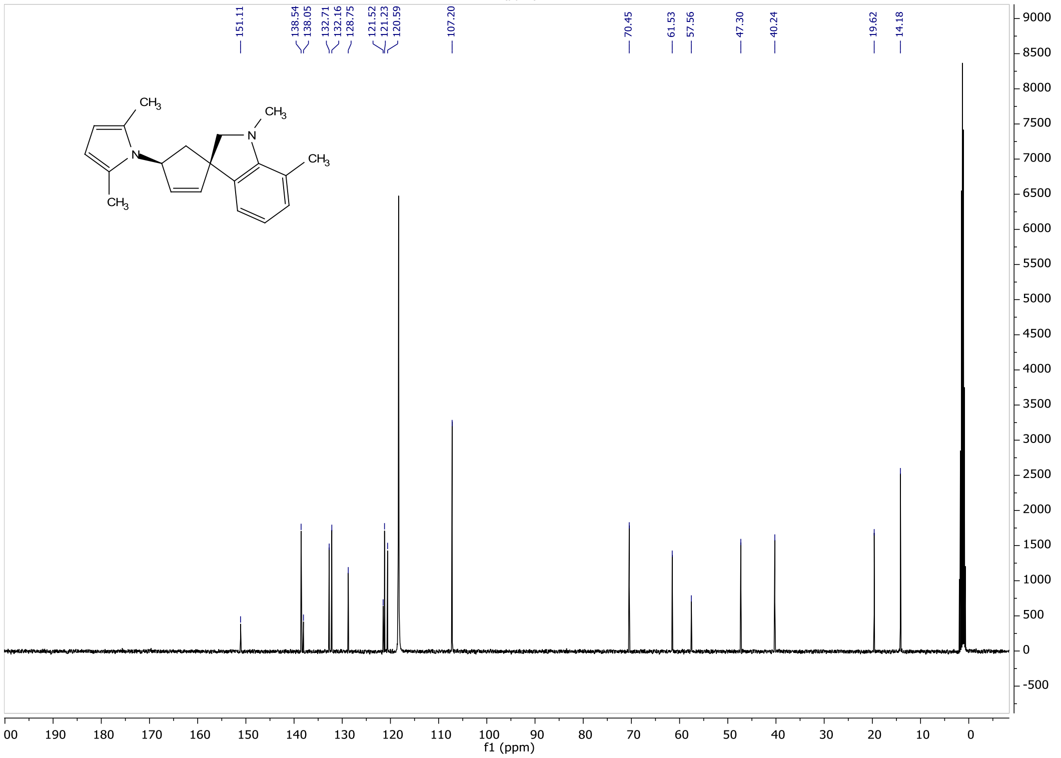


**Figure S40.** $^{1}H$ and $^{13}C\{^{1}H\}$ NMR spectra of **9h** in $CD_3CN$.

**(1*R*,4*S*)-4-(2,5-dimethyl-1H-pyrrol-1-yl)-5'-fluoro-1'-methylspiro[cyclopentane-1,3'-indolin]-2-ene (9i)**

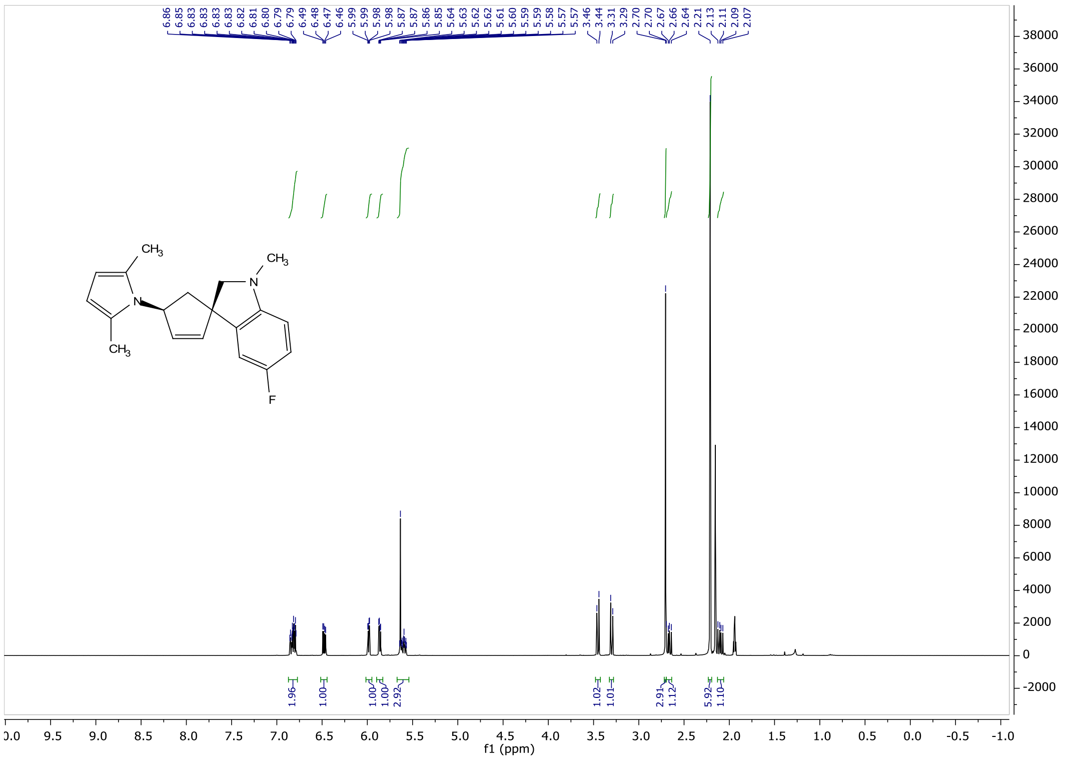


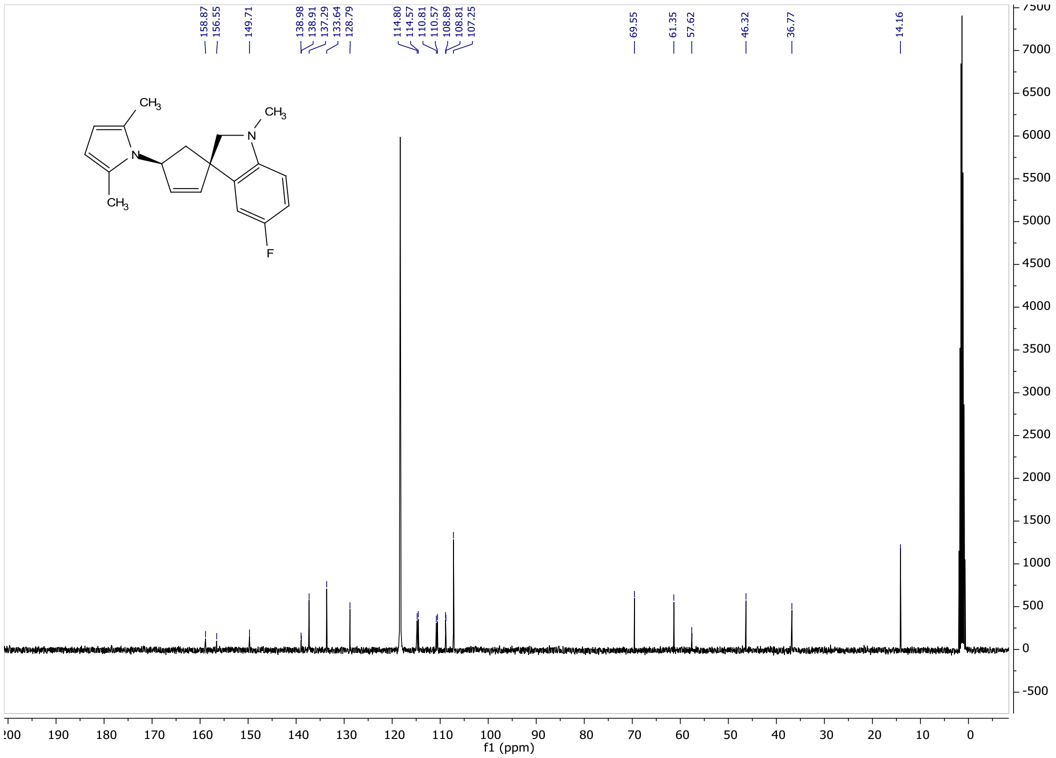


**Figure S41.** $^{1}H$ and $^{13}C\{^{1}H\}$ NMR spectra of **9i** in $CD_3CN$.

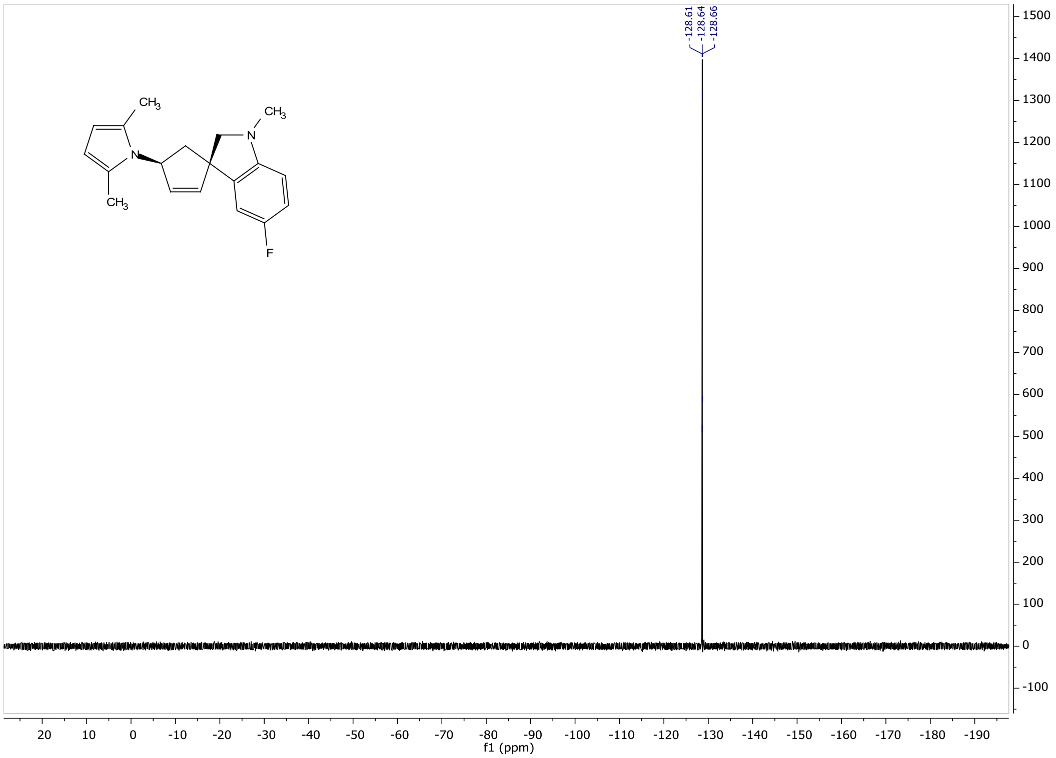


**Figure S42.** $^{19}$F NMR spectra of **9i** in $CD_3CN$.

## (1*R*,4*S*)-5'-bromo-4-(2,5-dimethyl-1H-pyrrol-1-yl)-1'-methylspiro[cyclopentane-1,3'-indolin]-2-ene (9j)

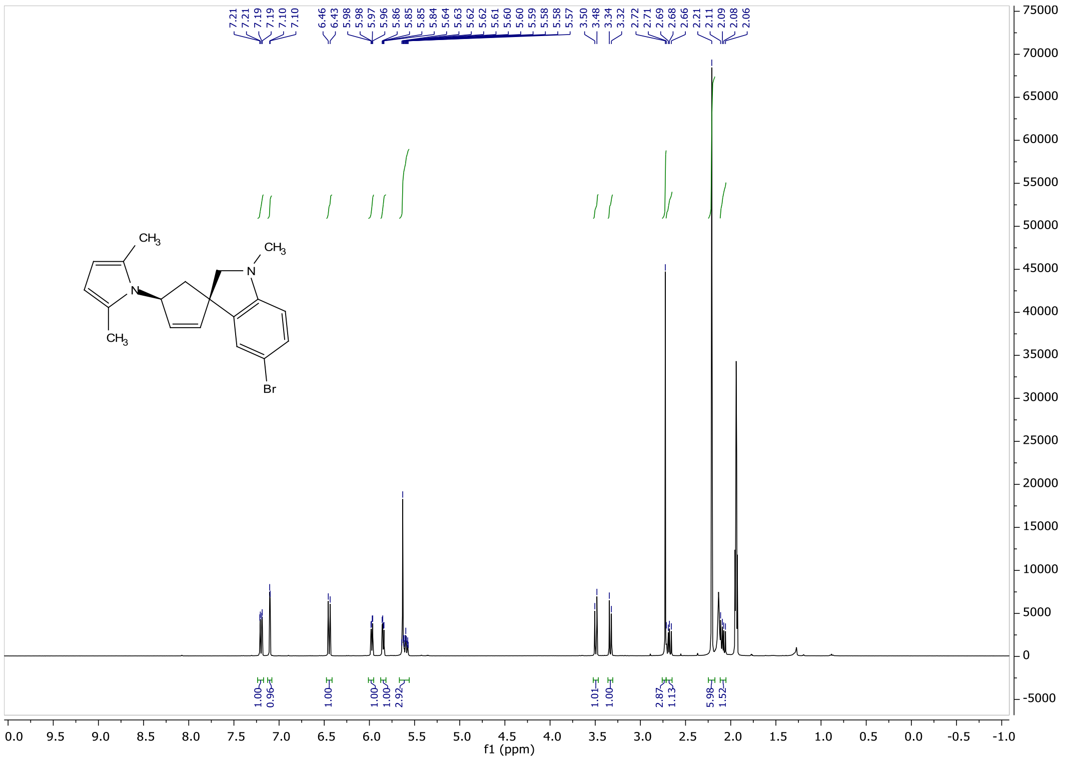


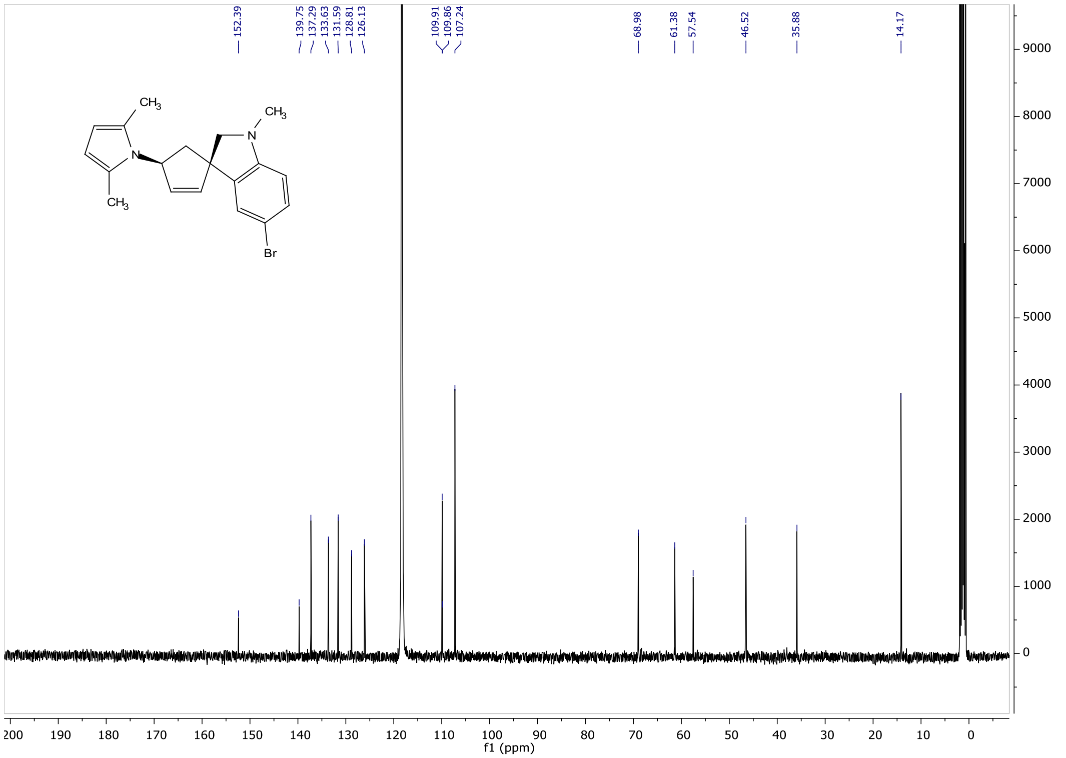


**Figure S43.** $^{1}H$ and $^{13}C\{^{1}H\}$ NMR spectra of **9j** in $CD_3CN$.

**(1*R*,4*S*)-6'-bromo-4-(2,5-dimethyl-1H-pyrrol-1-yl)-1'-methylspiro[cyclopentane-1,3'-indolin]-2-ene (9k)**

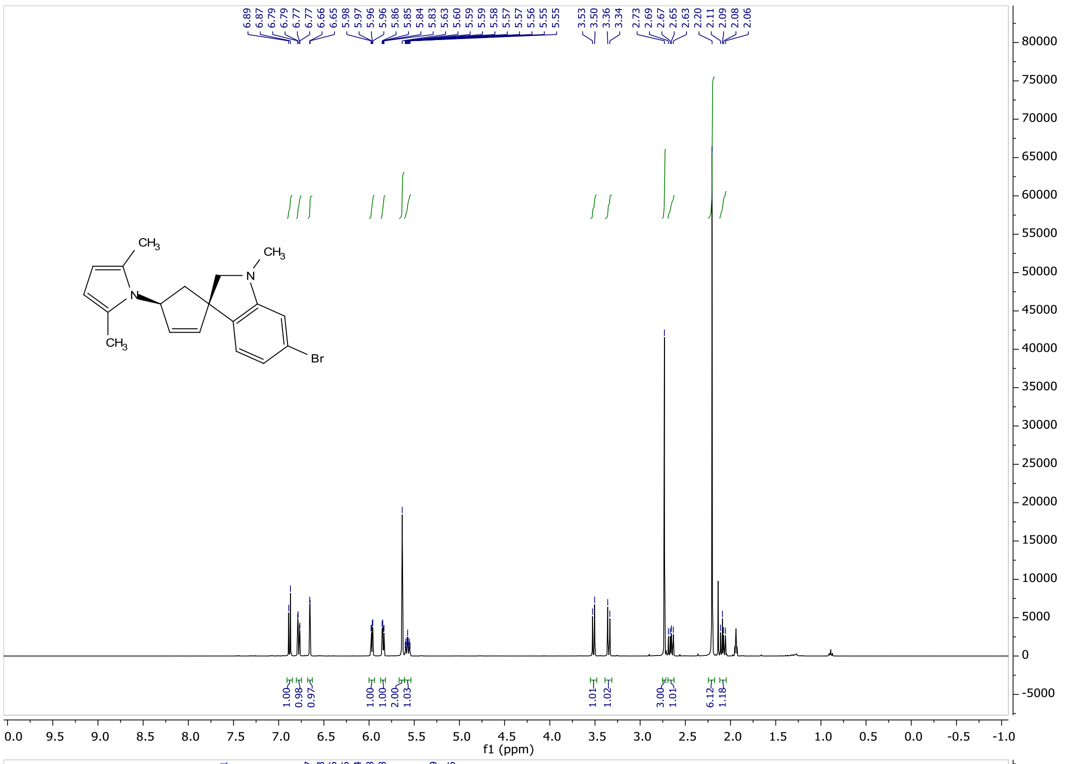


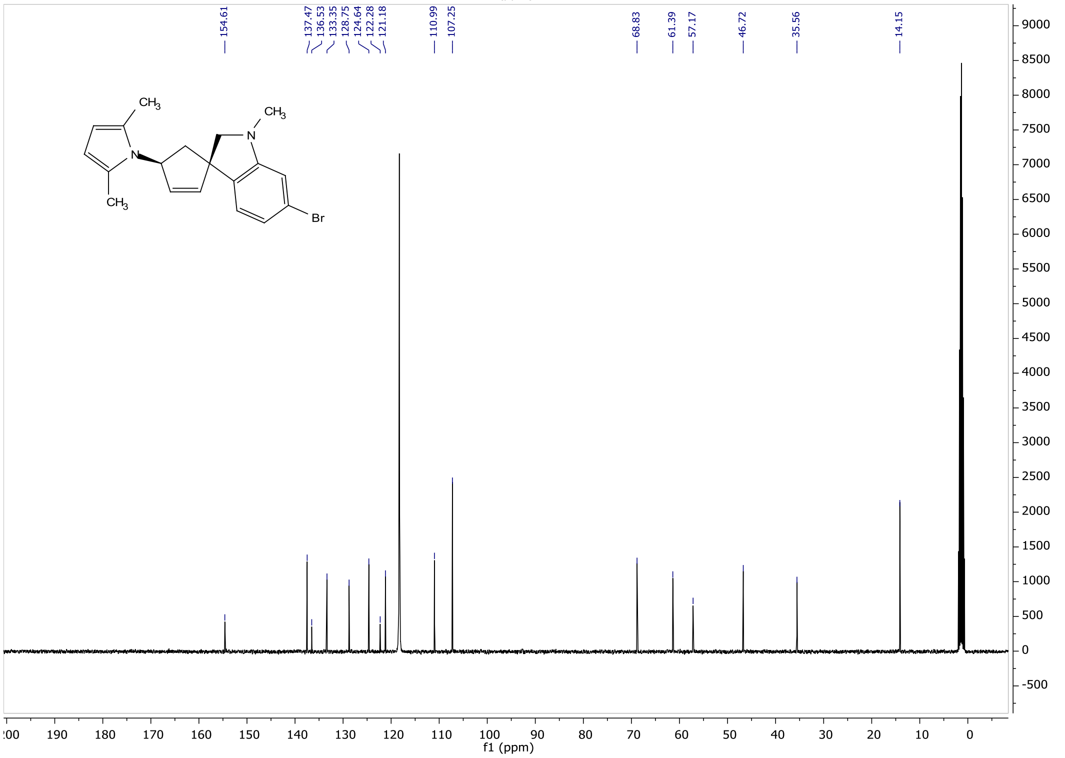


**Figure S44.** $^{1}H$ and $^{13}C\{^{1}H\}$ NMR spectra of **9k** in $CD_3CN$.

**(1*R*,4*S*)-4-(2,5-dimethyl-1H-pyrrol-1-yl)-6'-methoxy-1'-methylspiro[cyclopentane-1,3'-indolin]-2-ene (9l)**

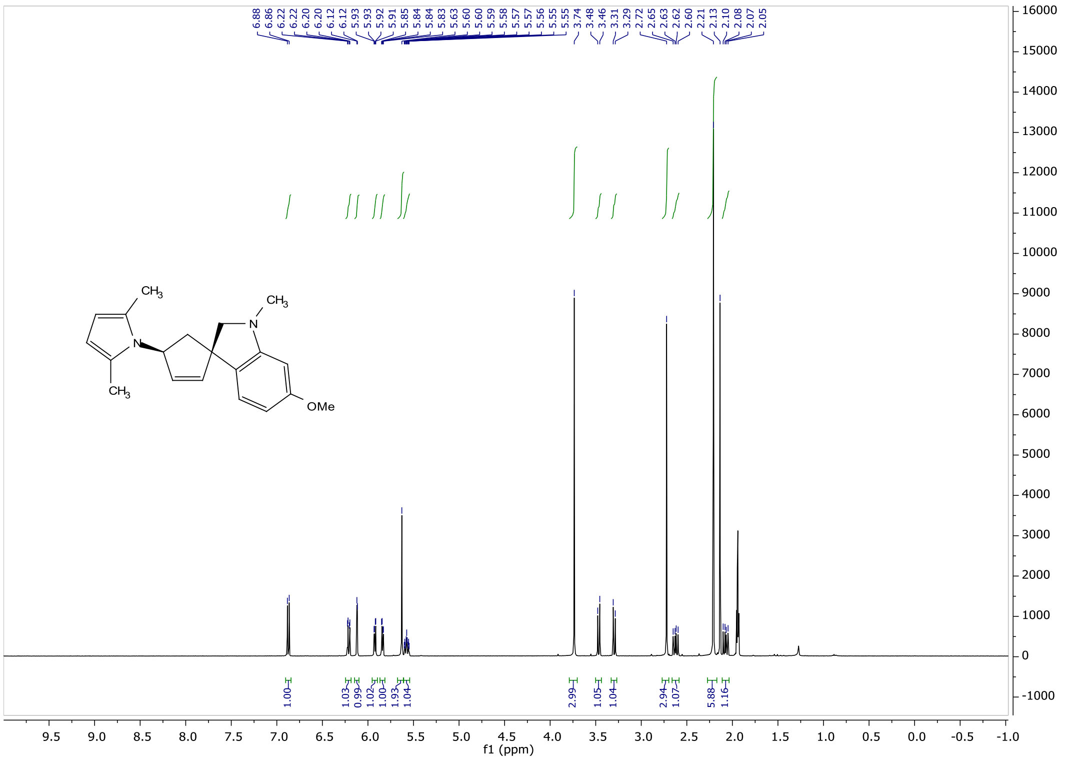

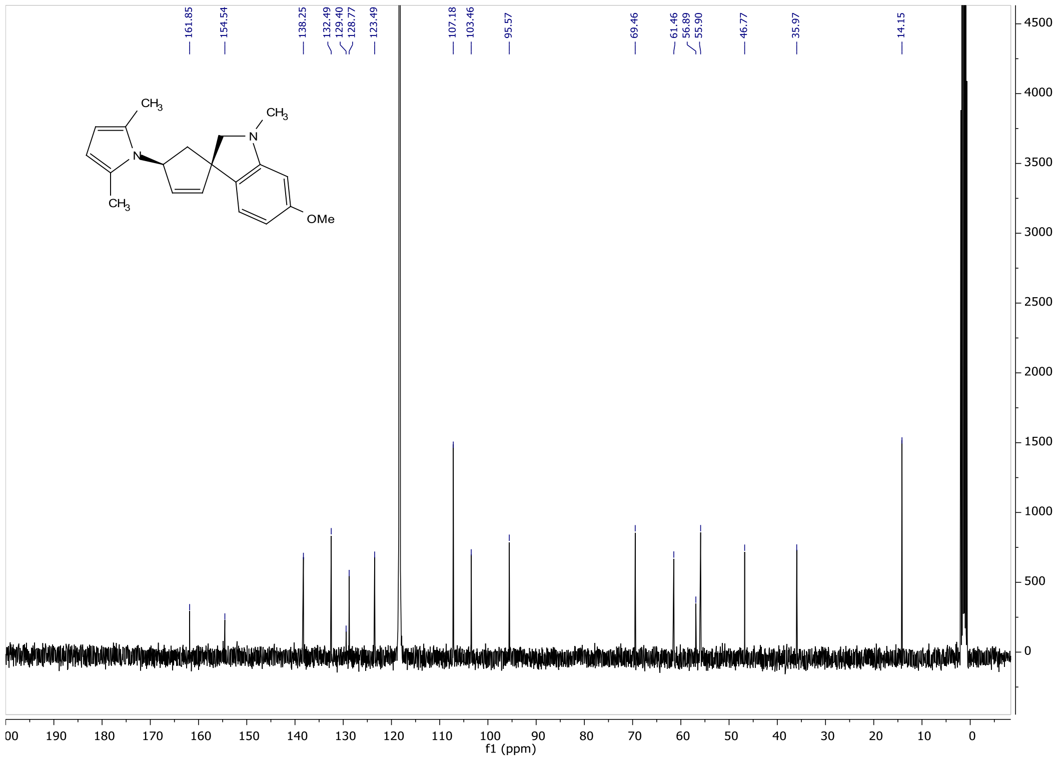

**Figure S45.** $^{1}H$ and $^{13}C\{^{1}H\}$ NMR spectra of **9l** in $CD_3CN$.

**(1*R*,4*S*)-4-(2,5-dimethyl-1H-pyrrol-1-yl)-1'-methyl-1',2'-dihydrospiro[cyclopentane-1,3'-pyrrolo[2,3-b]pyridin]-2-ene** (**9m**)

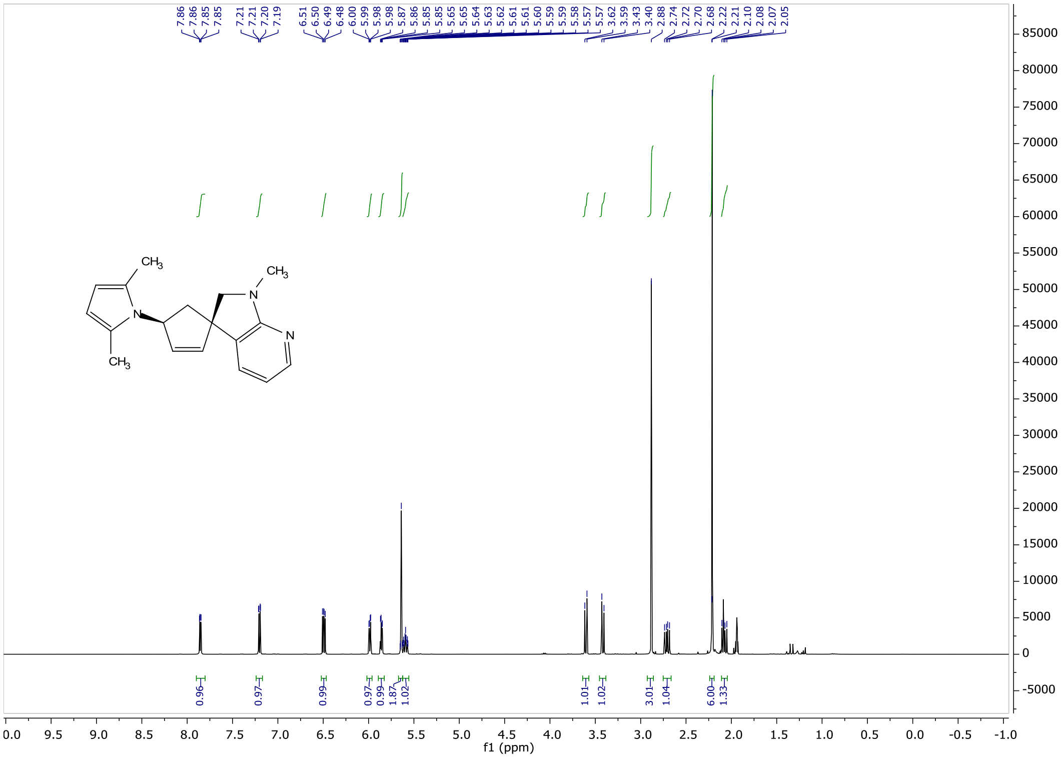


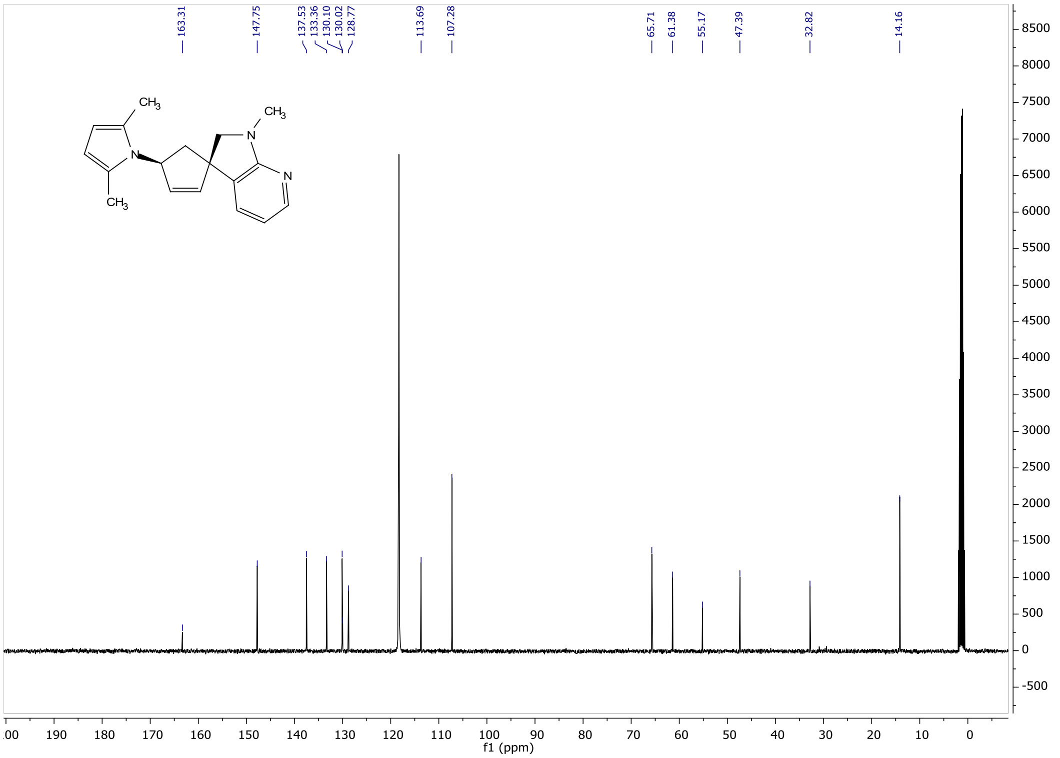


**Figure S46.** $^{1}H$ and $^{13}C\{^{1}H\}$ NMR spectra of **9m** in $CD_3CN$.

**(1*S*,4*R*)-4-(2,5-dimethyl-1H-pyrrol-1-yl)-1'-methylspiro[cyclopentane-1,3'-indolin]-2-ene** (9n)

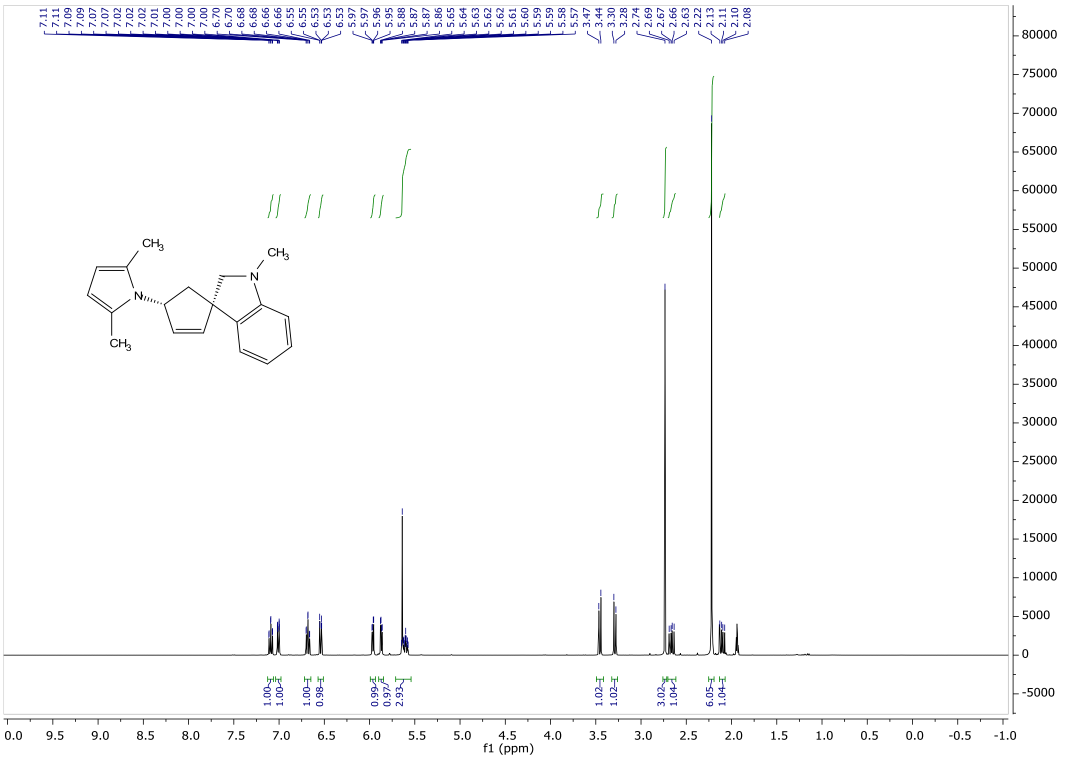

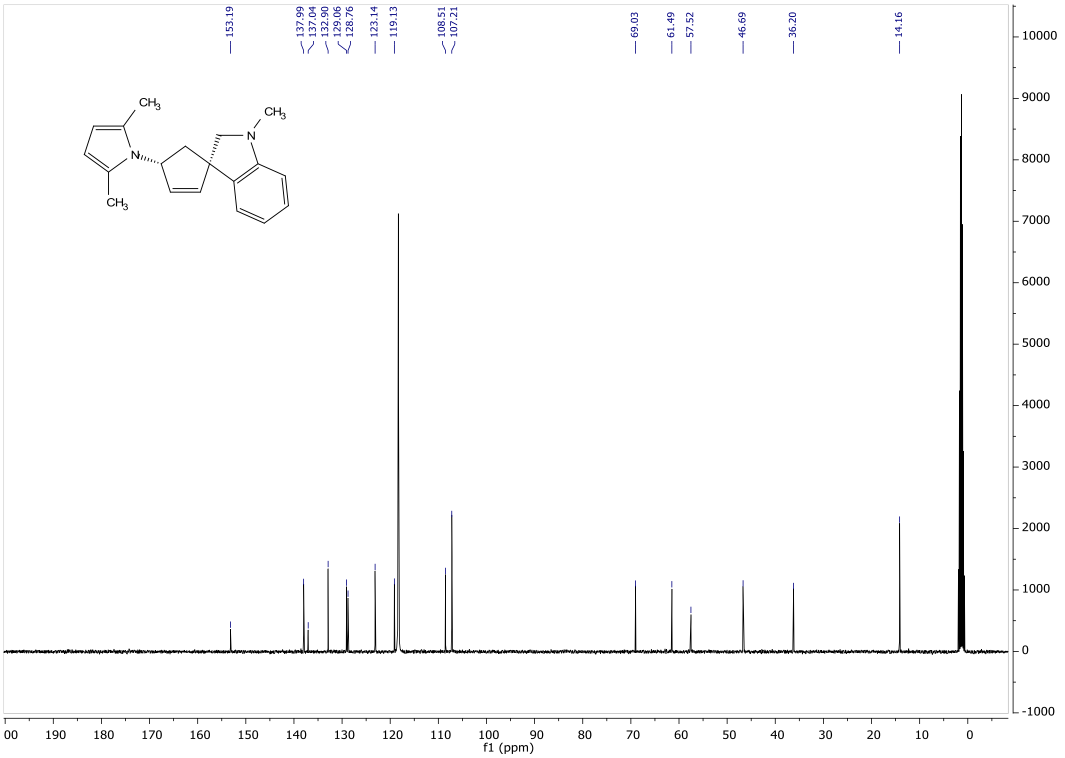

**Figure S47.** $^{1}H$ and $^{13}C\{^{1}H\}$ NMR spectra of **9n** in $CD_3CN$.

**Benzyl (1*R*,4*S*)-4-(2,5-dimethyl-1H-pyrrol-1-yl)-1'-methylspiro[cyclopentane-1,3'-indolin]-2-ene-6'-carboxylate (10)**

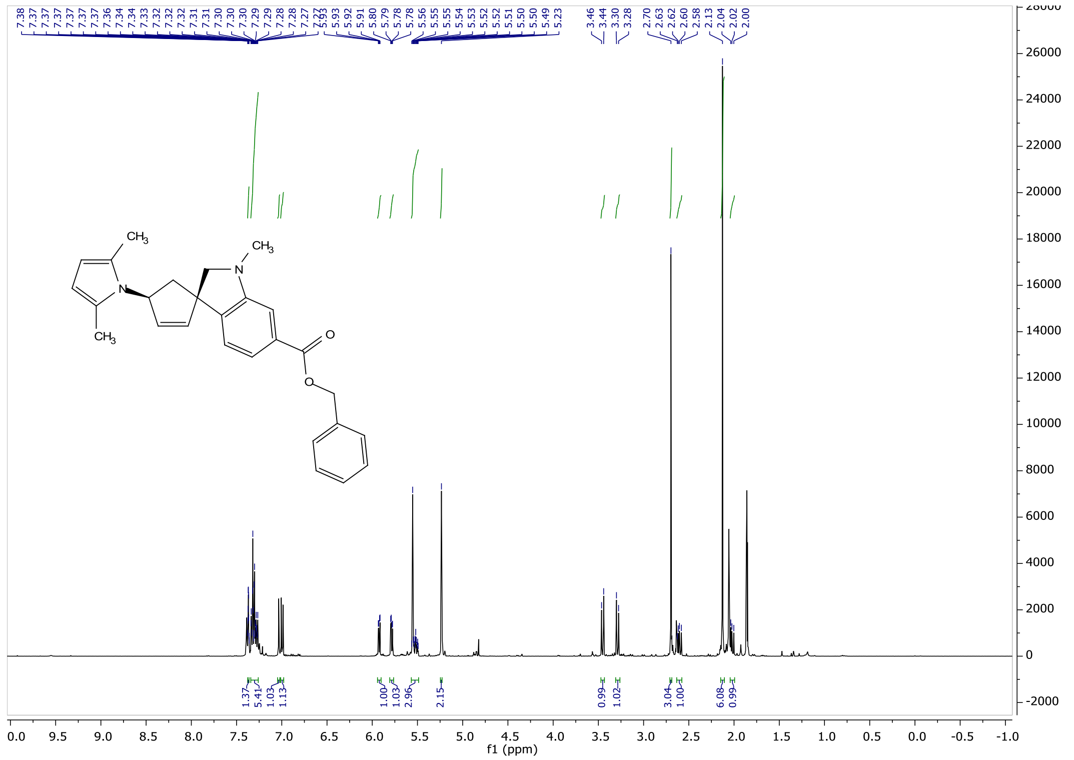

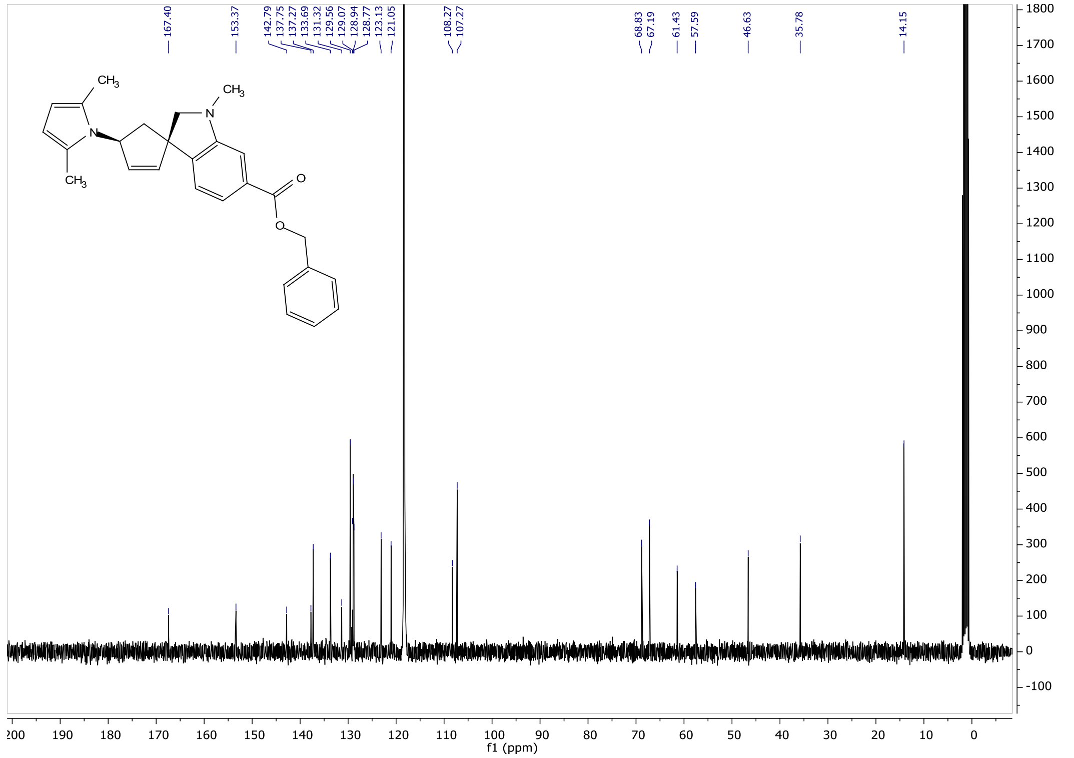

**Figure S48.** $^{1}H$ and $^{13}C\{^{1}H\}$ NMR spectra of **10** in $CD_3CN$.

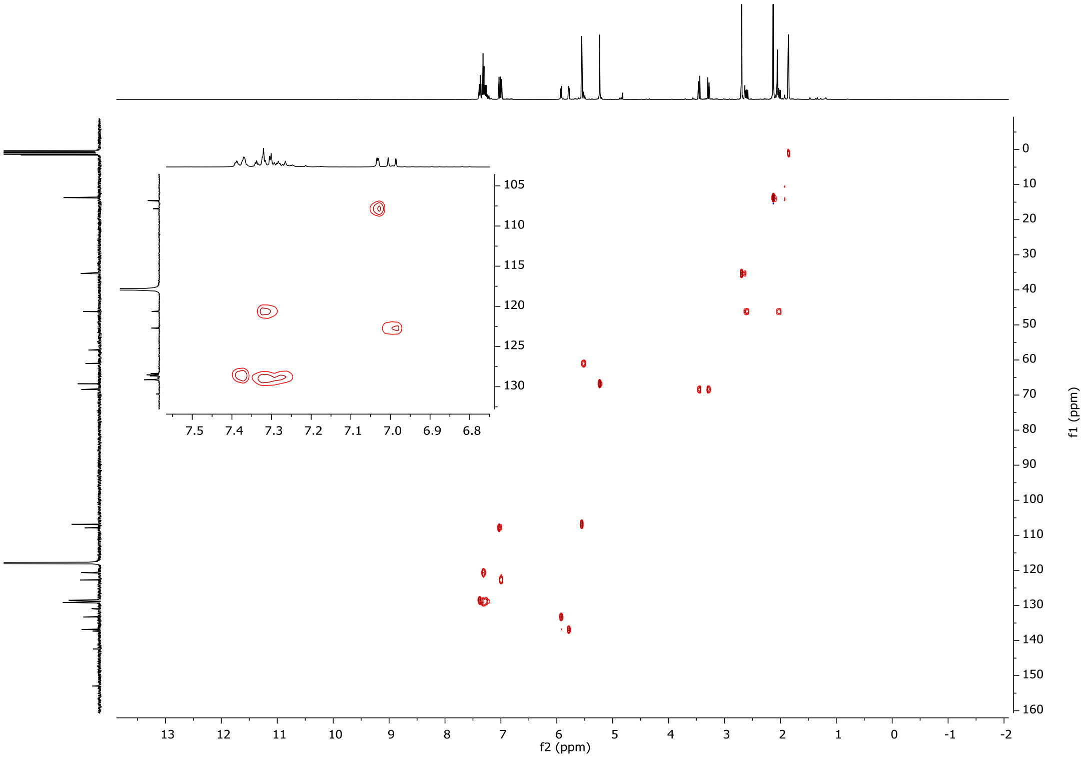


**Figure S49.** HSQC spectrum of **10** in $CD_3CN$.

**Benzyl (1*R*,4*S*)-4-amino-1'-methylspiro[cyclopentane-1,3'-indolin]-2-ene-6'-carboxylate (11)**

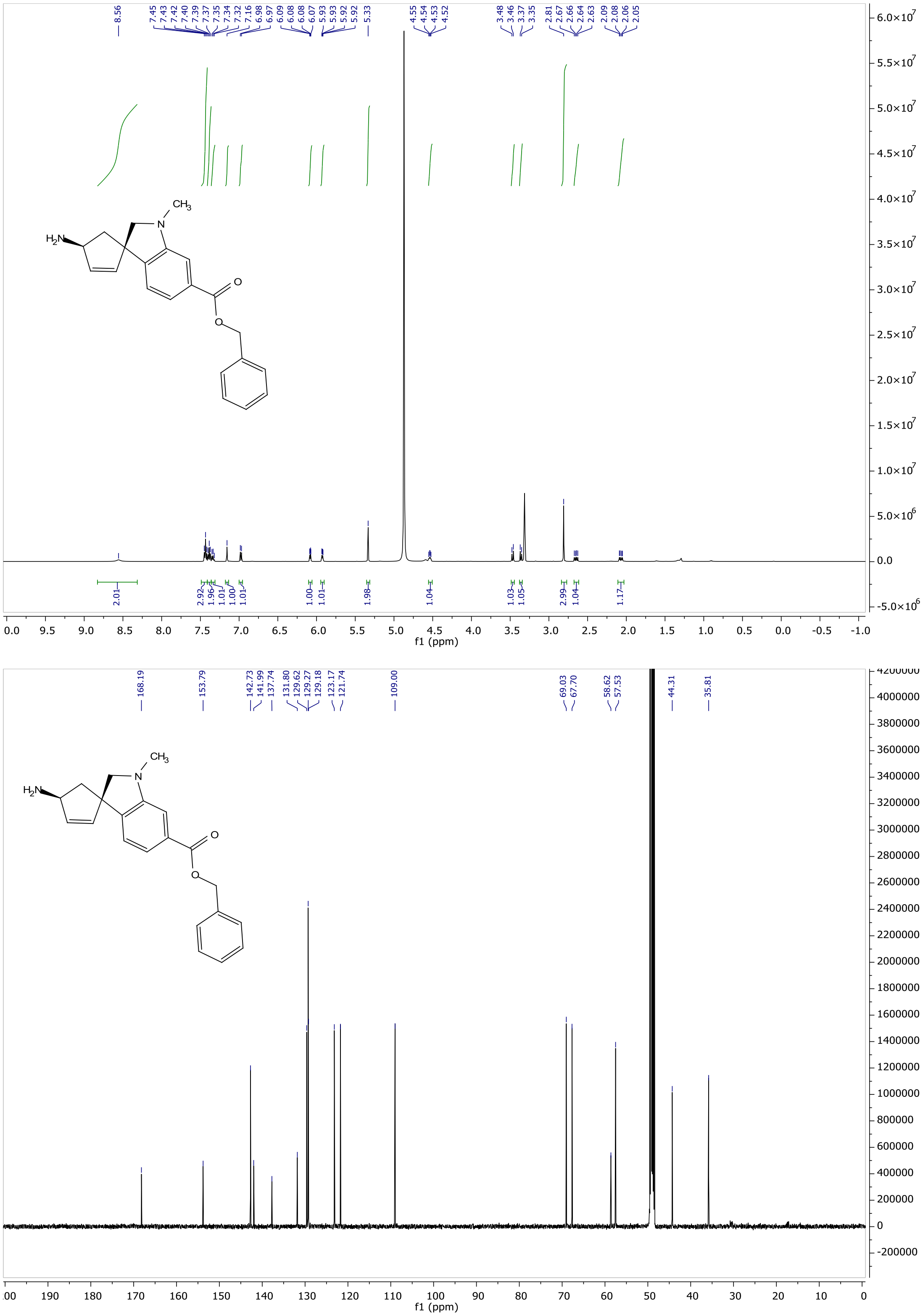


**Figure S50.** $^{1}$H and $^{13}$C{$^{1}$H} NMR spectra of **11** in MeOD.

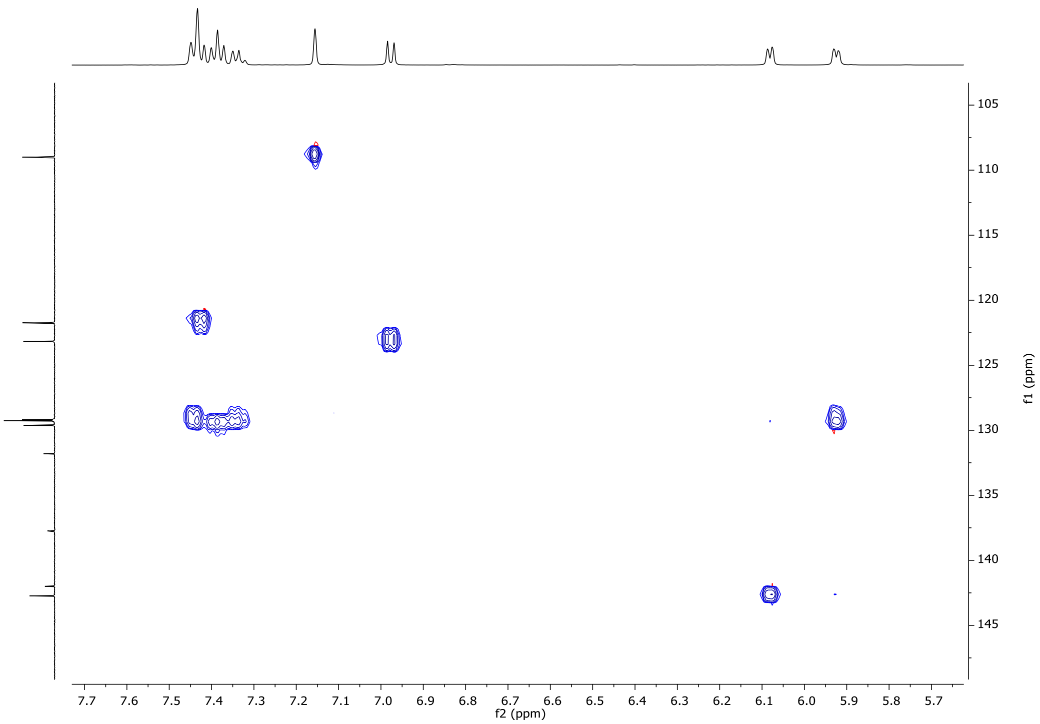


**Figure S51.** HSQC spectrum displaying overlap of carbon signals in the region between 129.1 – 129.7.

**(1*R*,4*S*)-4-(2,5-dimethyl-1H-pyrrol-1-yl)-1'-methylspiro[cyclopentane-1,3'-indolin]-2-ene-6'-carboxylic acid (12)**

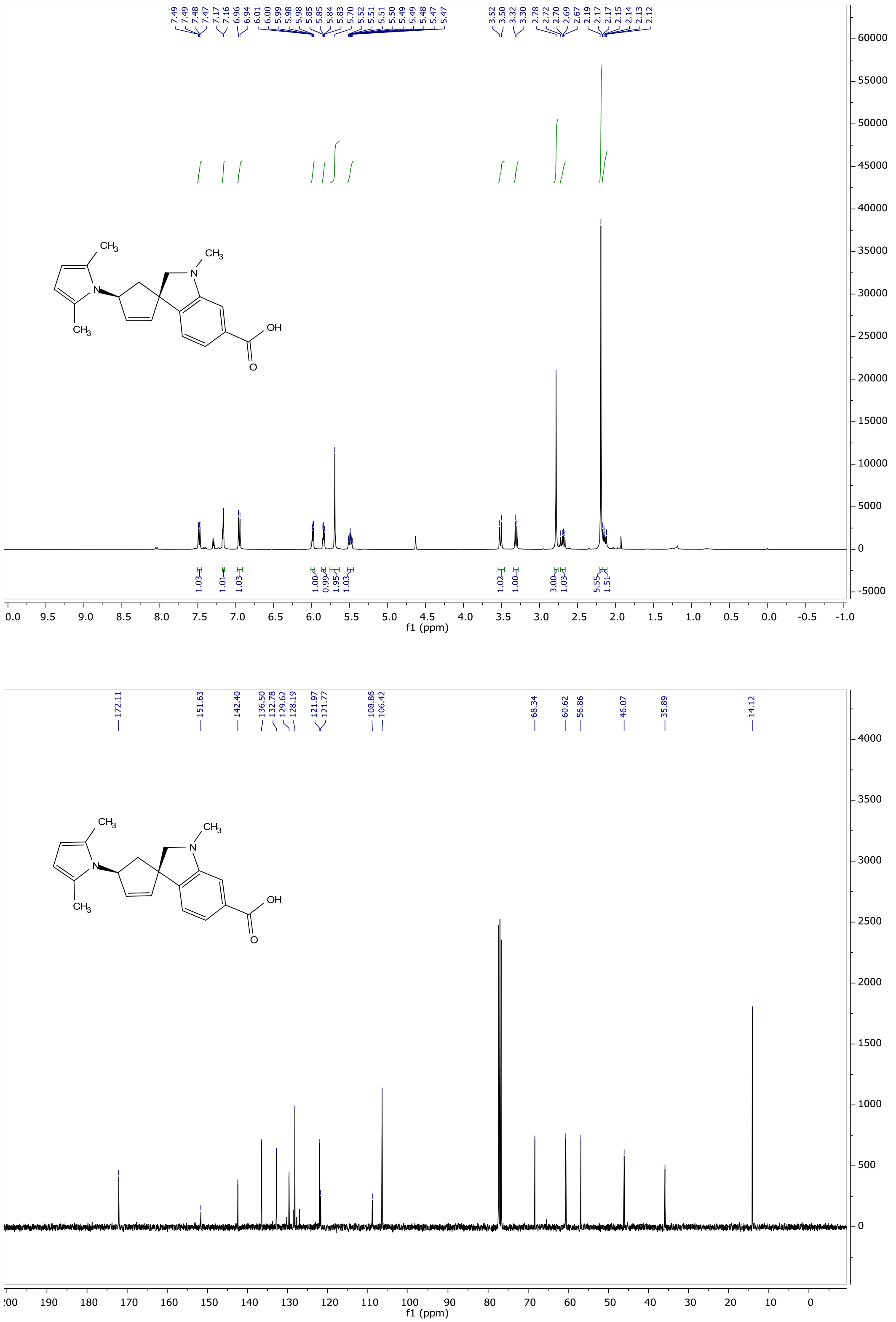

**Figure S52.** $^1H$ and $^{13}C\{^1H\}$ NMR spectra of **11** in $CDCl_3$.

# Cartesian coordinates for structures in figures S2 – S11

**π complex intermediate leading to *anti* product (figure S2)**

| | | | |
|---|---|---|---|
| C | -5.30360 | 0.35440 | 5.02940 |
| C | -6.63600 | 0.60490 | 5.77020 |
| C | -4.30740 | 1.08820 | 5.90880 |
| H | -6.66620 | -0.12030 | 6.58670 |
| C | -2.82610 | 1.03760 | 5.68660 |
| C | -3.89440 | -3.57280 | 5.44370 |
| C | -4.82000 | -3.52750 | 6.48390 |
| C | -4.80750 | -2.43870 | 7.36650 |
| C | -3.89280 | -1.39410 | 7.21480 |
| C | -3.01900 | -1.39780 | 6.10880 |
| C | -3.00910 | -2.51100 | 5.25060 |
| H | -3.87400 | -4.41890 | 4.76290 |
| H | -5.53150 | -4.33530 | 6.62940 |
| H | -5.50630 | -2.42860 | 8.19920 |
| H | -2.32950 | -2.53570 | 4.40490 |
| H | -5.30700 | 0.81940 | 4.03460 |
| C | -4.95690 | 1.99830 | 6.68240 |
| H | -4.47090 | 2.75610 | 7.28620 |
| H | -5.10050 | -0.70750 | 4.88750 |
| N | -2.18380 | -0.27880 | 5.89610 |
| C | -6.44550 | 1.98210 | 6.44490 |
| H | -6.71110 | 2.81040 | 5.77920 |
| H | -7.02330 | 2.09260 | 7.36420 |
| N | -7.83990 | 0.39160 | 4.98560 |
| C | -8.65910 | -0.72210 | 5.14920 |
| C | -9.62140 | -0.68630 | 4.16570 |
| C | -9.38310 | 0.46810 | 3.37100 |
| C | -8.28170 | 1.12020 | 3.88190 |
| H | -10.41000 | -1.41580 | 4.04100 |
| H | -9.95830 | 0.80530 | 2.51880 |
| C | -7.67800 | 2.40230 | 3.39770 |
| H | -6.58950 | 2.35700 | 3.28170 |
| H | -8.10090 | 2.63880 | 2.41760 |
| H | -7.89610 | 3.25220 | 4.05740 |
| C | -8.45040 | -1.74050 | 6.22540 |
| H | -7.47880 | -2.24530 | 6.14960 |
| H | -8.51680 | -1.31020 | 7.23350 |
| H | -9.22770 | -2.50440 | 6.14380 |

| | | | |
|---|---|---|---|
| Pd | -4.17330 | 0.14280 | 8.48300 |
| P | -2.73040 | -0.73430 | 10.19800 |
| C | -1.51180 | 0.70460 | 10.65280 |
| C | -0.44090 | 0.82800 | 9.54760 |
| H | 0.14730 | 1.73270 | 9.74150 |
| H | 0.25430 | -0.01140 | 9.52280 |
| H | -0.89460 | 0.93210 | 8.55750 |
| C | -2.25960 | 2.05340 | 10.67970 |
| H | -1.54000 | 2.83360 | 10.95790 |
| H | -2.67240 | 2.30690 | 9.70170 |
| H | -3.08270 | 2.08560 | 11.38950 |
| C | -0.81500 | 0.52090 | 12.01870 |
| H | -0.09890 | 1.34140 | 12.14960 |
| H | -1.51790 | 0.57630 | 12.85020 |
| H | -0.25850 | -0.41040 | 12.10610 |
| C | -3.79480 | -1.21270 | 11.75350 |
| C | -1.61900 | -2.27230 | 9.73670 |
| C | -2.49350 | -3.54130 | 9.63390 |
| H | -1.85430 | -4.36510 | 9.29490 |
| H | -2.92830 | -3.84590 | 10.58610 |
| H | -3.29130 | -3.42910 | 8.90240 |
| C | -0.48260 | -2.56920 | 10.74340 |
| H | 0.02270 | -3.48340 | 10.40910 |
| H | 0.27250 | -1.78230 | 10.75970 |
| H | -0.82360 | -2.74270 | 11.76240 |
| C | -0.95330 | -2.03670 | 8.34680 |
| H | -1.22450 | -2.83960 | 7.65800 |
| H | -1.24920 | -1.10770 | 7.86830 |
| H | 0.13710 | -2.03090 | 8.43740 |
| C | -5.07270 | -1.89830 | 11.21410 |
| H | -5.68120 | -2.22700 | 12.06530 |
| H | -5.66850 | -1.19350 | 10.62760 |
| H | -4.86380 | -2.77580 | 10.59970 |
| C | -4.24850 | 0.02870 | 12.55060 |
| H | -4.96300 | -0.30990 | 13.31090 |
| H | -3.42670 | 0.51220 | 13.08100 |
| H | -4.75910 | 0.76100 | 11.92850 |
| C | -3.08500 | -2.14560 | 12.75810 |
| H | -2.17660 | -1.69770 | 13.16720 |
| H | -3.76850 | -2.31370 | 13.59870 |
| H | -2.83800 | -3.12520 | 12.35030 |
| Br | -5.67700 | 1.86420 | 9.86550 |
| H | -2.33010 | 1.79020 | 6.31230 |

| | | | |
|---|---|---|---|
| H | -2.66330 | 1.33440 | 4.63450 |
| C | -1.00020 | -0.45280 | 5.07420 |
| H | -0.33910 | 0.40860 | 5.21680 |
| H | -0.45890 | -1.34860 | 5.38910 |
| H | -1.21530 | -0.53330 | 3.99380 |

**Migratory insertion transition state leading to *anti* product (Figure S3)**

| | | | |
|---|---|---|---|
| C | -5.35770 | 0.35860 | 5.02340 |
| C | -6.70080 | 0.37250 | 5.77450 |
| C | -4.38160 | 0.84480 | 6.08810 |
| H | -6.69660 | -0.51030 | 6.41190 |
| C | -2.94790 | 1.17000 | 5.72040 |
| C | -3.38730 | -3.55810 | 5.77450 |
| C | -4.50410 | -3.36270 | 6.59190 |
| C | -4.75220 | -2.09320 | 7.12040 |
| C | -3.87950 | -1.02470 | 6.86920 |
| C | -2.83150 | -1.20610 | 5.93780 |
| C | -2.55500 | -2.49230 | 5.43910 |
| H | -3.17670 | -4.54310 | 5.36840 |
| H | -5.16820 | -4.18790 | 6.82940 |
| H | -5.60550 | -1.95400 | 7.77760 |
| H | -1.72980 | -2.64760 | 4.75240 |
| H | -5.34880 | 1.11300 | 4.22700 |
| C | -5.10880 | 1.64210 | 7.01730 |
| H | -4.67430 | 2.54080 | 7.44810 |
| H | -5.11610 | -0.60910 | 4.58110 |
| N | -2.20620 | -0.06230 | 5.48790 |
| C | -6.59140 | 1.59450 | 6.71010 |
| H | -6.88540 | 2.51540 | 6.19150 |
| H | -7.21410 | 1.51020 | 7.60200 |
| N | -7.90170 | 0.27360 | 4.96310 |
| C | -8.86860 | -0.70670 | 5.16590 |
| C | -9.89920 | -0.47290 | 4.28450 |
| C | -9.56260 | 0.68010 | 3.52480 |
| C | -8.33230 | 1.13160 | 3.95170 |
| H | -10.79630 | -1.07160 | 4.20380 |
| H | -10.14730 | 1.13820 | 2.73820 |
| C | -7.55330 | 2.29020 | 3.40930 |
| H | -6.69000 | 1.98000 | 2.80540 |
| H | -8.20720 | 2.87340 | 2.75560 |
| H | -7.17970 | 2.96810 | 4.18420 |
| C | -8.73370 | -1.78590 | 6.19230 |

| | | | |
|---|---|---|---|
| H | -7.84700 | -2.41400 | 6.03320 |
| H | -8.67850 | -1.39400 | 7.21670 |
| H | -9.61010 | -2.43670 | 6.13920 |
| Pd | -4.19390 | 0.34990 | 8.52930 |
| P | -2.59920 | -0.57640 | 10.25200 |
| C | -1.73880 | 0.86550 | 11.22540 |
| C | -0.67030 | 1.52040 | 10.32340 |
| H | -0.35960 | 2.46490 | 10.78510 |
| H | 0.22560 | 0.90950 | 10.21070 |
| H | -1.06360 | 1.75350 | 9.32910 |
| C | -2.75720 | 1.97980 | 11.54150 |
| H | -2.23930 | 2.76290 | 12.11010 |
| H | -3.15810 | 2.42820 | 10.63180 |
| H | -3.60790 | 1.64710 | 12.13060 |
| C | -1.07050 | 0.43170 | 12.54750 |
| H | -0.53720 | 1.29420 | 12.96640 |
| H | -1.80040 | 0.11610 | 13.29380 |
| H | -0.34360 | -0.37050 | 12.42400 |
| C | -3.57350 | -1.68460 | 11.51910 |
| C | -1.16170 | -1.64480 | 9.48950 |
| C | -1.76360 | -2.96440 | 8.96470 |
| H | -0.98890 | -3.50800 | 8.41150 |
| H | -2.10380 | -3.62070 | 9.76650 |
| H | -2.59180 | -2.79600 | 8.28270 |
| C | 0.01410 | -2.00080 | 10.42300 |
| H | 0.71760 | -2.62790 | 9.86020 |
| H | 0.56930 | -1.12100 | 10.75160 |
| H | -0.28870 | -2.56490 | 11.30500 |
| C | -0.57310 | -0.88500 | 8.27140 |
| H | -0.17530 | -1.61190 | 7.55420 |
| H | -1.31350 | -0.28620 | 7.74540 |
| H | 0.25030 | -0.22930 | 8.55430 |
| C | -4.63780 | -2.45770 | 10.70400 |
| H | -5.18650 | -3.12350 | 11.38190 |
| H | -5.35730 | -1.76250 | 10.26280 |
| H | -4.21980 | -3.06930 | 9.90410 |
| C | -4.35450 | -0.81480 | 12.52860 |
| H | -5.01060 | -1.47800 | 13.10590 |
| H | -3.70500 | -0.30920 | 13.24460 |
| H | -4.98550 | -0.07580 | 12.03410 |
| C | -2.71260 | -2.67740 | 12.32560 |
| H | -1.93930 | -2.17680 | 12.91180 |
| H | -3.36470 | -3.20590 | 13.03190 |

| | | | |
|---|---|---|---|
| H | -2.23620 | -3.43650 | 11.70420 |
| Br | -5.89200 | 1.55140 | 10.13100 |
| H | -2.49380 | 1.77200 | 6.52120 |
| H | -2.94440 | 1.77700 | 4.80260 |
| C | -1.13470 | -0.09510 | 4.52030 |
| H | -0.70170 | 0.90320 | 4.42810 |
| H | -0.33760 | -0.76900 | 4.85330 |
| H | -1.46970 | -0.42350 | 3.52320 |

**σ complex intermediate leading to *anti* product (Figure S4)**

| | | | |
|---|---|---|---|
| C | -5.50280 | 0.09740 | 4.77500 |
| C | -6.84260 | -0.17150 | 5.48940 |
| C | -4.46700 | 0.12120 | 5.92780 |
| H | -6.93680 | -1.25220 | 5.59260 |
| C | -3.10960 | 0.74020 | 5.53140 |
| C | -2.91210 | -3.85570 | 6.28070 |
| C | -4.13900 | -3.63260 | 6.91220 |
| C | -4.70060 | -2.34880 | 6.91440 |
| C | -4.02190 | -1.30440 | 6.28520 |
| C | -2.78340 | -1.54530 | 5.64630 |
| C | -2.21220 | -2.82130 | 5.65180 |
| H | -2.48000 | -4.85250 | 6.28990 |
| H | -4.65400 | -4.44940 | 7.40710 |
| H | -5.65460 | -2.17120 | 7.39950 |
| H | -1.25520 | -3.01270 | 5.17820 |
| H | -5.51240 | 1.07710 | 4.29240 |
| C | -5.21480 | 0.82090 | 7.06260 |
| H | -5.06190 | 1.90450 | 7.06070 |
| H | -5.27570 | -0.65240 | 4.01110 |
| N | -2.33240 | -0.39120 | 5.02280 |
| C | -6.69200 | 0.43550 | 6.92250 |
| H | -7.34360 | 1.29700 | 7.07040 |
| H | -7.00080 | -0.29350 | 7.67650 |
| N | -8.02730 | 0.26160 | 4.75870 |
| C | -9.07770 | -0.58390 | 4.41650 |
| C | -10.05890 | 0.18060 | 3.82410 |
| C | -9.60670 | 1.52780 | 3.81170 |
| C | -8.35630 | 1.56540 | 4.39140 |
| H | -10.99960 | -0.19830 | 3.44720 |
| H | -10.12910 | 2.38890 | 3.41620 |
| C | -7.46750 | 2.75950 | 4.56710 |
| H | -6.67330 | 2.81490 | 3.81040 |

| | | | |
|---|---|---|---|
| H | -8.07160 | 3.66500 | 4.46290 |
| H | -6.98250 | 2.80100 | 5.54730 |
| C | -9.07120 | -2.05480 | 4.69230 |
| H | -8.23710 | -2.57650 | 4.20460 |
| H | -9.02050 | -2.28740 | 5.76450 |
| H | -9.99760 | -2.49050 | 4.30900 |
| Pd | -4.11900 | 0.21060 | 8.69860 |
| P | -2.47360 | -0.41320 | 10.50460 |
| C | -1.68140 | 1.12880 | 11.36380 |
| C | -0.69950 | 1.80940 | 10.38490 |
| H | -0.40970 | 2.78010 | 10.80410 |
| H | 0.21900 | 1.24070 | 10.23130 |
| H | -1.16620 | 2.00010 | 9.41320 |
| C | -2.78360 | 2.17720 | 11.63710 |
| H | -2.31530 | 3.05350 | 12.10280 |
| H | -3.26950 | 2.49810 | 10.71460 |
| H | -3.56770 | 1.82920 | 12.30480 |
| C | -0.93740 | 0.82930 | 12.68090 |
| H | -0.47610 | 1.75610 | 13.04480 |
| H | -1.61170 | 0.48070 | 13.46490 |
| H | -0.14120 | 0.09250 | 12.56500 |
| C | -3.34020 | -1.50110 | 11.84890 |
| C | -1.03540 | -1.44320 | 9.71360 |
| C | -1.58190 | -2.83190 | 9.32960 |
| H | -0.83680 | -3.34890 | 8.71430 |
| H | -1.78090 | -3.46470 | 10.19430 |
| H | -2.49170 | -2.75780 | 8.73340 |
| C | 0.23340 | -1.63020 | 10.56590 |
| H | 0.93250 | -2.27860 | 10.02220 |
| H | 0.75240 | -0.68890 | 10.75480 |
| H | 0.02870 | -2.10480 | 11.52660 |
| C | -0.64150 | -0.76730 | 8.37860 |
| H | 0.05820 | -1.42140 | 7.84350 |
| H | -1.51080 | -0.63290 | 7.73270 |
| H | -0.15500 | 0.19800 | 8.50290 |
| C | -4.35910 | -2.41010 | 11.11710 |
| H | -4.88670 | -3.01640 | 11.86370 |
| H | -5.10300 | -1.80820 | 10.58770 |
| H | -3.90340 | -3.09420 | 10.40250 |
| C | -4.17770 | -0.60900 | 12.79270 |
| H | -4.78570 | -1.26430 | 13.42860 |
| H | -3.56270 | -0.00170 | 13.45860 |
| H | -4.85900 | 0.04190 | 12.24130 |

| | | | |
|---|---|---|---|
| C | -2.39830 | -2.36490 | 12.71060 |
| H | -1.66070 | -1.76620 | 13.24880 |
| H | -2.99620 | -2.89810 | 13.46040 |
| H | -1.86580 | -3.12080 | 12.13100 |
| Br | -5.91420 | 1.44880 | 10.04470 |
| H | -2.62050 | 1.17870 | 6.42380 |
| H | -3.20100 | 1.52520 | 4.77420 |
| C | -0.94250 | -0.19250 | 4.67510 |
| H | -0.85660 | 0.68580 | 4.02990 |
| H | -0.30150 | -0.04070 | 5.55760 |
| H | -0.56700 | -1.05380 | 4.11670 |

**Reductive elimination transition step leading to *anti* product (Figure S5)**

| | | | |
|---|---|---|---|
| C | -5.53290 | -0.06130 | 4.81480 |
| C | -6.88960 | 0.06130 | 5.54920 |
| C | -4.47970 | 0.16190 | 5.94740 |
| H | -7.09360 | -0.88980 | 6.03870 |
| C | -3.09650 | 0.67300 | 5.47830 |
| C | -2.94900 | -3.61430 | 7.28430 |
| C | -4.24550 | -3.30430 | 7.69660 |
| C | -4.82110 | -2.08190 | 7.32360 |
| C | -4.09180 | -1.18520 | 6.55750 |
| C | -2.80360 | -1.52360 | 6.10970 |
| C | -2.21240 | -2.73250 | 6.48610 |
| H | -2.49760 | -4.55470 | 7.58800 |
| H | -4.80320 | -3.99800 | 8.31770 |
| H | -5.82070 | -1.82660 | 7.66430 |
| H | -1.20520 | -2.98610 | 6.17260 |
| H | -5.44540 | 0.73390 | 4.07000 |
| C | -5.20300 | 1.19590 | 6.82000 |
| H | -4.68630 | 2.08730 | 7.15730 |
| H | -5.41910 | -1.02200 | 4.30700 |
| N | -2.29400 | -0.53590 | 5.26250 |
| C | -6.58780 | 1.13200 | 6.61970 |
| H | -7.25280 | 1.98290 | 6.72570 |
| H | -7.26770 | 0.47370 | 7.99850 |
| N | -8.04680 | 0.35340 | 4.72370 |
| C | -9.16940 | -0.46910 | 4.65250 |
| C | -10.10830 | 0.17160 | 3.87690 |
| C | -9.55670 | 1.41650 | 3.46710 |
| C | -8.28700 | 1.51740 | 3.99160 |
| H | -11.08330 | -0.22480 | 3.62820 |

| | | | |
|---|---|---|---|
| H | -10.02470 | 2.16220 | 2.83850 |
| C | -7.29090 | 2.61580 | 3.77340 |
| H | -6.52100 | 2.34480 | 3.03940 |
| H | -7.81470 | 3.49160 | 3.38180 |
| H | -6.76860 | 2.92530 | 4.68400 |
| C | -9.24560 | -1.81320 | 5.30620 |
| H | -8.45410 | -2.49250 | 4.96460 |
| H | -9.18650 | -1.76690 | 6.40050 |
| H | -10.20340 | -2.27400 | 5.05290 |
| Pd | -5.91360 | 0.52620 | 8.81370 |
| P | -7.04830 | -0.16350 | 10.79660 |
| C | -6.43430 | -1.89340 | 11.40040 |
| C | -7.00000 | -2.99840 | 10.48240 |
| H | -6.48720 | -3.93770 | 10.71970 |
| H | -8.06780 | -3.17060 | 10.62900 |
| H | -6.81670 | -2.78670 | 9.42630 |
| C | -4.89650 | -1.95030 | 11.24490 |
| H | -4.55680 | -2.93750 | 11.58290 |
| H | -4.58830 | -1.82410 | 10.20580 |
| H | -4.36890 | -1.19940 | 11.82740 |
| C | -6.81650 | -2.23460 | 12.85570 |
| H | -6.46540 | -3.24970 | 13.07860 |
| H | -6.34460 | -1.56850 | 13.57820 |
| H | -7.89350 | -2.21780 | 13.02880 |
| C | -6.74300 | 1.14570 | 12.19570 |
| C | -8.96470 | -0.26940 | 10.47360 |
| C | -9.51280 | 1.16030 | 10.27070 |
| H | -10.55180 | 1.09240 | 9.92730 |
| H | -9.51350 | 1.75480 | 11.18430 |
| H | -8.94710 | 1.69900 | 9.50440 |
| C | -9.77070 | -0.97230 | 11.58760 |
| H | -10.83970 | -0.88270 | 11.35940 |
| H | -9.54550 | -2.03920 | 11.64250 |
| H | -9.61220 | -0.53970 | 12.57420 |
| C | -9.26110 | -1.01140 | 9.14830 |
| H | -10.34820 | -1.04550 | 9.00900 |
| H | -8.84120 | -0.48070 | 8.29170 |
| H | -8.89720 | -2.03630 | 9.12190 |
| C | -6.71990 | 2.54430 | 11.53230 |
| H | -6.54430 | 3.29450 | 12.31320 |
| H | -5.90340 | 2.61590 | 10.80900 |
| H | -7.65150 | 2.80670 | 11.03160 |
| C | -5.34540 | 0.95160 | 12.82430 |

| | | | |
|---|---|---|---|
| H | -5.16160 | 1.79950 | 13.49520 |
| H | -5.27170 | 0.04700 | 13.42910 |
| H | -4.55500 | 0.95100 | 12.07260 |
| C | -7.77990 | 1.13300 | 13.33600 |
| H | -7.83540 | 0.16610 | 13.84090 |
| H | -7.47550 | 1.87310 | 14.08570 |
| H | -8.78300 | 1.40880 | 13.00760 |
| Br | -3.46000 | 1.01220 | 9.62770 |
| H | -2.65460 | 1.28810 | 6.27970 |
| H | -3.15300 | 1.27460 | 4.56440 |
| C | -0.86130 | -0.35220 | 5.13630 |
| H | -0.66240 | 0.40920 | 4.37660 |
| H | -0.38940 | -0.03610 | 6.08160 |
| H | -0.39030 | -1.28130 | 4.80410 |

**Product 9a resulting from *anti* Mizoroki-Heck pathway (Figure S6)**

| | | | |
|---|---|---|---|
| C | -6.05330 | -0.85060 | 5.24080 |
| C | -7.31680 | -0.13150 | 5.80900 |
| C | -4.80280 | -0.23960 | 5.97160 |
| H | -7.89750 | -0.84810 | 6.38500 |
| C | -3.77600 | 0.41090 | 4.99610 |
| C | -2.00700 | -3.22530 | 7.24610 |
| C | -3.09470 | -3.00540 | 8.09290 |
| C | -4.05040 | -2.03390 | 7.76260 |
| C | -3.90340 | -1.30010 | 6.59880 |
| C | -2.80500 | -1.52580 | 5.75020 |
| C | -1.84300 | -2.48710 | 6.06730 |
| H | -1.26260 | -3.97200 | 7.50850 |
| H | -3.19650 | -3.57390 | 9.01230 |
| H | -4.89340 | -1.83550 | 8.41390 |
| H | -0.98360 | -2.65610 | 5.42700 |
| H | -5.98770 | -0.71260 | 4.15990 |
| C | -5.40420 | 0.79400 | 6.89690 |
| H | -4.78880 | 1.46260 | 7.48930 |
| H | -6.11250 | -1.92520 | 5.42410 |
| N | -2.87040 | -0.68510 | 4.63480 |
| C | -6.74990 | 0.90120 | 6.77290 |
| H | -7.35330 | 1.70550 | 7.18290 |
| H | -6.97490 | -1.35630 | 8.45320 |
| N | -8.25830 | 0.41140 | 4.83550 |
| C | -9.59240 | 0.01730 | 4.76720 |
| C | -10.20660 | 0.77790 | 3.79860 |

| | | | |
|---|---|---|---|
| C | -9.23360 | 1.66640 | 3.26550 |
| C | -8.04040 | 1.43280 | 3.91340 |
| H | -11.24680 | 0.69900 | 3.51330 |
| H | -9.37810 | 2.40230 | 2.48580 |
| C | -6.71930 | 2.09230 | 3.67880 |
| H | -6.02840 | 1.45460 | 3.11240 |
| H | -6.87550 | 2.99930 | 3.08930 |
| H | -6.21530 | 2.38140 | 4.60600 |
| C | -10.18410 | -1.04220 | 5.64200 |
| H | -9.68740 | -2.01560 | 5.53390 |
| H | -10.15780 | -0.77940 | 6.70820 |
| H | -11.23310 | -1.18030 | 5.36820 |
| Pd | -6.70720 | 0.01500 | 9.07000 |
| P | -7.65060 | -1.15530 | 10.84580 |
| C | -6.25540 | -2.24110 | 11.63020 |
| C | -5.97270 | -3.47140 | 10.74150 |
| H | -5.06330 | -3.95810 | 11.11290 |
| H | -6.77100 | -4.21440 | 10.77890 |
| H | -5.79410 | -3.20130 | 9.69970 |
| C | -4.95260 | -1.40540 | 11.65860 |
| H | -4.16280 | -2.01450 | 12.11510 |
| H | -4.62690 | -1.13450 | 10.65160 |
| H | -5.03770 | -0.48480 | 12.23210 |
| C | -6.56970 | -2.74620 | 13.05400 |
| H | -5.74310 | -3.38740 | 13.38270 |
| H | -6.65320 | -1.93650 | 13.77870 |
| H | -7.48140 | -3.34420 | 13.09910 |
| C | -8.35940 | 0.05860 | 12.18860 |
| C | -9.09600 | -2.31460 | 10.24750 |
| C | -10.32290 | -1.44230 | 9.90410 |
| H | -11.06210 | -2.06860 | 9.39140 |
| H | -10.81040 | -1.02300 | 10.78420 |
| H | -10.05630 | -0.62450 | 9.22760 |
| C | -9.50100 | -3.37980 | 11.29110 |
| H | -10.39600 | -3.89910 | 10.92860 |
| H | -8.72590 | -4.13670 | 11.42550 |
| H | -9.74300 | -2.96200 | 12.26670 |
| C | -8.73900 | -3.05690 | 8.93760 |
| H | -9.56030 | -3.74600 | 8.70680 |
| H | -8.64730 | -2.36390 | 8.09940 |
| H | -7.82500 | -3.64450 | 8.99300 |
| C | -9.12210 | 1.20670 | 11.48490 |
| H | -9.45980 | 1.90610 | 12.25910 |

| | | | |
|---|---|---|---|
| H | -8.47570 | 1.76020 | 10.80300 |
| H | -10.00700 | 0.87340 | 10.94550 |
| C | -7.19280 | 0.71170 | 12.96280 |
| H | -7.61640 | 1.49090 | 13.60700 |
| H | -6.66910 | 0.01060 | 13.61410 |
| H | -6.48120 | 1.19860 | 12.29470 |
| C | -9.30530 | -0.60910 | 13.20880 |
| H | -8.84400 | -1.44070 | 13.74410 |
| H | -9.57820 | 0.14510 | 13.95610 |
| H | -10.23590 | -0.95830 | 12.75830 |
| Br | -5.94980 | 2.39440 | 9.94710 |
| H | -3.22500 | 1.21380 | 5.51890 |
| H | -4.24200 | 0.84040 | 4.10820 |
| C | -1.66190 | -0.33100 | 3.91820 |
| H | -1.92460 | 0.27490 | 3.04630 |
| H | -0.94890 | 0.23910 | 4.53840 |
| H | -1.16170 | -1.23360 | 3.55650 |

**π complex intermediate leading to *syn* product (figure S7)**

| | | | |
|---|---|---|---|
| C | -5.21690 | 0.50500 | 4.99890 |
| C | -6.52620 | 1.34770 | 5.02130 |
| C | -4.33520 | 1.20340 | 6.01760 |
| H | -6.44050 | 2.07890 | 4.21680 |
| C | -2.83660 | 1.15870 | 5.98430 |
| C | -3.57920 | -3.53770 | 5.37980 |
| C | -4.47250 | -3.70700 | 6.43510 |
| C | -4.60470 | -2.68830 | 7.38650 |
| C | -3.85970 | -1.50990 | 7.29520 |
| C | -2.97250 | -1.32680 | 6.21610 |
| C | -2.84980 | -2.35380 | 5.26450 |
| H | -3.45510 | -4.31870 | 4.63500 |
| H | -5.05600 | -4.61830 | 6.52930 |
| H | -5.29050 | -2.82810 | 8.21830 |
| H | -2.17730 | -2.22800 | 4.42200 |
| H | -4.75970 | 0.49280 | 4.00220 |
| C | -5.05040 | 2.07610 | 6.76060 |
| H | -4.63040 | 2.72370 | 7.52130 |
| H | -5.39410 | -0.53920 | 5.26390 |
| N | -2.18350 | -0.15680 | 6.14120 |
| C | -6.49560 | 2.14160 | 6.35920 |
| H | -6.83280 | 3.17540 | 6.22220 |
| H | -7.14100 | 1.71510 | 7.13080 |

| | | | |
|---|---|---|---|
| N | -7.75660 | 0.61460 | 4.76570 |
| C | -8.61430 | 0.88550 | 3.70320 |
| C | -9.69990 | 0.04690 | 3.81320 |
| C | -9.50900 | -0.75430 | 4.97150 |
| C | -8.31090 | -0.39540 | 5.55080 |
| H | -10.53770 | 0.02230 | 3.12950 |
| H | -10.16960 | -1.52230 | 5.35110 |
| C | -7.66340 | -0.98910 | 6.76310 |
| H | -7.37600 | -0.25430 | 7.52360 |
| H | -8.37230 | -1.67580 | 7.23440 |
| H | -6.76640 | -1.56800 | 6.52010 |
| C | -8.33950 | 1.92960 | 2.66830 |
| H | -8.27860 | 2.94310 | 3.08740 |
| H | -7.40850 | 1.74900 | 2.11440 |
| H | -9.15530 | 1.92870 | 1.94100 |
| Pd | -4.37920 | -0.11260 | 8.61710 |
| P | -2.81120 | -0.82630 | 10.25020 |
| C | -1.58960 | 0.67240 | 10.39290 |
| C | -0.59820 | 0.63630 | 9.21100 |
| H | -0.05330 | 1.58770 | 9.19080 |
| H | 0.14560 | -0.15720 | 9.30600 |
| H | -1.11610 | 0.51830 | 8.25500 |
| C | -2.39080 | 1.98650 | 10.24950 |
| H | -1.69450 | 2.82620 | 10.36550 |
| H | -2.85040 | 2.06440 | 9.26120 |
| H | -3.18620 | 2.10280 | 10.98220 |
| C | -0.79340 | 0.72660 | 11.71320 |
| H | -0.11440 | 1.58670 | 11.66610 |
| H | -1.43340 | 0.87310 | 12.58310 |
| H | -0.18080 | -0.15750 | 11.88360 |
| C | -3.74970 | -1.05940 | 11.94590 |
| C | -1.75050 | -2.44330 | 9.96730 |
| C | -2.62970 | -3.69710 | 10.17030 |
| H | -2.03950 | -4.57160 | 9.87160 |
| H | -2.94200 | -3.85620 | 11.20150 |
| H | -3.51400 | -3.67330 | 9.53210 |
| C | -0.52690 | -2.52580 | 10.91150 |
| H | -0.08930 | -3.52590 | 10.80770 |
| H | 0.24810 | -1.81080 | 10.62970 |
| H | -0.75960 | -2.38430 | 11.96400 |
| C | -1.20490 | -2.55220 | 8.52010 |
| H | -1.93700 | -2.99950 | 7.85470 |
| H | -0.89450 | -1.60730 | 8.08440 |

| | | | |
|---|---|---|---|
| H | -0.33250 | -3.21600 | 8.53900 |
| C | -5.05300 | -1.83460 | 11.63850 |
| H | -5.58160 | -2.01240 | 12.58280 |
| H | -5.71130 | -1.24820 | 10.99350 |
| H | -4.87960 | -2.80570 | 11.17320 |
| C | -4.16330 | 0.29300 | 12.56380 |
| H | -4.81160 | 0.07810 | 13.42170 |
| H | -3.31320 | 0.86180 | 12.94340 |
| H | -4.73910 | 0.90960 | 11.87710 |
| C | -2.94500 | -1.80370 | 13.03480 |
| H | -2.02680 | -1.27740 | 13.30490 |
| H | -3.56790 | -1.84440 | 13.93580 |
| H | -2.69460 | -2.83180 | 12.77740 |
| Br | -6.11400 | 1.45730 | 9.85320 |
| H | -2.43420 | 1.85700 | 6.73020 |
| H | -2.55010 | 1.55610 | 4.99400 |
| C | -0.92610 | -0.22820 | 5.41580 |
| H | -0.29990 | 0.62150 | 5.70970 |
| H | -0.39490 | -1.14600 | 5.67960 |
| H | -1.04100 | -0.19220 | 4.31820 |

**Migratory insertion transition state leading to *syn* product (Figure S8)**

| | | | |
|---|---|---|---|
| C | -5.40520 | 0.21870 | 5.04420 |
| C | -6.65160 | 1.11640 | 5.26430 |
| C | -4.42160 | 0.71600 | 6.11190 |
| H | -6.47050 | 2.04440 | 4.71810 |
| C | -2.98750 | 1.04100 | 5.73380 |
| C | -3.39030 | -3.68360 | 5.88970 |
| C | -4.49080 | -3.47870 | 6.72790 |
| C | -4.73170 | -2.20150 | 7.24060 |
| C | -3.86550 | -1.13830 | 6.95540 |
| C | -2.84160 | -1.32640 | 6.00170 |
| C | -2.57170 | -2.62010 | 5.51650 |
| H | -3.18510 | -4.67450 | 5.49490 |
| H | -5.14860 | -4.30160 | 6.98950 |
| H | -5.58160 | -2.04550 | 7.89760 |
| H | -1.76350 | -2.78370 | 4.81170 |
| H | -4.98050 | 0.34220 | 4.04140 |
| C | -5.12290 | 1.51700 | 7.04940 |
| H | -4.66850 | 2.41160 | 7.46860 |
| H | -5.64410 | -0.83630 | 5.15580 |
| N | -2.22850 | -0.18570 | 5.52290 |

| | | | |
|---|---|---|---|
| C | -6.61250 | 1.48080 | 6.76930 |
| H | -7.09980 | 2.43500 | 6.98030 |
| H | -7.11520 | 0.73640 | 7.38790 |
| N | -7.90770 | 0.59100 | 4.75510 |
| C | -8.67210 | 1.23720 | 3.78570 |
| C | -9.79860 | 0.47930 | 3.56200 |
| C | -9.73030 | -0.65660 | 4.41250 |
| C | -8.56490 | -0.57820 | 5.14230 |
| H | -10.58440 | 0.72700 | 2.86120 |
| H | -10.45320 | -1.45740 | 4.49280 |
| C | -8.06590 | -1.56190 | 6.15380 |
| H | -7.86900 | -1.11670 | 7.13510 |
| H | -8.83230 | -2.32870 | 6.29390 |
| H | -7.14730 | -2.07510 | 5.84700 |
| C | -8.27440 | 2.53280 | 3.15140 |
| H | -8.18400 | 3.35310 | 3.87560 |
| H | -7.32270 | 2.46930 | 2.60690 |
| H | -9.04210 | 2.82100 | 2.42900 |
| Pd | -4.17420 | 0.26770 | 8.57850 |
| P | -2.57840 | -0.61280 | 10.29890 |
| C | -1.73710 | 0.86190 | 11.23890 |
| C | -0.67840 | 1.51100 | 10.32140 |
| H | -0.38050 | 2.46980 | 10.76150 |
| H | 0.22570 | 0.91000 | 10.22140 |
| H | -1.07580 | 1.71610 | 9.32260 |
| C | -2.77110 | 1.96860 | 11.53090 |
| H | -2.26460 | 2.77030 | 12.08360 |
| H | -3.17710 | 2.39270 | 10.61190 |
| H | -3.61840 | 1.63690 | 12.12550 |
| C | -1.06220 | 0.46610 | 12.56950 |
| H | -0.54050 | 1.34480 | 12.96900 |
| H | -1.78730 | 0.15680 | 13.32310 |
| H | -0.32440 | -0.32860 | 12.46270 |
| C | -3.53820 | -1.70450 | 11.59010 |
| C | -1.13020 | -1.67900 | 9.55430 |
| C | -1.71500 | -3.01820 | 9.06000 |
| H | -0.93130 | -3.56550 | 8.52330 |
| H | -2.05020 | -3.65900 | 9.87620 |
| H | -2.54220 | -2.87640 | 8.37070 |
| C | 0.05130 | -1.99790 | 10.49410 |
| H | 0.76300 | -2.62720 | 9.94450 |
| H | 0.59430 | -1.10330 | 10.80260 |
| H | -0.24290 | -2.54660 | 11.38870 |

| | | | |
|---|---|---|---|
| C | -0.55380 | -0.93980 | 8.31810 |
| H | -0.13860 | -1.67760 | 7.62210 |
| H | -1.30430 | -0.37080 | 7.77370 |
| H | 0.25530 | -0.26020 | 8.58550 |
| C | -4.59240 | -2.51080 | 10.79480 |
| H | -5.12800 | -3.17100 | 11.48850 |
| H | -5.32450 | -1.83620 | 10.34260 |
| H | -4.16800 | -3.13200 | 10.00590 |
| C | -4.33120 | -0.82090 | 12.57790 |
| H | -4.97960 | -1.47860 | 13.17020 |
| H | -3.68930 | -0.29000 | 13.28240 |
| H | -4.97130 | -0.10220 | 12.06510 |
| C | -2.66330 | -2.66640 | 12.41860 |
| H | -1.89560 | -2.14240 | 12.99150 |
| H | -3.30770 | -3.18640 | 13.13820 |
| H | -2.17820 | -3.43370 | 11.81410 |
| Br | -5.91660 | 1.48400 | 10.13490 |
| H | -2.53820 | 1.66350 | 6.52100 |
| H | -2.99070 | 1.62870 | 4.80330 |
| C | -1.20390 | -0.23510 | 4.50580 |
| H | -0.77390 | 0.76050 | 4.37820 |
| H | -0.39290 | -0.90470 | 4.81200 |
| H | -1.58530 | -0.57880 | 3.53020 |

**σ complex intermediate leading to *syn* product (Figure S9)**

| | | | |
|---|---|---|---|
| C | -5.52790 | 0.17740 | 4.79340 |
| C | -6.72400 | 0.98900 | 5.34930 |
| C | -4.46390 | 0.18500 | 5.94520 |
| H | -6.53810 | 2.04140 | 5.12470 |
| C | -3.07760 | 0.71810 | 5.51530 |
| C | -3.12870 | -3.86070 | 6.36540 |
| C | -4.32850 | -3.55200 | 7.01390 |
| C | -4.81470 | -2.23780 | 6.99670 |
| C | -4.08270 | -1.24930 | 6.33580 |
| C | -2.87360 | -1.57610 | 5.67980 |
| C | -2.37910 | -2.88360 | 5.70220 |
| H | -2.75780 | -4.88160 | 6.38730 |
| H | -4.88340 | -4.32700 | 7.53220 |
| H | -5.75650 | -1.99570 | 7.47700 |
| H | -1.44560 | -3.14220 | 5.21400 |
| H | -5.13930 | 0.61550 | 3.86890 |
| C | -5.11370 | 0.95780 | 7.10150 |

| | | | |
|---|---|---|---|
| H | -4.78460 | 2.00350 | 7.15180 |
| H | -5.83350 | -0.84320 | 4.55630 |
| N | -2.37370 | -0.46410 | 5.01750 |
| C | -6.61570 | 0.85380 | 6.88430 |
| H | -7.17860 | 1.61810 | 7.41920 |
| H | -6.99130 | -0.11460 | 7.21910 |
| N | -8.01170 | 0.67340 | 4.74290 |
| C | -8.68790 | 1.54000 | 3.88860 |
| C | -9.81010 | 0.88980 | 3.42680 |
| C | -9.82340 | -0.40830 | 4.00380 |
| C | -8.71210 | -0.53110 | 4.81030 |
| H | -10.53820 | 1.31310 | 2.74820 |
| H | -10.56960 | -1.17890 | 3.86300 |
| C | -8.33440 | -1.70920 | 5.65430 |
| H | -8.49670 | -1.53310 | 6.72540 |
| H | -8.96320 | -2.55640 | 5.36780 |
| H | -7.29210 | -2.02160 | 5.53290 |
| C | -8.20950 | 2.92130 | 3.56980 |
| H | -8.13430 | 3.56170 | 4.45830 |
| H | -7.22930 | 2.93170 | 3.07450 |
| H | -8.92220 | 3.39270 | 2.88840 |
| Pd | -4.11800 | 0.17960 | 8.73140 |
| P | -2.49110 | -0.50950 | 10.53030 |
| C | -1.71180 | 1.02830 | 11.41010 |
| C | -0.75010 | 1.74040 | 10.43360 |
| H | -0.46720 | 2.70660 | 10.86780 |
| H | 0.17320 | 1.18610 | 10.25870 |
| H | -1.23040 | 1.94350 | 9.47100 |
| C | -2.82550 | 2.05580 | 11.71550 |
| H | -2.36430 | 2.92910 | 12.19440 |
| H | -3.32530 | 2.38850 | 10.80470 |
| H | -3.59770 | 1.68280 | 12.38380 |
| C | -0.94920 | 0.71460 | 12.71330 |
| H | -0.49730 | 1.64090 | 13.09000 |
| H | -1.60960 | 0.34080 | 13.49720 |
| H | -0.14400 | -0.00790 | 12.57460 |
| C | -3.33600 | -1.63180 | 11.86510 |
| C | -1.04480 | -1.50970 | 9.71400 |
| C | -1.57900 | -2.89860 | 9.31220 |
| H | -0.83560 | -3.39490 | 8.67710 |
| H | -1.75820 | -3.55040 | 10.16760 |
| H | -2.49800 | -2.82350 | 8.73090 |
| C | 0.23190 | -1.69370 | 10.55580 |

| | | | |
|---|---|---|---|
| H | 0.93400 | -2.32810 | 9.99960 |
| H | 0.74230 | -0.74910 | 10.75240 |
| H | 0.03910 | -2.18210 | 11.51210 |
| C | -0.66620 | -0.80940 | 8.38640 |
| H | 0.04560 | -1.44390 | 7.84320 |
| H | -1.53910 | -0.68460 | 7.74310 |
| H | -0.19770 | 0.16350 | 8.52160 |
| C | -4.35610 | -2.53060 | 11.12320 |
| H | -4.87060 | -3.15920 | 11.86080 |
| H | -5.10980 | -1.92110 | 10.61690 |
| H | -3.90310 | -3.19350 | 10.38680 |
| C | -4.16720 | -0.76800 | 12.83990 |
| H | -4.75820 | -1.44330 | 13.47090 |
| H | -3.54780 | -0.16870 | 13.50900 |
| H | -4.86410 | -0.11360 | 12.31320 |
| C | -2.37610 | -2.50330 | 12.69880 |
| H | -1.63920 | -1.90750 | 13.24150 |
| H | -2.96110 | -3.05670 | 13.44390 |
| H | -1.84200 | -3.24260 | 12.10020 |
| Br | -5.96050 | 1.26060 | 10.14820 |
| H | -2.55310 | 1.14170 | 6.39420 |
| H | -3.13400 | 1.49460 | 4.74590 |
| C | -0.98370 | -0.35200 | 4.63080 |
| H | -0.86280 | 0.51480 | 3.97510 |
| H | -0.31040 | -0.23260 | 5.49470 |
| H | -0.67660 | -1.23920 | 4.07050 |

**Reductive elimination transition step leading to *syn* product (Figure S10)**

| | | | |
|---|---|---|---|
| C | -5.88420 | -0.58050 | 5.24800 |
| C | -6.59840 | 0.73100 | 4.88780 |
| C | -4.59940 | -0.14020 | 6.00330 |
| H | -6.07910 | 1.18630 | 4.03080 |
| C | -3.42240 | 0.15340 | 5.02330 |
| C | -2.66920 | -3.54100 | 7.72420 |
| C | -3.73160 | -3.00730 | 8.45590 |
| C | -4.40870 | -1.87460 | 7.98590 |
| C | -4.00480 | -1.29230 | 6.79480 |
| C | -2.94990 | -1.84720 | 6.04990 |
| C | -2.26120 | -2.97040 | 6.51200 |
| H | -2.13910 | -4.41130 | 8.10180 |
| H | -4.02130 | -3.45590 | 9.40080 |
| H | -5.21400 | -1.43480 | 8.56300 |

| | | | |
|---|---|---|---|
| H | -1.43120 | -3.39310 | 5.95500 |
| H | -5.64940 | -1.20970 | 4.38850 |
| C | -5.04830 | 1.14270 | 6.68450 |
| H | -4.33130 | 1.78760 | 7.17950 |
| H | -6.49780 | -1.15160 | 5.94550 |
| N | -2.76700 | -1.14450 | 4.84760 |
| C | -6.23660 | 1.61410 | 6.09160 |
| H | -6.47580 | 2.66990 | 6.08970 |
| H | -7.50910 | 1.14820 | 6.99550 |
| N | -8.02240 | 0.66170 | 4.54150 |
| C | -8.75750 | 1.81020 | 4.22890 |
| C | -10.00460 | 1.41600 | 3.80420 |
| C | -10.04040 | -0.00090 | 3.83030 |
| C | -8.81740 | -0.45930 | 4.27030 |
| H | -10.79450 | 2.08790 | 3.49700 |
| H | -10.87100 | -0.63990 | 3.56220 |
| C | -8.45650 | -1.90290 | 4.45160 |
| H | -8.29160 | -2.17880 | 5.49910 |
| H | -9.29730 | -2.50030 | 4.09020 |
| H | -7.57260 | -2.20990 | 3.88490 |
| C | -8.23110 | 3.20950 | 4.30570 |
| H | -8.19530 | 3.60480 | 5.32910 |
| H | -7.22750 | 3.31380 | 3.87620 |
| H | -8.89610 | 3.86280 | 3.73580 |
| Pd | -6.59500 | 0.96520 | 8.27970 |
| P | -8.56630 | 0.88190 | 9.65210 |
| C | -8.61960 | -0.63080 | 10.85260 |
| C | -8.92310 | -1.91120 | 10.04520 |
| H | -8.73480 | -2.77760 | 10.68960 |
| H | -9.96280 | -1.97270 | 9.72100 |
| H | -8.27280 | -2.00490 | 9.17010 |
| C | -7.22090 | -0.84380 | 11.47210 |
| H | -7.27700 | -1.70590 | 12.14870 |
| H | -6.47050 | -1.05540 | 10.70950 |
| H | -6.85550 | 0.00510 | 12.04370 |
| C | -9.65870 | -0.50240 | 11.98570 |
| H | -9.66320 | -1.43570 | 12.56210 |
| H | -9.41520 | 0.30130 | 12.68180 |
| H | -10.67340 | -0.34050 | 11.62010 |
| C | -8.62900 | 2.52140 | 10.68350 |
| C | -10.13870 | 0.80430 | 8.50930 |
| C | -10.25380 | 2.13520 | 7.73430 |
| H | -11.04360 | 2.03590 | 6.98210 |

| | | | |
|---|---|---|---|
| H | -10.51140 | 2.98100 | 8.37270 |
| H | -9.33340 | 2.36930 | 7.19350 |
| C | -11.47320 | 0.53540 | 9.23440 |
| H | -12.28250 | 0.57080 | 8.49500 |
| H | -11.50120 | -0.45770 | 9.68680 |
| H | -11.70420 | 1.26950 | 10.00590 |
| C | -9.95640 | -0.29800 | 7.44080 |
| H | -10.71450 | -0.16400 | 6.66300 |
| H | -8.98850 | -0.23080 | 6.94580 |
| H | -10.06910 | -1.30480 | 7.83820 |
| C | -8.05720 | 3.65550 | 9.79710 |
| H | -8.10270 | 4.59480 | 10.36190 |
| H | -7.00950 | 3.46200 | 9.55150 |
| H | -8.60820 | 3.80380 | 8.86850 |
| C | -7.69110 | 2.42640 | 11.90650 |
| H | -7.63740 | 3.42080 | 12.36620 |
| H | -8.05960 | 1.74230 | 12.67230 |
| H | -6.67720 | 2.13900 | 11.62260 |
| C | -10.03230 | 2.91690 | 11.18310 |
| H | -10.48830 | 2.14420 | 11.80560 |
| H | -9.93930 | 3.81890 | 11.79990 |
| H | -10.72100 | 3.15590 | 10.37120 |
| Br | -4.66410 | 1.17060 | 10.03600 |
| H | -2.73410 | 0.88220 | 5.48720 |
| H | -3.75230 | 0.55940 | 4.06080 |
| C | -1.46590 | -1.13520 | 4.20980 |
| H | -1.53310 | -0.59690 | 3.25960 |
| H | -0.68910 | -0.65550 | 4.82990 |
| H | -1.14890 | -2.15810 | 3.98920 |

**Product resulting from *syn* Mizoroki-Heck pathway (Figure S11)**

| | | | |
|---|---|---|---|
| C | -5.72340 | -0.51620 | 4.96540 |
| C | -6.51090 | 0.79010 | 4.69780 |
| C | -4.57760 | -0.12580 | 5.94080 |
| H | -6.06050 | 1.31520 | 3.84070 |
| C | -3.26000 | 0.21710 | 5.18310 |
| C | -2.87650 | -3.50360 | 7.91760 |
| C | -4.04460 | -2.98920 | 8.48380 |
| C | -4.66390 | -1.86770 | 7.91310 |
| C | -4.09960 | -1.28600 | 6.78970 |
| C | -2.92510 | -1.80830 | 6.22130 |
| C | -2.29610 | -2.92060 | 6.78350 |

| | | | |
|---|---|---|---|
| H | -2.39640 | -4.36770 | 8.36900 |
| H | -4.46800 | -3.44970 | 9.37150 |
| H | -5.56650 | -1.44560 | 8.34250 |
| H | -1.38010 | -3.32390 | 6.36470 |
| H | -5.34330 | -0.99610 | 4.06190 |
| C | -5.13860 | 1.09010 | 6.62760 |
| H | -4.62480 | 1.60140 | 7.43500 |
| H | -6.36060 | -1.22360 | 5.48820 |
| N | -2.56280 | -1.07130 | 5.08490 |
| C | -6.19240 | 1.61300 | 5.94160 |
| H | -6.56510 | 2.61080 | 6.11420 |
| H | -7.50540 | -0.52220 | 7.11070 |
| N | -7.95060 | 0.65030 | 4.41650 |
| C | -8.80610 | 1.75170 | 4.33700 |
| C | -10.05910 | 1.29270 | 3.99980 |
| C | -9.97620 | -0.11220 | 3.84590 |
| C | -8.67530 | -0.49890 | 4.08740 |
| H | -10.93050 | 1.91940 | 3.86660 |
| H | -10.77300 | -0.78990 | 3.56990 |
| C | -8.17300 | -1.90600 | 3.96420 |
| H | -7.93410 | -2.37580 | 4.92560 |
| H | -8.96950 | -2.50080 | 3.50940 |
| H | -7.29190 | -2.00010 | 3.32230 |
| C | -8.39960 | 3.17840 | 4.54020 |
| H | -8.32000 | 3.46140 | 5.59690 |
| H | -7.44610 | 3.42190 | 4.05730 |
| H | -9.16190 | 3.82100 | 4.09240 |
| Pd | -7.25050 | 0.77070 | 7.87040 |
| P | -8.94490 | -0.19150 | 9.19220 |
| C | -8.39320 | -0.40720 | 11.03390 |
| C | -7.44050 | -1.61610 | 11.15400 |
| H | -7.00920 | -1.61140 | 12.16160 |
| H | -7.94400 | -2.57520 | 11.02510 |
| H | -6.60950 | -1.55060 | 10.44590 |
| C | -7.57360 | 0.82590 | 11.47250 |
| H | -7.28230 | 0.68310 | 12.52070 |
| H | -6.66660 | 0.93960 | 10.87690 |
| H | -8.11590 | 1.76520 | 11.40100 |
| C | -9.57010 | -0.60330 | 12.01300 |
| H | -9.15940 | -0.77230 | 13.01580 |
| H | -10.20850 | 0.27890 | 12.07420 |
| H | -10.19530 | -1.46250 | 11.76900 |
| C | -10.46150 | 1.01130 | 9.09080 |

| | | | |
|---|---|---|---|
| C | -9.48460 | -1.93130 | 8.50570 |
| C | -10.20660 | -1.73330 | 7.15610 |
| H | -10.36490 | -2.71820 | 6.70070 |
| H | -11.18660 | -1.26700 | 7.25700 |
| H | -9.61700 | -1.14090 | 6.45370 |
| C | -10.40440 | -2.72510 | 9.45930 |
| H | -10.74100 | -3.63080 | 8.94110 |
| H | -9.88230 | -3.05060 | 10.36110 |
| H | -11.29540 | -2.17420 | 9.75860 |
| C | -8.25400 | -2.81880 | 8.19890 |
| H | -8.61760 | -3.77500 | 7.80420 |
| H | -7.60940 | -2.37360 | 7.43930 |
| H | -7.63740 | -3.04110 | 9.06700 |
| C | -10.52850 | 1.56570 | 7.64720 |
| H | -11.39880 | 2.22960 | 7.57400 |
| H | -9.63940 | 2.15850 | 7.41770 |
| H | -10.62880 | 0.80690 | 6.87380 |
| C | -10.23940 | 2.23120 | 10.01040 |
| H | -11.04060 | 2.95140 | 9.80530 |
| H | -10.29960 | 1.98220 | 11.07120 |
| H | -9.28950 | 2.72990 | 9.81010 |
| C | -11.81290 | 0.36990 | 9.46350 |
| H | -11.81850 | -0.04000 | 10.47580 |
| H | -12.58200 | 1.15040 | 9.42590 |
| H | -12.12110 | -0.41380 | 8.77060 |
| Br | -6.78000 | 3.17360 | 8.92200 |
| H | -2.66480 | 0.94000 | 5.76910 |
| H | -3.44040 | 0.64570 | 4.19200 |
| C | -1.17930 | -1.03760 | 4.65370 |
| H | -1.10640 | -0.47500 | 3.71860 |
| H | -0.51130 | -0.56930 | 5.39690 |
| H | -0.82260 | -2.05270 | 4.45880 |

# References


(1) Sluder, A.; Shah, S.; Cassayre, J.; Clover, R.; Maienfisch, P.; Molleyres, L. P.; Hirst, E. A.; Flemming, A. J.; Shi, M.; Cutler, P.; et al. Spiroindolines Identify the Vesicular Acetylcholine Transporter as a Novel Target for Insecticide Action. *PLoS One.* **2012**, *7* (5), 1–13. https://doi.org/10.1371/journal.pone.0034712.

(2) Mizutani, A.; Yashiroda, Y.; Muramatsu, Y.; Yoshida, H.; Chikada, T.; Tsumura, T.; Okue, M.; Shirai, F.; Fukami, T.; Yoshida, M.; et al. RK-287107, a Potent and Specific Tankyrase Inhibitor, Blocks Colorectal Cancer Cell Growth in a Preclinical Model. *Cancer Sci.* **2018**, *109* (12), 4003–4014. https://doi.org/10.1111/cas.13805.

(3) Xia, Z. L.; Zheng, C.; Wang, S. G.; You, S. L. Catalytic Asymmetric Dearomatization of Indolyl Dihydropyridines through an Enamine Isomerization/Spirocyclization/Transfer Hydrogenation Sequence. *Angew. Chem. Int. Ed.* **2018**, *57* (10), 2653–2656. https://doi.org/10.1002/anie.201712435.

(4) Pan, Z.; Liu, Y.; Hu, F.; Liu, Q.; Shang, W.; Ji, X.; Xia, C. Enantioselective Synthesis of Spiroindolines via Cascade Isomerization/Spirocyclization/Dearomatization Reaction. *Org. Lett.* **2020**, 1589–1593. https://doi.org/10.1021/acs.orglett.0c00181.

(5) Zhu, M.; Zheng, C.; Zhang, X.; You, S. L. Synthesis of Cyclobutane-Fused Angular Tetracyclic Spiroindolines via Visible-Light-Promoted Intramolecular Dearomatization of Indole Derivatives. *J. Am. Chem. Soc.* **2019**, *141* (6), 2636–2644. https://doi.org/10.1021/jacs.8b12965.

(6) Zaman, M.; Hasan, M.; Peshkov, A. A.; Van Hecke, K.; Van der Eycken, E. V.; Pereshivko, O. P.; Peshkov, V. A. Silver(I) Triflate-Catalyzed Protocol for the Post-Ugi Synthesis of Spiroindolines. *Adv. Synth. Catal.* **2020**, *362* (1), 261–268. https://doi.org/10.1002/adsc.201901064.

(7) Liang, G.; Ji, Y.; Liu, H.; Pang, Y.; Zhou, B.; Cheng, M.; Liu, Y.; Lin, B.; Liu, Y. Silver Triflate/N-Fluorobenzenesulfonimide-Catalyzed Cycloisomerization of Tryptamine-Ynamide to Spiro[Indoline-3,4′-Piperidine] Induced by Cation-π-π Interactions between Substrate and Metal Ligand. *Adv. Synth. Catal.* **2020**, *362* (1), 192–205. https://doi.org/10.1002/adsc.201901175.

(8) Wu, K. J.; Dai, L. X.; You, S. L. Palladium(0)-Catalyzed Dearomative Arylation of Indoles: Convenient Access to Spiroindolenine Derivatives. *Org. Lett.* **2012**, *14* (14), 3772–3775. https://doi.org/10.1021/ol301663h.

(9) Roy, T.; Brandt, P.; Wetzel, A.; Bergman, J.; Brånalt, J.; Sävmarker, J.; Larhed, M. Selective Synthesis of Spirooxindoles by an Intramolecular Heck-Mizoroki Reaction. *Org. Lett.* **2017**, *19* (10), 2738–2741. https://doi.org/10.1021/acs.orglett.7b01094.

(10) Wetzel, A.; Bergman, J.; Brandt, P.; Larhed, M.; Branalt, J. Regio- and Stereoselective Synthesis of Functionalized Cyclopentene Derivatives via Mizoroki-Heck Reactions. *Org. Lett.* **2017**, *19* (7), 1602–1605. https://doi.org/10.1021/acs.orglett.7b00325.

(11) Grimme, S.; Antony, J.; Ehrlich, S.; Krieg, H. A Consistent and Accurate Ab Initio Parametrization of Density Functional Dispersion Correction (DFT-D) for the 94 Elements H-Pu. *J. Chem. Phys.* **2010**, *132* (15). https://doi.org/10.1063/1.3382344.

(12) Hay, P. J.; Wadt, W. R. Ab Initio Effective Core Potentials for Molecular Calculations. Potentials for

K to Au Including the Outermost Core Orbitale. *J. Chem. Phys.* **1985**, *82* (1), 299–310. https://doi.org/10.1063/1.448975.

(13) Marten, B.; Kim, K.; Cortis, C.; Friesner, R. A.; Murphy, R. B.; Ringnalda, M. N.; Sitkoff, D.; Honig, B. New Model for Calculation of Solvation Free Energies: Correction of Self-Consistent Reaction Field Continuum Dielectric Theory for Short-Range Hydrogen-Bonding Effects. *J. Phys. Chem.* **1996**, *100* (28), 11775–11788. https://doi.org/10.1021/jp953087x.

(14) Bochevarov, A. D.; Harder, E.; Hughes, T. F.; Greenwood, J. R.; Braden, D. A.; Philipp, D. M.; Rinaldo, D.; Halls, M. D.; Zhang, J.; Friesner, R. A. Jaguar: A High-Performance Quantum Chemistry Software Program with Strengths in Life and Materials Sciences. *Int. J. Quantum Chem.* **2013**, *113* (18), 2110–2142. https://doi.org/10.1002/qua.24481.

(15) Dolomanov, O. V.; Bourhis, L. J.; Gildea, R. J.; Howard, J. A. K.; Puschmann, H. OLEX2: A Complete Structure Solution, Refinement and Analysis Program. *J. Appl. Crystallogr.* **2009**, *42* (2), 339–341. https://doi.org/10.1107/S0021889808042726.

(16) Sheldrick, G. M. SHELXT - Integrated Space-Group and Crystal-Structure Determination. *Acta Crystallogr. Sect. A Found. Crystallogr.* **2015**, *71* (1), 3–8. https://doi.org/10.1107/S2053273314026370.

(17) Bourhis, L. J.; Dolomanov, O. V.; Gildea, R. J.; Howard, J. A. K.; Puschmann, H. The Anatomy of a Comprehensive Constrained, Restrained Refinement Program for the Modern Computing Environment - Olex2 Dissected. *Acta Crystallogr. Sect. A Found. Crystallogr.* **2015**, *71* (1), 59–75. https://doi.org/10.1107/S2053273314022207.
