# Supplementary material for: Diastereoselective Synthesis of N-Methylspiroindolines by Intramolecular Mizoroki–Heck Annulations
Source: ACS Omega. 2022 Aug 26;7(36):32525–35. doi: 10.1021/acsomega.2c04111 (PMC9476516; doi:10.1021/acsomega.2c04111)
Supplement: Supplementary file 4 — ao2c04111_si_004.pdf [file ao2c04111_si_004.pdf]

BW2546

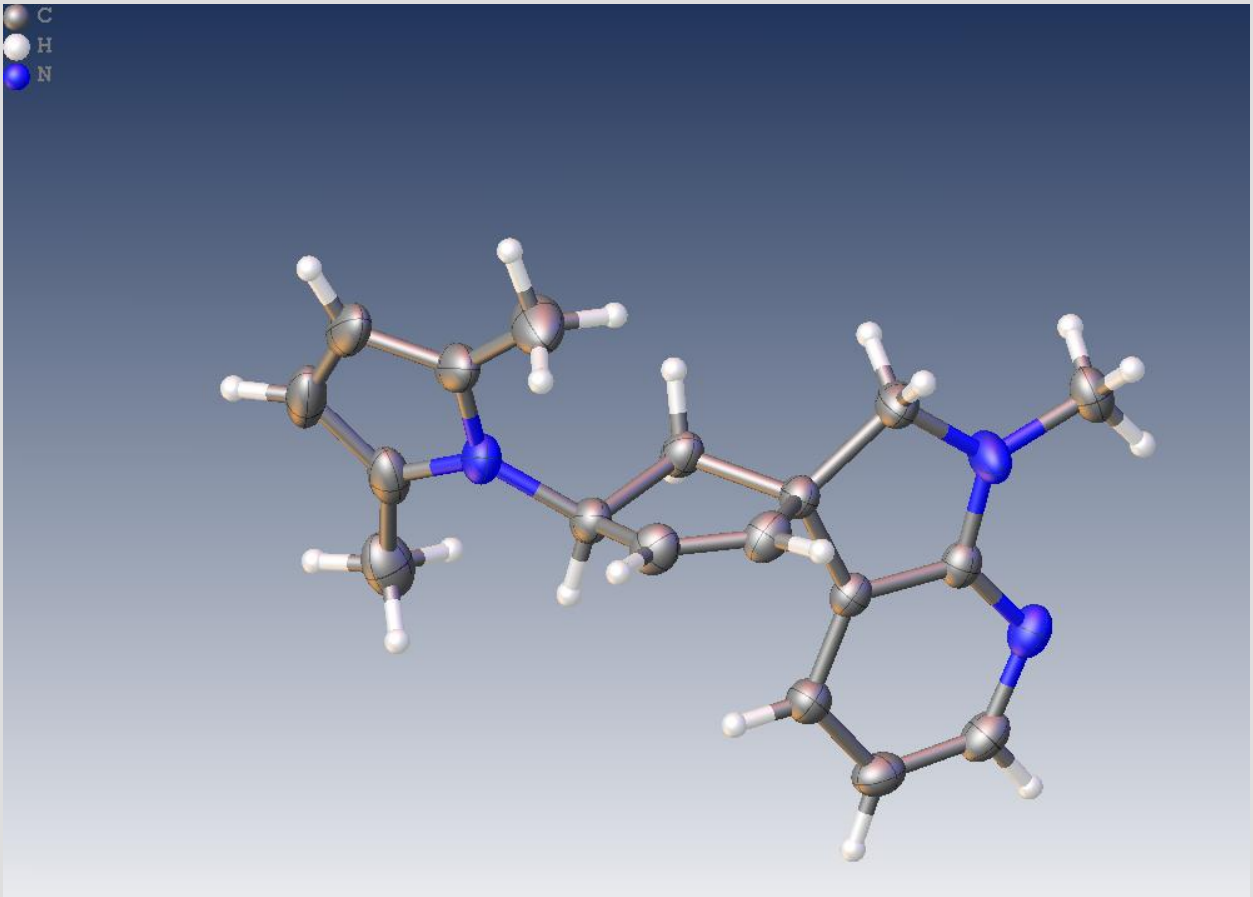

R_1 = 3.25%

wR_2 = 8.86%

**$C_{18}H_{21}N_3$**	
monoclinic, *C*2	
a/Å:	14.3755(4)
b/Å:	6.94300(17)
c/Å:	16.3630(4)
α/°:	90
β/°:	110.471(3)
γ/°:	90

Diffractometer Rigaku, XtaLAB Synergy, Dualflex, HyPix

Radiation Source Cu K_{α}(λ=1.54184Å)

Data Collection CrysAlisPro 1.171.41.112a (Rigaku OD, 2021)

Table 1 Crystal data and structure refinement for BW2546.

Identification code	BW2546
Empirical formula	$C_{18}H_{21}N_3$
Formula weight	279.39
Temperature/K	170.0(2)

Crystal system	monoclinic
Space group	*C*2
a/Å	14.3755(4)
b/Å	6.94300(17)
c/Å	16.3630(4)
α/°	90
β/°	110.471(3)
γ/°	90
Volume/Å^3	1530.04(7)
Z	4
ρ_{calc}g/cm^3	1.2128
μ/mm^{-1}	0.562
F(000)	600.0
Crystal size/mm^3	0.14 × 0.13 × 0.03
Radiation	Cu Kα (λ = 1.54184)
2Θ max. for data collection/°	155.0
Index ranges	$-13 \leq h \leq 18$, $-8 \leq k \leq 8$, $-19 \leq l \leq 20$
Reflections collected	9236
Independent reflections	2989 [R_{int} = 0.0251, R_{sigma} = 0.0199]
Data/restraints/parameters	2989/1/197
Goodness-of-fit on F^2	1.068
Final *R* indexes [*I*>=2σ(*I*)]	R_1 = 0.0323, wR_2 = 0.0844
Final *R* indexes [all data]	R_1 = 0.0330, wR_2 = 0.0861
Largest diff. peak/hole / e Å^{-3}	0.11/-0.18
Flack's *x* parameter	0.1(2)

Table 2 Fractional Atomic Coordinates (×10^4) and Equivalent Isotropic Displacement Parameters (Å^2×10^3) for BW2546. U_{eq} is defined as 1/3 of of the trace of the orthogonalised U_{IJ} tensor.

**Atom**	***x***	***y***	***z***	***U*(eq)**
C1	2871.7(8)	6311(2)	3358.0(7)	27.5(2)
C2	2942.2(10)	8003(2)	2798.4(10)	33.2(3)
C3	2889.6(11)	7479(2)	2007.9(9)	35.3(3)
C4	2763.7(10)	5333(2)	1886.8(9)	31.2(3)

C5	3064.2(10)	4601(2)	2833.5(9)	30.8(3)
N1'	2954.3(8)	6405(2)	4847.7(6)	41.2(3)
C1'	3351.3(10)	6318(3)	5784.2(8)	38.7(3)
C2'	3581.5(9)	6366(3)	4327.6(8)	35.9(3)
C3A'	1860.3(8)	6251(2)	3457.8(7)	27.0(2)
C4'	926.1(9)	6180(2)	2853.8(8)	33.5(3)
C5'	125.6(9)	6149(2)	3158.2(9)	37.5(3)
C6'	315.9(9)	6179(2)	4037.4(9)	35.9(3)
N7'	1234.9(8)	6256.1(19)	4658.5(7)	34.4(2)
C7A'	1976.9(8)	6293(2)	4346.0(7)	27.8(2)
N1P	3298.4(8)	4392.4(18)	1381.2(7)	35.3(3)
C2P	4263.4(10)	4739(3)	1425.1(9)	41.4(3)
C3P	4484.0(13)	3395(3)	906.7(10)	49.3(4)
C4P	3653.3(14)	2195(2)	552.1(10)	50.3(4)
C5P	2923.5(12)	2809(2)	850.6(9)	40.4(3)
C6P	4898.2(11)	6332(4)	1937.1(10)	58.0(4)
C7P	1914.3(14)	2010(2)	686.4(11)	50.2(4)

Table 3 Anisotropic Displacement Parameters (Å^2×10^3) for BW2546. The Anisotropic displacement factor exponent takes the form: $-2\pi^2[h^2a^{*2}U_{11}+2hka^*b^*U_{12}+\ldots]$.

**Atom**	U_{11}	U_{22}	U_{33}	U_{12}	U_{13}	U_{23}
C1	27.0(5)	32.1(5)	25.6(5)	0.6(6)	11.9(4)	-0.6(6)
C2	36.1(6)	28.0(6)	41.1(7)	-2.7(5)	20.5(6)	-0.9(5)
C3	40.4(7)	35.1(7)	35.4(7)	2.6(6)	19.5(6)	7.7(5)
C4	32.0(6)	37.3(7)	27.1(6)	3.3(5)	14.0(5)	1.1(5)
C5	34.6(6)	31.9(6)	29.2(6)	5.5(5)	15.4(5)	2.7(5)
N1'	33.8(5)	66.8(8)	23.9(5)	-5.4(6)	11.3(4)	-4.0(6)
C1'	48.9(7)	38.9(6)	25.6(6)	-2.6(7)	9.8(5)	-2.3(7)
C2'	29.4(5)	49.4(7)	30.4(6)	-0.5(6)	12.2(4)	-5.5(7)
C3A'	28.4(5)	25.8(5)	29.7(5)	1.2(5)	13.8(4)	-0.1(6)
C4'	32.0(6)	38.0(6)	30.9(6)	3.1(6)	11.5(4)	-0.6(6)
C5'	26.8(5)	40.5(7)	45.0(7)	2.0(6)	12.5(5)	-1.8(7)
C6'	34.3(6)	34.7(6)	46.9(7)	-0.4(6)	24.4(5)	-0.1(7)
N7'	38.8(5)	35.9(5)	36.4(5)	-1.2(5)	23.1(4)	-1.1(5)
C7A'	33.4(5)	25.3(5)	28.3(5)	0.4(5)	15.2(4)	0.3(5)
N1P	38.9(6)	43.5(6)	26.2(5)	3.2(5)	14.7(4)	-3.2(5)
C2P	38.5(7)	62.6(9)	26.6(6)	8.6(7)	15.7(5)	3.5(6)
C3P	55.3(9)	70.0(11)	29.1(7)	23.4(8)	23.0(6)	7.2(7)
C4P	74.3(10)	49.1(8)	30.4(7)	19.5(8)	21.9(7)	-1.2(6)
C5P	58.2(8)	36.9(7)	24.8(6)	6.0(6)	13.0(6)	0.2(5)
C6P	39.3(7)	93.9(13)	46.3(8)	-9.2(10)	21.9(6)	-12.9(11)
C7P	66.1(10)	39.2(8)	41.2(8)	-6.0(7)	13.5(7)	-5.4(6)

Table 4 Bond Lengths for BW2546.

Atom	Atom	Length/Å	Atom	Atom	Length/Å
C1	C2	1.5148(19)	C3A'	C7A'	1.4039(15)
C1	C5	1.5455(18)	C4'	C5'	1.4050(16)
C1	C2'	1.5580(16)	C5'	C6'	1.3673(19)
C1	C3A'	1.5192(14)	C6'	N7'	1.3572(17)
C2	C3	1.320(2)	N7'	C7A'	1.3347(14)
C3	C4	1.5056(18)	N1P	C2P	1.3849(18)
C4	C5	1.5416(18)	N1P	C5P	1.3875(19)
C4	N1P	1.4650(16)	C2P	C3P	1.371(2)
N1'	C1'	1.4372(15)	C2P	C6P	1.491(3)
N1'	C2'	1.4404(14)	C3P	C4P	1.404(3)
N1'	C7A'	1.3592(15)	C4P	C5P	1.371(2)
C3A'	C4'	1.3617(16)	C5P	C7P	1.487(2)

Table 5 Bond Angles for BW2546.

Atom	Atom	Atom	Angle/°	Atom	Atom	Atom	Angle/°
C5	C1	C2	101.38(8)	C5'	C4'	C3A'	117.74(11)
C2'	C1	C2	115.99(12)	C6'	C5'	C4'	119.05(11)
C2'	C1	C5	113.80(11)	N7'	C6'	C5'	124.93(10)
C3A'	C1	C2	110.79(10)	C7A'	N7'	C6'	114.39(10)
C3A'	C1	C5	113.75(11)	C3A'	C7A'	N1'	110.51(9)
C3A'	C1	C2'	101.64(8)	N7'	C7A'	N1'	124.44(10)
C3	C2	C1	112.66(12)	N7'	C7A'	C3A'	125.04(10)
C4	C3	C2	111.41(12)	C2P	N1P	C4	127.38(12)
C5	C4	C3	102.44(12)	C5P	N1P	C4	122.73(12)
N1P	C4	C3	116.92(12)	C5P	N1P	C2P	109.45(12)
N1P	C4	C5	113.74(11)	C3P	C2P	N1P	107.09(15)
C4	C5	C1	105.26(10)	C6P	C2P	N1P	124.28(13)
C2'	N1'	C1'	122.14(10)	C6P	C2P	C3P	128.61(14)
C7A'	N1'	C1'	125.64(10)	C4P	C3P	C2P	108.19(14)
C7A'	N1'	C2'	111.74(9)	C5P	C4P	C3P	108.33(14)
N1'	C2'	C1	106.25(9)	C4P	C5P	N1P	106.92(14)
C4'	C3A'	C1	131.37(10)	C7P	C5P	N1P	123.33(13)
C7A'	C3A'	C1	109.78(9)	C7P	C5P	C4P	129.74(15)
C7A'	C3A'	C4'	118.85(10)				

Table 6 Hydrogen Atom Coordinates (Å×10^4) and Isotropic Displacement Parameters (Å^2×10^3) for BW2546.

Atom	*x*	*y*	*z*	*U*(eq)
H2	3016.2(10)	9301(2)	2994.9(10)	39.8(3)
H3	2926.5(11)	8345(2)	1570.9(9)	42.4(4)
H4	2045(13)	5020(20)	1557(11)	35(4)
H5a	3773.7(10)	4228(2)	3060.7(9)	36.9(3)

H5b	2658.2(10)	3473(2)	2867.2(9)	36.9(3)
H1'a	2805.0(11)	6320(20)	6012.0(9)	58.0(4)
H1'b	3742(9)	5137(10)	5967.6(8)	58.0(4)
H1'c	3777(9)	7439(11)	6011.4(9)	58.0(4)
H2'a	4013.0(9)	5211(3)	4467.5(8)	43.1(3)
H2'b	4007.2(9)	7527(3)	4439.0(8)	43.1(3)
H4'	819.4(9)	6153(2)	2247.2(8)	40.2(3)
H5'	-538.4(9)	6108(2)	2758.9(9)	44.9(3)
H6'	-236.9(9)	6143(2)	4227.8(9)	43.1(3)
H3P	5093.5(13)	3293(3)	805.0(10)	59.2(5)
H4P	3605.3(14)	1139(2)	170.6(10)	60.4(5)
H6Pa	4657(7)	7564(4)	1649(5)	87.0(7)
H6Pb	4869(9)	6348(14)	2527(3)	87.0(7)
H6Pc	5586(2)	6129(12)	1972(8)	87.0(7)
H7Pa	1855(4)	1611(19)	1240.5(16)	75.4(6)
H7Pb	1415.4(14)	2998(7)	413(8)	75.4(6)
H7Pc	1808(4)	895(12)	297(8)	75.4(6)

Experimental

Single crystals of $C_{18}H_{21}N_3$ [BW2546] were investigated with the help of on a Rigaku, XtaLAB Synergy, Dualflex, HyPix diffractometer. The crystal was kept at 170.0(2) K during data collection. Using Olex2 [1], the structure was solved with the SHELXT [2] structure solution program using Intrinsic Phasing and refined with the olex2.refine [3] refinement package using Levenberg-Marquardt minimisation.

1. Dolomanov, O.V., Bourhis, L.J., Gildea, R.J, Howard, J.A.K. & Puschmann, H. (2009), J. Appl. Cryst. 42, 339-341.

2. Sheldrick, G.M. (2015). Acta Cryst. A71, 3-8.

3. Bourhis, L.J., Dolomanov, O.V., Gildea, R.J., Howard, J.A.K., Puschmann, H. (2015). Acta Cryst. A71, 59-75.

Crystal Data for $C_{18}H_{21}N_3$ (M = 279.39 g/mol): monoclinic, space group $C2$ (no. 5), a = 14.3755(4) Å, b = 6.9430(2) Å, c = 16.3630(4) Å, β = 110.471(3)°, V = 1530.04(7) Å^3, Z = 4, T = 170.0(2) K, μ(Cu Kα) = 0.562 mm^{-1}, $Dcalc$ = 1.2128 g/cm^3, 9236 reflections measured (2Θ ≤ 155.0°), 2989 unique (R_{int} = 0.0251, R_{sigma} = 0.0199) which were used in all calculations. The final R_1 was 0.0323 ($I > 2\sigma(I)$) and wR_2 was 0.0861 (all data).

Details:

```
1. Fixed Uiso
 At 1.2 times of:
  All C(H) groups, All C(H,H) groups
 At 1.5 times of:
  All C(H,H,H) groups
2.a Secondary CH2 refined with riding coordinates:
 C5(H5a,H5b), C2'(H2'a,H2'b)
2.b Aromatic/amide H refined with riding coordinates:
```

 C2(H2), C3(H3), C4'(H4'), C5'(H5'), C6'(H6'), C3P(H3P), C4P(H4P)
2.c Idealised Me refined as rotating group:
 C1'(H1'a,H1'b,H1'c), C6P(H6Pa,H6Pb,H6Pc), C7P(H7Pa,H7Pb,H7Pc)

This report has been created with Olex2, compiled on 2018.05.17 svn.r3504 for Rigaku Oxford Diffraction.
