# Supplementary figures and images for: Diastereoselective Synthesis of N-Methylspiroindolines by Intramolecular Mizoroki–Heck Annulations
Source: ACS Omega. 2022 Aug 26;7(36):32525–35. doi: 10.1021/acsomega.2c04111 (PMC9476516; doi:10.1021/acsomega.2c04111)

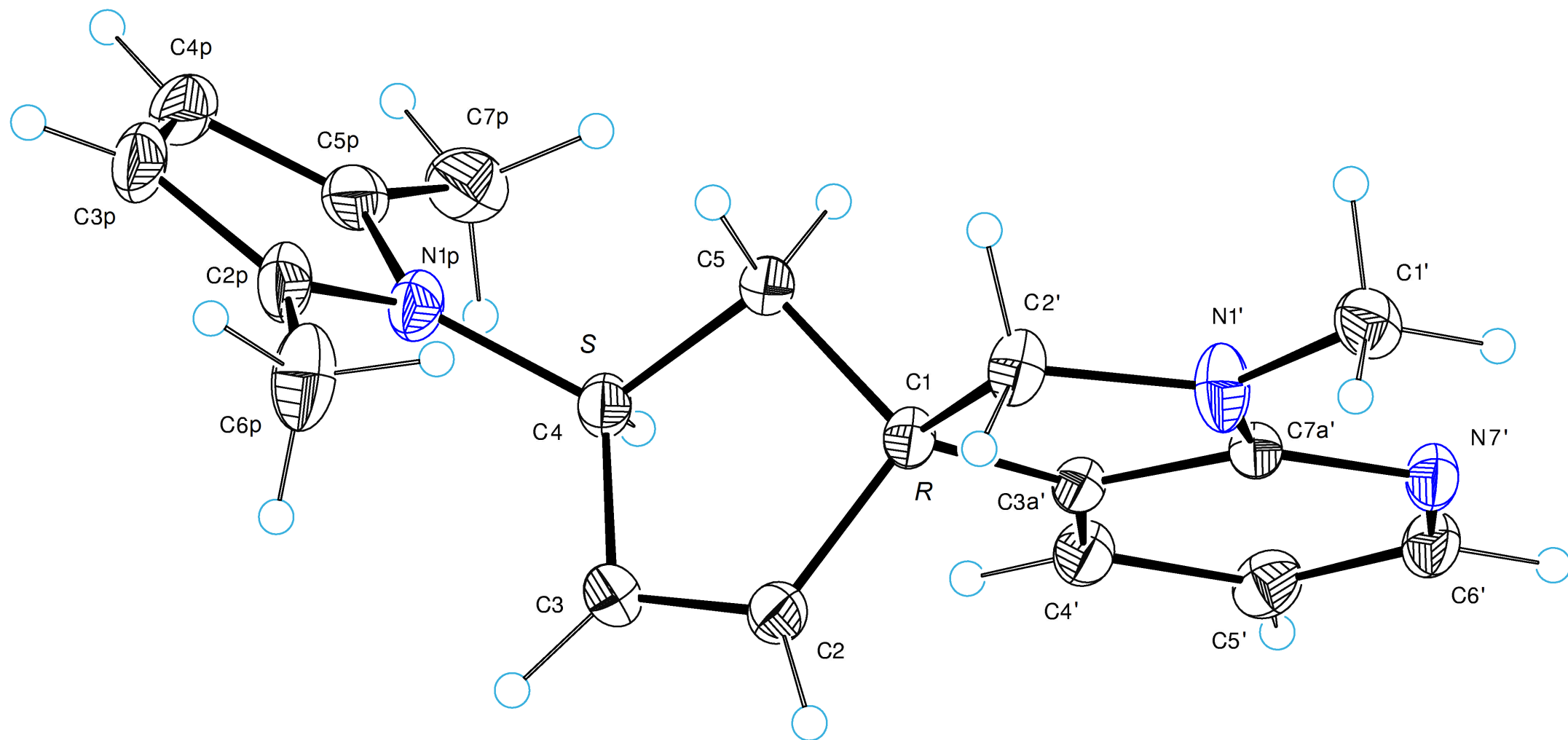

Supplement: Supplementary file 5 — ao2c04111_si_005.pdf [file ao2c04111_si_005.pdf]
